# Supplementary material for: Development and content of a community-based reablement programme (I-MANAGE): a co-creation study
Source: BMJ Open. 2023 Aug 30;13(8):e070890. doi: 10.1136/bmjopen-2022-070890 (PMC10471872; doi:10.1136/bmjopen-2022-070890)
Supplement: Supplementary data [file bmjopen-2022-070890supp002.pdf]

Supplementary file 2. Overview of the different stakeholders involved per research activity.

**Stakeholders**[a] (n = 26)	**Observations**[b]	**Individual interviews**	**Working groups**								
			**Development of a logic model**[c]			**Translation to practice**[d]					
			1	2	3	1	2	3	4	5	6
Domestic support worker	•		•	•	•		•			•	
Registered nurse (n = 3)	•		•	•	•	•	•		•	•	
Nursing assistant	•										
Physiotherapist (n = 4)		•	•	•	•	•				•	
Expert in the field of GR research (n = 3)		•	•								
Informal caregiver (n = 3)		•									•
Policy maker of the local municipality (n = 2)		•		•	•			•	•		
Client representative		•	•	•					•		
Informal caregiver representative		•	•	•	•						
Occupational therapist (n = 2)			•	•	•	•	•	•			
Psychologist			•	•	•						•
Geriatrician						•					
Reablement researcher							•				
Manager community care team										•	
Consultant informal care									•		•

GR = geriatric rehabilitation

Note: [a] if n > 1, not all stakeholders participated in every indicated research activity for that group; [b] observations were performed by the researchers; [c] indicates the three consecutive working group sessions throughout which the logic model was being developed following step 1 to 6 as described by Bleijenberg et al. [1]; [d] indicated the 6 working groups related to each programme component described in the logic model (1 = improving assessment and goal-setting, 2 = stimulating self-management during meaningful daily activities, 3 = optimising the use of the physical environment, 4 = optimising the use of the social environment, 5 = improving interprofessional collaboration, and 6 = engaging the informal caregiver).

References

1. Bleijenberg N, de Man-van Ginkel JM, Trappenburg JCA, et al. Increasing value and reducing waste by optimizing the development of complex interventions: Enriching the development phase of the Medical Research Council (MRC) Framework. Int J Nurs Stud. 2018;79:86-93.
