# Supplementary material for: Development and content of a community-based reablement programme (I-MANAGE): a co-creation study
Source: BMJ Open. 2023 Aug 30;13(8):e070890. doi: 10.1136/bmjopen-2022-070890 (PMC10471872; doi:10.1136/bmjopen-2022-070890)
Supplement: Supplementary data [file bmjopen-2022-070890supp003.pdf]

Supplementary file 3. Overview of the intermediate results of the first five steps of the development phase.

Programme development step	Results
1. Problem identification	The identified problems were situated in three main areas:<br>1. A lack of attention to meaningful daily activities. According to the working group, older persons were mainly focused on functional goals related to (I)ADL tasks rather than meaningful daily activities that increase their well-being. Additionally, the focus of care is often on eliminating diseases and symptoms instead of maintaining quality of life [1, 2];<br>2. There appeared a gap between the person's abilities, needs, and wishes, and the environment they reside in. When older people experience a deterioration in health status, their environment is often not adapted, i.e. the necessary home modifications and assistive devices are not always in place [3, 4];<br>3. Communication and coordination within the care trajectory of an older person. There is insufficient communication amongst different care professionals but also with older persons and informal caregivers [4-7].
2. Identifying the evidence	The identification of the evidence-based practices was mainly based on literature research. A literature review was performed to gain insight into current evidence on effective reablement programmes aimed at improving the independent living of older persons [8]. We specifically sought to gain a general understanding of different reablement programmes, their characteristics (e.g. duration), content, outcome measures, and effectiveness. Thirteen programmes were identified, all intended for (I)ADL-impaired community-dwelling older adults. The most common features were: 1) delivery by a trained and coordinated multidisciplinary team and 2) implementation of an intake assessment and goal-setting tool. The applied interventions focused on supporting (I)ADL and physical functioning, and improving home safety. Nine programmes showed a statistically significant improvement on at least one of the outcome measures (e.g. (I)ADL functioning).
3. Identifying or developing theory	A literature review was performed on the concept of disability and its working mechanisms [9] to identify a solid theoretical foundation for our programme (*step 3*). We concluded that by optimising the use of personal, as well as environmental, resources and focusing on health and functioning, rather than disability, an older person's independence and wellbeing could be improved, especially while performing meaningful daily activities in accordance with the person's needs and preferences.
4. Determine the needs	The determined needs are closely related to the identified problems. Stakeholders expressed the need for:<br>1. Standardised goal-setting at the beginning of the care process. Goals should be meaningful, embedded in the older person's own environment, and not solely focused on (I)ADL;<br>2. Timely assessment and, if necessary, adaptations to the home environment of the older person. This concerns adaptations to the built environment, as well as the availability of assistive devices. Additionally, the social environment of the older person needs to be assessed in order to make optimal use of it or provide additional support if the social network is rather weak;<br>3. Improved communication and coordination within the care trajectory of the older person. For example, a smooth handover of information between care professionals and organisations, involvement of the informal caregiver when discussing a client's care needs, multidisciplinary collaboration with clear roles for each provider, and the appointment of a care coordinator.
5. Examining current practice and context	When examining the current practice and context we focused on positive aspects and possible barriers for implementation. Possible barriers could be different financial arrangements and different communication systems within a multidisciplinary team. Positive aspects include the comprehensive assessment by community nurses at the start

	of receiving community care, a signalling role for domestic support workers, and the presence of various welfare initiatives in the Netherlands. A suggestion was made to include the principles of Positive Health [10] in the programme, a best practice that is already known and applied by some care professionals.

References

1. Nicholson C, Meyer J, Flatley M, et al. The experience of living at home with frailty in old age: A psychosocial qualitative study. Int J Nurs Stud. 2013 2013/09/01/;50(9):1172-9.
2. Aspinal F, Glasby J, Rostgaard T, et al. New horizons: Reablement - supporting older people towards independence. Age Ageing. 2016;45(5):572-6.
3. Q-Consult zorg. Inventarisatie zorginhoud GRZ: Het net opgehaald na zes jaar GRZ in de Zorgverzekeringswet. Q-Consult zorg, 2019.
4. Rustad EC, Furnes B, Cronfalk BS, et al. Older patients' experiences during care transition. Patient Prefer Adherence. 2016;10:769-79.
5. Everink IHJ, van Haastregt JCM, Kempen GIJM, et al. Uitdagingen in de geriatrische revalidatiezorg: de ontwikkeling van een zorgpad. Tijdschr Gerontol Geriatr. 2015 2015/04/01;46(2):104-12.
6. Bauer M, Fitzgerald L, Haesler E, et al. Hospital discharge planning for frail older people and their family. Are we delivering best practice? A review of the evidence. J Clin Nurs. 2009;18(18):2539-46.
7. Adelman RD, Tmanova LL, Delgado D, et al. Caregiver burden: a clinical review. JAMA. 2014;311(10):1052-60.
8. Mouchaers I, Van Haastregt JCM, Verbeek H, et al. « Reablement » comme outil thérapeutique pour offrir aux personnes âgées une vie de qualité à domicile après une réadaptation gériatrique. Rev Med Brux. 2021;42(4):324-32.
9. Mouchaers I, Verbeek H, Kempen GIJM, et al. The concept of disability and its causal mechanisms in older people over time from a theoretical perspective: a literature review. Eur J Ageing. 2022 2022/01/29.
10. Huber M, van Vliet M, Giezenberg M, et al. Towards a ‘patient-centred’ operationalisation of the new dynamic concept of health: a mixed methods study. BMJ Open. 2016;6(1):e010091.
