# Supplementary material for: Development and content of a community-based reablement programme (I-MANAGE): a co-creation study
Source: BMJ Open. 2023 Aug 30;13(8):e070890. doi: 10.1136/bmjopen-2022-070890 (PMC10471872; doi:10.1136/bmjopen-2022-070890)
Supplement: Author's manuscript [file bmjopen-2022-070890.draft_revisions.pdf]

BMJ Open is committed to open peer review. As part of this commitment we make the peer review history of every article we publish publicly available.

When an article is published we post the peer reviewers' comments and the authors' responses online. We also post the versions of the paper that were used during peer review. These are the versions that the peer review comments apply to.

The versions of the paper that follow are the versions that were submitted during the peer review process. They are not the versions of record or the final published versions. They should not be cited or distributed as the published version of this manuscript.

BMJ Open is an open access journal and the full, final, typeset and author-corrected version of record of the manuscript is available on our site with no access controls, subscription charges or pay-per-view fees (http://bmjopen.bmj.com).

If you have any questions on BMJ Open's open peer review process please email info.bmjopen@bmj.com

BMJ Open

Development, rationale and content of a community-based reablement programme (I-MANAGE): A co-creation study

Journal:	*BMJ Open*
Manuscript ID	bmjopen-2022-070890
Article Type:	Original research
Date Submitted by the Author:	07-Dec-2022

Complete List of Authors:	Mouchaers, Ines; Maastricht University, Department of Health Services Research; Living Lab of Ageing and Long Term Care<br>Verbeek, Hilde ; Maastricht University, Department of Health Services Research; Living Lab of Ageing and Long Term Care<br>Kempen, Gertrudis; Maastricht University, Department of Health Services Research; Living Lab of Ageing and Long Term Care<br>van Haastregt, Jolanda; Maastricht University, Department of Health Services Research; Living Lab of Ageing and Long Term Care<br>Vlaeyen, Ellen; KU Leuven, Public Health and Primary Care; UHasselt, Faculty of Medicine and Life Sciences<br>Goderis, Geert; KU Leuven, Department of Public Health and Primary Care<br>Metzelthin, Silke; Maastricht University, Department of Health Services Research; Living Lab of Ageing and Long Term Care

Keywords:	GERIATRIC MEDICINE, PRIMARY CARE, REHABILITATION MEDICINE

SCHOLARONE™
Manuscripts

I, the Submitting Author has the right to grant and does grant on behalf of all authors of the Work (as defined in the below author licence), an exclusive licence and/or a non-exclusive licence for contributions from authors who are: i) UK Crown employees; ii) where BMJ has agreed a CC-BY licence shall apply, and/or iii) in accordance with the terms applicable for US Federal Government officers or employees acting as part of their official duties; on a worldwide, perpetual, irrevocable, royalty-free basis to BMJ Publishing Group Ltd ("BMJ") its licensees and where the relevant Journal is co-owned by BMJ to the co-owners of the Journal, to publish the Work in this journal and any other BMJ products and to exploit all rights, as set out in our licence.

The Submitting Author accepts and understands that any supply made under these terms is made by BMJ to the Submitting Author unless you are acting as an employee on behalf of your employer or a postgraduate student of an affiliated institution which is paying any applicable article publishing charge ("APC") for Open Access articles. Where the Submitting Author wishes to make the Work available on an Open Access basis (and intends to pay the relevant APC), the terms of reuse of such Open Access shall be governed by a Creative Commons licence – details of these licences and which Creative Commons licence will apply to this Work are set out in our licence referred to above.

Other than as permitted in any relevant BMJ Author's Self Archiving Policies, I confirm this Work has not been accepted for publication elsewhere, is not being considered for publication elsewhere and does not duplicate material already published. I confirm all authors consent to publication of this Work and authorise the granting of this licence.

For peer review only

TITLE PAGE

Title

Development, rationale and content of a community-based reablement programme (I-MANAGE): A co-creation study

Authors

Ines Mouchaers, MSc, [1, 2, 3 *]

Hilde Verbeek, PhD, [1, 2]

Gertrudis I.J.M. Kempen, PhD, [1, 2]

Jolanda C.M. van Haastregt, PhD, [1, 2]

Ellen Vlaeyen, PhD, [3, 5, 6]

Geert Goderis, PhD, [4]

Silke F. Metzelthin, PhD, [1, 2]

Author Affiliations

[1] Department of Health Services Research, Faculty of Health Medicine and Life Sciences, CAPHRI Care and Public Health Research Institute, Maastricht University, Maastricht, the Netherlands

[2] Living Lab of Ageing and Long Term Care, Maastricht, the Netherlands

[3] Department of Public Health and Primary Care, Academic Centre for Nursing and Midwifery, KU Leuven, Leuven, Belgium

[4] Department of Public Health and Primary Care, Academic Centre for General Practice, KU Leuven, Leuven, Belgium

[5] Faculty of Medicine and Life Sciences, Hasselt University, Hasselt, Belgium

[6] University Centre for Nursing and Midwifery, Department of Public Health and Primary Care, Faculty of Medicine and Health Sciences, Ghent University, Belgium

For peer review only - http://bmjopen.bmj.com/site/about/guidelines.xhtml

Corresponding author

Ines Mouchaers: https://orcid.org/0000-0001-7189-4468

* Address correspondence to: Ines Mouchaers, MSc, Department of Health Services Research, P.O. Box 616, 6200 MD Maastricht, The Netherlands.

E-mail: i.mouchaers@maastrichtuniversity.nl; Telephone number: +31-(0)43 388 28 15.

Word count

3996

For peer review only

ABSTRACT

Objectives: With increasing age, people generally start experiencing problems related to independent living, resulting in an increased need for long-term care services. Investing in sustainable solutions to promote independent living is therefore essential. A concept attracting growing interest herein is reablement, a person-centred, holistic approach that promotes older adults' active participation through daily, social, leisure, and physical activities. To aid the implementation of reablement, a blue print was developed as part of the TRANS-SENIOR international training and research network. This paper describes the rationale, development, and content of I-MANAGE, a reablement programme developed for community-dwelling older adults.

Design: The development of the programme was performed according to the Medical Research Council framework. A co-creation design was used, including literature research, observations, interviews, and working group sessions with stakeholders.

Setting and participants: The interviews and working group sessions took place Dutch long-term home care context. Stakeholders invited to the individual interviews and working group sessions included care professionals, policymakers, client representatives, informal caregiver representatives, informal caregivers, and scientific experts.

Results: The co-creation process resulted in a 5-phase interdisciplinary primary care programme, called I-MANAGE. The programme focuses on improving the self-management and well-being of older people by working towards their meaningful goals. During the programme, the person's physical and social environment will be put to optimal use, and sufficient support will be provided to informal caregivers to reduce their burden. Lastly, the programme aims for continuity of care and better communication and coordination.

Conclusion: This paper provides a blueprint of the I-MANAGE programme, making it suitable for implementation in different local settings with the integration of their own best practices and available resources. If the programme is implemented as described, it is important to closely monitor the process and results.

For peer review only - http://bmjopen.bmj.com/site/about/guidelines.xhtml

For peer review only

STRENGTHS AND LIMITATIONS OF THIS STUDY

- Intervention development was conducted using a systematic approach based on the Medical Research Council framework.
- The intervention and its development is theory- and evidence-based as a result of extensive literature research.
- All end-users were represented during the development process, increasing its acceptability and feasibility.
- Data triangulation was used during the development process, increasing validity of the results.
- Client and informal caregiver representatives were included in the working group instead of clients and informal caregivers themselves, possibly missing an important voice.

For peer review only

For peer review only - http://bmjopen.bmj.com/site/about/guidelines.xhtml

BACKGROUND

Older people often face various challenges in living independently, due to trauma, acute or chronic illness [1, 2]. Disabilities in later life are often multiple and severe, with 50% of people aged 85 years or older requiring care and/or support with daily activities [3], resulting in an increased need for long-term care services. These challenges are even bigger if older people live in an environment that is not safe and does not meet their needs [4]. Previous research showed that a maladjusted environment negatively affects disability [5], which could lead to an accumulation of health risks, loss of independence, poor quality of life (QoL), and depression [6, 7]. Care and service delivery in the community is often fragmented [8], with little coordination and poor communication amongst care providers, clients, and informal caregivers [4, 8-10]. Moreover, the focus of care is often on eliminating specific diseases and symptoms instead of supporting the remaining capacity to maintain quality of life and independent living [11, 12]. Care professionals often tend to take over care tasks rather than performing tasks with their clients [12]. Not tackling these challenges properly could lead to increased healthcare utilisation and costs [13], and unnecessary (re)hospitalisations or permanent nursing home placement, each with their own risks (e.g. increased mortality) [14], while financial and workforce resources are shrinking [8, 13, 15]. Therefore, it is essential to invest in sustainable solutions to promote independent living [16-18].

A concept attracting growing interest in promoting independent living among older people is reablement, a person-centred, holistic approach that promotes older adults' active participation in daily activities through social, leisure, and physical activities chosen by the older person in line with their preferences, either at home or in the community [19]. Instead of creating dependency by taking over tasks, care professionals identify the capabilities and opportunities of individuals to maximise their independence by supporting them to achieve their goals, through participation in daily activities, home modifications and assistive devices, and involvement of their social network [12, 19-21]. Current evidence on the effectiveness of reablement interventions is inconclusive [12]. However, several systematic reviews have indicated that reablement has positive results related to activities of daily

For peer review only - http://bmjopen.bmj.com/site/about/guidelines.xhtml

living (ADL) functioning and health-related QoL [22-24]. Furthermore, despite the promising results abroad, this is no indication that it is also effective in its current format in the Dutch home care setting due to contextual differences. To implement reablement in the Dutch home care setting, existing programmes need to be revised and adapted to suit the current context. However, the development and content of community care programmes, such as reablement programmes, are often insufficiently described in the scientific literature [20, 25-27], offering little guidance for replication or to build on previous findings, despite being essential for the development of new and implementation of existing programmes in different settings [26-28].

Therefore, this paper describes the development, rationale, and content of I-MANAGE, a reablement programme for community-dwelling older adults. The programme was developed using a co-creation design involving stakeholders and reviewing the literature. We started with the development of a programme logic model, which was eventually translated to and applied in practice. The programme aims to improve older persons' self-management and participation in daily life, while also increasing quality of life and decreasing informal caregivers' burden. The ultimate goal is to ensure that older people can remain living at home independently and to avoid unnecessary transitions to institutional care. The programme is specifically suitable for the Dutch community care setting. Due to its extensive description, this manuscript may serve as a blueprint for implementation in other local settings.

For peer review only - http://bmjopen.bmj.com/site/about/guidelines.xhtml

METHODS

To describe the development process of the programme and ensure completeness of reporting, we used the guidance for reporting for intervention development studies in health research (GUIDED) (Supplemetary file 1) [26]. This checklist provides a clear and structured basis for the reporting of programme development, as well as the description of the content of the programme.

Design

I-MANAGE was developed between September 2019 and June 2021 using a co-creation design. The programme was developed following the first phase (development) of the Medical Research Council (MRC) framework for the development and evaluation of complex interventions [29, 30]. A logic model was developed using six iterative programme development steps: 1) identifying the problem, 2) identifying the evidence, 3) identifying or developing a theory, 4) determining needs, 5) examining current practices and the context, and 6) modelling processes and outcomes [31]. The logic model was then translated to practice (step 7).

Patient and public involvement

All end-users were represented during and involved in the development process of the programme by being a member of the working group. They were not involved in the development of the study design of dissemination of the findings.

Setting and participants

I-MANAGE is based on international evidence and tailored to the Dutch home care context. The programme was developed for community-dwelling older people who wish to remain living at home independently. The programme was developed in co-creation with stakeholders. Working groups were composed of a physiotherapist (PT), occupational therapist (OT), registered nurse (RN), domestic support worker (DSW), policy maker of the local municipality, psychologist, client representative,

For peer review only - http://bmjopen.bmj.com/site/about/guidelines.xhtml

informal caregiver representative, informal caregiver, reablement researcher, geriatrician, manager of community care teams, and geriatric rehabilitation expert.

Data collection

Development of a logic model (steps 1 to 6)

The logic model systematically visualises the aim and sub-aims of the programme, programme components, and intended results [32].

All steps were guided by literature research, especially steps 2 and 3 of the development process. To gain insight into current evidence-based practices (*step 2*), relevant scientific literature on reablement programmes was reviewed. The methodology and results of this literature research is described elsewhere [33]. To identify a theoretical foundation for the programme (*step 3*), a literature review was performed on the concept of disability and its underlying causal mechanisms [34].

Furthermore, a working group was composed and invited to three sessions (1.5 h each). Members of the working group included PTs, OTs, RNs, a DSW, a policymaker of the local municipality, a psychologist, a client representative, informal caregiver representatives, and a geriatric rehabilitation expert. The first session focused on the identification of the problem (*step 1*) and the examination of the current practice and context (*step 5*). Session two focused on previously identified evidence [33], which participants could complement with practices from their own work field (*step 2*), and the determination of needs (*step 4*), closely related to the previously identified problems. The third session focused on modelling the process and outcomes (*step 6*), during which a preliminary logic model was presented on which participants could provide feedback. Between sessions, working group members were consulted for additional input.

Additionally, we interviewed two informal caregivers, two PTs, a policy maker of the local municipality, a client and informal caregiver representative, and three geriatric rehabilitation experts. In total, 11 interviews were conducted, all focusing on identifying the problem (*step 1*), determining the needs (*step 4*), and gaining insight into current practice and context (*step 5*).

For peer review only - http://bmjopen.bmj.com/site/about/guidelines.xhtml

Lastly, observations were performed to examine current practice and context of home care services (*step 5*) and to identify problems (*step 1*). Six observations of half a day each were performed with a DSW, an RN, and a nursing assistant, and three days with allied health.

Translation to practice (step 7)

For each of the six programme components in the logic model, members of the working group were invited, as well as a geriatrician, reablement researcher, informal caregiver, and a manager of community care teams, to a session to translate components into practice (*step 7*). Based on the programme component, the most relevant stakeholders were invited. For example, on informal caregiver support, we invited an informal care consultant, an informal caregiver representative, and a psychologist; resulting in three to four members per working group. Each working group attended one session (1 h). Each session began with an introduction to the goal of the programme, the logic model, and the results of steps 1 to 6 related to the programme component. Afterwards, participants were asked open-ended questions about the practical implementation of the component, barriers, and facilitators. Lastly, results were summarised and participants were asked for final feedback. Table 1 provides an overview of the different methodologies used in each development step.

Table 1. Overview of the different methodologies used in each development step defined by Bleijenberg et al. [31]

Programme development steps	Methodology			
	Literature research	Observations	Individual interviews	Working groups
1. Problem identification	● (grey)	●	●	●
2. Identifying the evidence	●			●
3. Identifying or developing theory	●			
4. Determine the needs	● (grey)		●	●
5. Examining current practice and context	● (grey)	●	●	●
6. Modelling process and outcomes	● (grey)	● (grey)	● (grey)	●
7. Translation to practice	● (grey)			●

Note: The full black dots ● indicate a main source of information for the particular step, while the full grey dots ● (grey) indicate only a minor influence and guidance for the particular step.

Data analysis

For peer review only - http://bmjopen.bmj.com/site/about/guidelines.xhtml

We used data triangulation to verify the results. Our main source of information was the working group sessions. Sessions were recorded and transcribed non-verbatim. A thematic analysis was conducted. Common themes were identified within each step of the development process (e.g. lack of sufficient communication). Afterwards, the themes from all sessions were compared and searched for associations between, for example, identified problems and determined needs, or current scientific evidence and best practices of the organisation. The results of each session provided input and structure for the next session. Individual interviews were recorded and transcribed non-verbatim. Individual interviews, observations, and literature research were used to complement and check the information obtained through the working group sessions.

After each working group session or individual interview, a member check was conducted by summarising the most important findings. Between working group sessions, participants were contacted if clarification was needed. The results were discussed regularly within the research team.

For peer review only

For peer review only - http://bmjopen.bmj.com/site/about/guidelines.xhtml

RESULTS

Development of a logic model

The logic model starts with the aim and sub-aims of the programme based on identified problems and needs (*steps 1 and 4*), which are derived mostly from the working group sessions and individual interviews. To fulfil these aims, six programme components were determined: 1) improving assessment and goal-setting, 2) stimulating self-management during meaningful daily activities, 3) optimising the use of the physical environment, 4) optimising the use of the social environment, 5) improving interprofessional collaboration, and 6) engaging the informal caregiver. These originate from both the input from stakeholders (*steps 2, 4, 5 and 6)* and evidence-based practices from the literature (*step 2*). The intended results are reducing (I)ADL disability, improving self-management skills, increasing the quality of life of both the client and informal caregiver, and reducing healthcare utilisation and expenditures (proximal outcomes), which are common outcome measures in reablement programmes abroad, to remain living at home independently and avoid unnecessary transitions to institutional care (distal outcomes). Figure 1 presents the logic model. A detailed description of the results from the first five programme development steps is provided in Supplementary file 2.

[Insert Figure 1 here]

Translation to practice: description of the I-MANAGE programme

The I-MANAGE programme is the result of input from stakeholders during the last step of the development process (*step 7*) [31]. The following sections describe the programme in detail. The programme, as described, may serve as a blueprint for implementation in different local settings and leaves room for tailoring to the specific needs and resources of the organisation.

To achieve the intended results presented in the logic model, the programme applies a two-tiered approach: 1) focusing on the capabilities of the client and 2) paying attention to environmental factors (i.e. physical, social, and organisational). I-MANAGE is intended for community-dwelling older

For peer review only - http://bmjopen.bmj.com/site/about/guidelines.xhtml

people who are still able to (re)learn new skills and apply them in daily life without an indication of terminal illness or planned nursing home admission. The average duration of the programme is 8 weeks.

Interdisciplinary collaboration

I-MANAGE aims to improve interdisciplinary collaboration by facilitating intensive collaboration between the reablement team, client, informal caregiver, and other care professionals delivering care and support to the client and informal caregiver. The programme is delivered by an interdisciplinary reablement team, generally consisting of an RN, an OT, and, depending on the local context, supplemented with a PT, social worker, or DSW. The reablement team initiates the programme and is responsible for the direct support of the client and informal caregiver. A care coordinator is appointed, in most cases, the OT. Depending on the necessary care and goals set by the client, the reablement team may consult other care professionals (e.g. usual home care team, psychologist, or general practitioner), for example, during the intake phase for advice or while working on the client's goals. They are informed and coached by the reablement team.

Moreover, (bi-)weekly team meetings are organised to discuss the intake of new clients and informal caregivers, the progress made by clients, and final evaluations of clients' personal goals. Additionally, team meetings provide the opportunity to discuss specific cases with other team members, and to organise training. All members of the reablement team should have access to the client's electronic care file (ECF) to access the care plan, report progress, and follow up on each other's work. Lastly, when discharged from another care facility (e.g. hospital or rehabilitation facility), a smooth handover of client information is initiated so no necessary details of the client's care process are lost.

Practice-oriented training

For peer review only - http://bmjopen.bmj.com/site/about/guidelines.xhtml

Preceding the programme, the reablement team receives practice-oriented training focusing on adequately delivering I-MANAGE. Care professionals receive a detailed manual beforehand, consisting of background information and a description of the programme, including the goal, key components, and I-MANAGE's care process. Additionally, a toolkit for achieving individual client goals is provided (e.g. exercise booklet based on the OTAGO exercise programme [35], social map including welfare initiatives, and tools to assess needs and wishes of the caregiver). The training consists of multiple sessions. The first is a kick-off meeting for the reablement team, focusing on the rationale, goal, and content of I-MANAGE. Second, specific training sessions occur for OT and PT concerning the use of the Canadian Occupational Performance Measure (COPM) [36] to set personal goals with the client, and the OTAGO exercise booklet, respectively. Finally, the reablement team receives a booster session to practice and discuss challenging situations and focus on motivational and conversation skills. The kick-off and booster sessions each last two hours, and the specific sessions for OT and PT last one hour. Additionally, the programme stimulates training on the job, meaning that members of the reablement team can coach each other and external care professionals based on their own expertise.

I-MANAGE care process

The I-MANAGE care process consists of five consecutive phases: 1) initiation, 2) intake, 3) care plan, 4) care delivery, and 5) evaluation. Each phase is described in detail below and is presented in Figure 2.

Phase 1: Initiation

People are referred to the programme through community nurses, general practitioners, or institutional care facilities. Community-dwelling older adults eligible for I-MANAGE receive information about the programme. The care coordinator plans a first visit to present the programme to the client and their informal caregiver, and provides the opportunity to ask additional questions.

For peer review only - http://bmjopen.bmj.com/site/about/guidelines.xhtml

The care coordinator initiates an exploratory conversation based on the principles of positive health to gain insight into the client's needs and wishes [37].

Phase 2: Intake

Within the first week after the initiation phase, the OT performs an environmental assessment identifying necessary home modifications and assistive devices to ensure a safe environment. Additionally, the care coordinator or an assigned social worker performs an intake with the informal caregiver assessing their needs and wishes. Furthermore, the OT sets meaningful goals with the client using COPM [36] (maximum of five goals). Clients must score both their performance and their satisfaction with performing these activities [36]. Setting goals is guided by the exploratory conversation held in phase 1. The ultimate goal is to improve the client's participation and well-being; therefore, goals are not merely (I)ADL-related (e.g. meeting friends at the local café, or painting in the hobby room on the first floor). The goals are recorded in the client's ECF.

Phase 3: Care plan

Possible interventions and actions to achieve the client's goals and provide the informal caregiver with the right support are discussed within the reablement team. These interventions and actions are derived from the available toolkit. Afterwards, the care coordinator determines the final interventions and actions with the client and informal caregiver, guided by their preferences and capabilities, possibilities of the social network, and physical environment. Subsequently, the care plan is recorded in the client's ECF and shared with all members of the reablement team. The intensity of the programme depends on the care needs of the client and their pre-set goals, which may require a higher intensity in the beginning but less at the end when (sub-)goals are (partly) reached.

Phase 4: Care delivery

The reablement team delivers care and support, as described in the care plan. Care delivery is coordinated by the OT, who is also responsible for (re)learning and practicing meaningful daily

For peer review only - http://bmjopen.bmj.com/site/about/guidelines.xhtml

activities. The RN is responsible for supporting managing the client's personal care needs. The PT, when part of the reablement team, is responsible for functional training and stimulating participation in daily life. The client's environment is adapted (e.g. (home) modifications, assistive devices, and care technology) to support them in (re)learning activities. During the support with these meaningful activities, the client's self-management is stimulated by practicing these activities and ensuring that tasks are not taken over by care professionals or informal caregivers. If necessary, a social worker provides the informal caregiver with additional support decreasing their burden (e.g. respite care).

Phase 5: Evaluation

The evaluation of the care process is structurally embedded in interdisciplinary team meetings. Based on the conclusions drawn during these meetings, the care coordinator decides whether the care plan is continued, adjusted, or terminated. This is discussed with the client and informal caregiver. At the end of the programme, a formal evaluation of the client's goals takes place using COPM [36]. Afterwards, the care coordinator decides with the client and informal caregiver if the programme should be extended (max. two weeks) to ensure all goals are sufficiently reached or if the client needs referral to usual care.

[Insert Figure 2 here]

For peer review only - http://bmjopen.bmj.com/site/about/guidelines.xhtml

DISCUSSION

This manuscript describes the development, rationale, and content of I-MANAGE, a community-based reablement programme. The programme was developed for community-dwelling older adults to improve their self-management and participation in daily life and ensure that they can remain living at home independently as long as possible and avoid unnecessary transitions to institutional care, while also increasing quality of life and decreasing the burden of informal caregivers.

The described programme serves as a blueprint and leaves room to tailor the intervention to a specific context and the needs of the organisation. For example, the programme leaves room to the organisation to choose which target group would benefit the most. Additionally, delivering the programme by an interdisciplinary team is a prerequisite; however, depending on the available resources, the organisation can decide on the composition of the reablement team. Moreover, I-MANAGE is a multi-component programme in which organisations can integrate their own innovative practices, especially when working towards and reaching clients' meaningful goals. Moe and Brinchmann [38] confirmed the necessity of tailoring reablement services to local conditions by arguing that establishing reablement in an existing organisational structure is a complex process.

The programme contains several key elements that are considered essential. First, we implemented a standardised goal-setting tool, COPM [36], preceded by an extensive intake based on the principles of positive health [37]. Previous research has indicated that using standardised assessment or goal-setting tools could increase the effectiveness of reablement interventions [27]. Additionally, it increased client involvement and helps professionals to identify meaningful activities with the client [39]. Second, interdisciplinary collaboration is important in I-MANAGE (e.g. appointing a care coordinator, scheduling (bi-)weekly meetings, and a shared ECF). Recent literature indicates that investing in interdisciplinary collaboration stimulates patient-centred care by ensuring a holistic view of the client's situation and creates shared responsibility [40-42]. This is achieved by good communication and coordination within the team but also with the client and informal caregiver [43]. It also stimulates continuous learning and is experienced as exciting and constructive by care

For peer review only - http://bmjopen.bmj.com/site/about/guidelines.xhtml

professionals [42, 43]. Moreover, delivery of the programme by an interdisciplinary team, including allied health, such as OTs, is deemed valuable because of their educational background [27, 44]. Third, supporting informal caregivers also contributes to the success of I-MANAGE. Previous research found that providing informal caregivers with the right psychosocial and educational support strengthens their ability to cope with their new role [45, 46]. Fourth, I-MANAGE promotes optimal use of the social and physical environment, which is essential since a demanding environment can either stimulate or hinder a person's participation in meaningful activities [47]. Fifth, the programme stimulates self-management through participation in meaningful daily activities, which is a core element within reablement ('doing with…' rather than 'doing for…' the client) [19, 48].

When implementing the programme, we give high priority to maintaining fidelity by using several implementation strategies [49]. A first strategy is practice-oriented training. Over the course of the implementation of the programme, we conduct ongoing educational and training meetings together with training on the job, where care professionals have the chance to learn from other disciplines. Additionally, multiple advisory boards and working groups are set up to discuss the implementation process and adjust where needed. We also invest in getting the management of care organisations on board to support the implementation within their organisation. Moreover, knowledge exchange between sites in the Netherlands where reablement is being implemented is set up to share experiences and best practices. Investing in the self-efficacy of care professionals and a suitable organisational structure is essential because it remains a challenge to change behaviour and existing organisational structures successfully [38, 50, 51].

A strength of I-MANAGE and its development is that it is theory- and evidence-based [33, 34], which has proven to be advantageous when developing effective interventions [52, 53]. Moreover, Thuesen et al. [54] highlight the demand for making theory explicit in reablement interventions, and addressing the physical, psychological, and sociocultural perspectives of ageing within these interventions. Another strength of the development process was the co-creation process, giving a voice to multiple stakeholders and making the programme suitable for practice. However, there are

For peer review only - http://bmjopen.bmj.com/site/about/guidelines.xhtml

also some limitations related to this co-creation process. First, during the process, we always informed management to obtain their support, but they were not included in the working groups, which could have been an important opinion lacking. Second, we aimed to include all end users of the programme during the development process (care professionals, clients, and informal caregivers). However, we only included client and informal caregiver representatives in the working groups and interviewed only two informal caregivers between sessions. We also obtained data from many different sources (i.e. scientific literature, individual interviews, working groups, and observations), which made it difficult to find common ground throughout all the sources and forced us to compromise. However, this use of data triangulation is also a strength of the development of the programme because we were able to check our findings multiple times with different sources, increasing the validity of the results.

During the co-creation process, some influencing factors for implementation were identified, for example, available resources in terms of staff and finances, and different IT systems, making communication and information transfer less evident. Moe and Brinchmann [38] mentioned that, next to communication and IT systems, habitual ways of offering health services and lacking knowledge about the rehabilitation potential of older people and benefits of active ageing are also experienced as barriers. A recent scoping review identified several factors that act as barriers and facilitators during the implementation of care innovations, among which available resources and communication were listed as possible barriers to implementation [55, 56]. It is critical to consider these factors when implementing I-MANAGE in practice. Regarding future research, (cost-)effectiveness has not yet been proven, but is currently being implemented and evaluated at different Dutch care providers. The systematic reporting of the development of the programme provides useful insight for future research looking to develop complex health interventions or to implement a similar programme.

For peer review only

FIGURE LEGENDS

Figure 1: Logic model of the I-MANAGE programme. The logic model of the I-MANAGE programme presents the aim and associated sub-aims of the programme, the programme components, and proximal and distal outcomes.

Figure 2: Detailed presentation of the I-MANAGE care process. I-MANAGE has a 5-phase care process (initiation, intake, care plan, care delivery, and evaluation), preceded with the referral of the client through different routes. After an average duration of 8 weeks, clients are referred to aftercare. Interdisciplinary collaboration is a continuous element of the programme.

ETHICS APPROVAL AND CONSENT TO PARTICIPATE

As participants were not being subjected to actions and no rules of behaviour were imposed on them no ethical approval was needed according to the Dutch 'Medical Research Involving Human Subjects Act' (WMO). Nevertheless the study was carried out in accordance with the principles of ethical research and all participants involved in the working group sessions and interviews provided either written or verbal informed consent prior to the start of each session.

AVAILABILITY OF DATA AND MATERIALS

The datasets used and/or analysed during the current study available from the corresponding author on reasonable request.

COMPETING INTEREST

The authors declare that they have no competing interests

For peer review only - http://bmjopen.bmj.com/site/about/guidelines.xhtml

FUNDING

This work was supported by the European Union's Horizon 2020 research and innovation programme under the Marie Skłodowska-Curie grant agreement No 812656 as part of the TRANS-SENIOR project (www.trans-senior.eu).

AUTHORS' CONTRIBUTORS

All authors – IM, HV, GIJMK, JCMvH, EV, GG, SFM – were involved in the development of the I-MANAGE programme. IM wrote the first draft of the article. All authors reviewed the manuscript and approved the final version.

ACKNOWLEDGEMENTS

We would like to thank the Dutch stakeholders who have participated in the working group sessions, observations, and individual interviews, and therefore have helped with the development of a reablement programme suitable for the Dutch home care setting: Angelica de Lang, Nelly Jacobs, Jose Blezer, Belkis Bozdemir, Marcel Mannens, Marc Lowis, Maud Scholtes, Mandy Boosten, Hella Senden, Angèle van Kasteren, Anne Gulpers, Eveline van Velthuijsen, Anouschka Feenstra, Elfi Pakbeers, Nadine Klinkers, Marjo Gartener (MeanderGroep Zuid-Limburg), Ewout Smit (Vivium Zorggroep), Teuni Rooijackers, Angelique de Rijk (Maastricht University), Bianca Miltenburg (Cicero Zorggroep), Marie-Therese Bindels, Femke Scheuerman (gemeente Kerkrade), Marja Veenstra (Burgerkracht Limburg).

SUPPLEMENTAL MATERIAL

Supplementary file 1: GUIDED checklist. Completed guideline for reporting for intervention development studies.

Supplementary file 2: Overview of the intermediate results of the first five steps of the development phase.

For peer review only

REFERENCES

1. Everink IHJ, van Haastregt JCM, Kempen GIJM, et al. Uitdagingen in de geriatrische revalidatiezorg: de ontwikkeling van een zorgpad. Tijdschr Gerontol Geriatr. 2015 2015/04/01;46(2):104-12.

2. Storm M, Siemsen IMD, Laugaland K, et al. Quality in transitional care of the elderly: Key challenges and relevant improvement measures. Int J Integr Care. 2014;14:e013.

3. Brown GC. Living too long: the current focus of medical research on increasing the quantity, rather than the quality, of life is damaging our health and harming the economy. EMBO Rep. 2015;16(2):137-41.

4. Rustad EC, Furnes B, Cronfalk BS, et al. Older patients' experiences during care transition. Patient Prefer Adherence. 2016;10:769-79.

5. Wahl HW, Fange A, Oswald F, et al. The home environment and disability-related outcomes in aging individuals: What is the empirical evidence? Gerontologist. 2009 Jun;49(3):355-67.

6. Arnau A, Espaulella J, Serrarols M, et al. Risk factors for functional decline in a population aged 75 years and older without total dependence: A one-year follow-up. Arch Gerontol Geriatr. 2016;65:239-47.

7. Tappenden P, Campbell F, Rawdin A, et al. The clinical effectiveness and cost-effectiveness of home-based, nurse-led health promotion for older people: A systematic review. Health Technol Assess. 2012;16(20):1-72.

8. Fujisawa R, Colombo F. The Long-Term Care Workforce: Overview and Strategies to Adapt Supply to a Growing Demand. 2009.

9. Arbaje AI, Kansagara DL, Salanitro AH, et al. Regardless of age: Incorporating principles from geriatric medicine to improve care transitions for patients with complex needs. J Gen Intern Med. 2014;29(6):932-9.

10. Koopmans RT, Lavrijsen JC, Hoek JF, et al. Dutch elderly care physician: a new generation of nursing home physician specialists. J Am Geriatr Soc. 2010;58(9):1807-9.

For peer review only - http://bmjopen.bmj.com/site/about/guidelines.xhtml

11. Nicholson C, Meyer J, Flatley M, et al. The experience of living at home with frailty in old age: A psychosocial qualitative study. Int J Nurs Stud. 2013 2013/09/01/;50(9):1172-9.

12. Aspinal F, Glasby J, Rostgaard T, et al. New horizons: Reablement - supporting older people towards independence. Age Ageing. 2016;45(5):572-6.

13. Beard JR, Officer A, de Carvalho IA, et al. The World report on ageing and health: a policy framework for healthy ageing. The Lancet. 2016;387(10033):2145-54.

14. Ferrah N, Ibrahim JE, Kipsaina C, et al. Death Following Recent Admission Into Nursing Home From Community Living: A Systematic Review Into the Transition Process. J Aging Health. 2018;30(4):584-604.

15. Oliver D, Foot C, Humphries R. Making our health and care systems fit for an ageing population: King's Fund London: UK; 2014.

16. Wiles JL, Leibing A, Guberman N, et al. The meaning of "aging in place" to older people. Gerontologist. 2012 Jun;52(3):357-66.

17. Cesari M, Vellas B, Hsu F-C, et al. A physical activity intervention to treat the frailty syndrome in older persons-results from the LIFE-P study. J Gerontol A Biol Sci Med Sci. 2015;70(2):216-22.

18. Daniels R, Metzelthin SF, van Rossum E, et al. Interventions to prevent disability in frail c ommunity-dwelling older persons: An overview. Eur J Ageing. 2010;7(1):37-55.

19. Metzelthin SF, Rostgaard T, Parsons M, et al. Development of an internationally accepted definition of reablement: a Delphi study. Ageing and Society. 2020:1-16.

20. Doh D, Smith R, Gevers P. Reviewing the reablement approach to caring for older people. Ageing and Society. 2019:1-13.

21. Metzelthin SF, Zijlstra GA, van Rossum E, et al. 'Doing with ...' rather than 'doing for ...' older adults: rationale and content of the 'Stay Active at Home' programme. Clin Rehabil. 2017;31(11):1419-30.

For peer review only

For peer review only - http://bmjopen.bmj.com/site/about/guidelines.xhtml

22. Tessier A, Beaulieu M-D, McGinn CA, et al. Effectiveness of Reablement: A Systematic Review. Healthcare policy. 2016;11(4):49-59.

23. Whitehead PJ, Worthington EJ, Parry RH, et al. Interventions to reduce dependency in personal activities of daily living in community dwelling adults who use homecare services: A systematic review. Clin Rehabil. 2015 Nov;29(11):1064-76.

24. Resnick B, Galik E, Boltz M. Function focused care approaches: Literature review of progress and future possibilities. J Am Med Dir Assoc. 2013 2013/05/01/;14(5):313-8.

25. Mjøsund HL, Moe CF, Burton E, et al. Integration of Physical Activity in Reablement for Community Dwelling Older Adults: A Systematic Scoping Review. J Multidiscip Healthc. 2020;13:1291-315.

26. Duncan E, Cathain A, Rousseau N, et al. Guidance for reporting intervention development studies in health research (GUIDED): an evidence-based consensus study. BMJ Open. 2020;10(4):e033516.

27. Buma LE, Vluggen S, Zwakhalen S, et al. Effects on clients' daily functioning and common features of reablement interventions: a systematic literature review. Eur J Ageing. 2022 2022/05/03.

28. Hoffmann TC, Glasziou PP, Boutron I, et al. Better reporting of interventions: template for intervention description and replication (TIDieR) checklist and guide. BMJ : British Medical Journal. 2014;348:g1687.

29. Craig P, Dieppe P, Macintyre S, et al. Developing and evaluating complex interventions: the new Medical Research Council guidance. BMJ. 2008;337:a1655.

30. Skivington K, Matthews L, Simpson SA, et al. A new framework for developing and evaluating complex interventions: update of Medical Research Council guidance. BMJ. 2021;374:n2061.

31. Bleijenberg N, de Man-van Ginkel JM, Trappenburg JCA, et al. Increasing value and reducing waste by optimizing the development of complex interventions: Enriching the development phase of the Medical Research Council (MRC) Framework. Int J Nurs Stud. 2018;79:86-93.

For peer review only - http://bmjopen.bmj.com/site/about/guidelines.xhtml

32. W.K. Kellogg Foundation. Logic Model Development Guide. Michigan2004. Available from: https://www.wkkf.org/resource-directory/resource/2006/02/wk-kellogg-foundation-logic-model-development-guide.

33. Mouchaers I, Van Haastregt JCM, Verbeek H, et al. « Reablement » comme outil thérapeutique pour offrir aux personnes âgées une vie de qualité à domicile après une réadaptation gériatrique. Rev Med Brux. 2021;42(4):324-32.

34. Mouchaers I, Verbeek H, Kempen GIJM, et al. The concept of disability and its causal mechanisms in older people over time from a theoretical perspective: a literature review. Eur J Ageing. 2022 2022/01/29.

35. Campbell AJ, Robertson MC. Het Otago Oefenprogramma om valincidenten bij ouderen te voorkomen. Amersfoort: Nederlandse Vereniging voor Fysiotherapie in de Geriatrie; 2013.

36. Law M, Baptiste S, McColl M, et al. The Canadian Occupational Performance Measure: An Outcome Measure for Occupational Therapy. Canadian Journal of Occupational Therapy. 1990;57(2):82-7.

37. Huber M, van Vliet M, Giezenberg M, et al. Towards a 'patient-centred' operationalisation of the new dynamic concept of health: a mixed methods study. BMJ Open. 2016;6(1):e010091.

38. Moe C, Brinchmann BS. Tailoring reablement: A grounded theory study of establishing reablement in a community setting in Norway. Health Soc Care Community. 2018 Jan;26(1):113-21.

39. Rose A, Rosewilliam S, Soundy A. Shared decision making within goal setting in rehabilitation settings: A systematic review. Patient Educ Couns. 2017 Jan;100(1):65-75.

40. Schmutz JB, Meier LL, Manser T. How effective is teamwork really? The relationship between teamwork and performance in healthcare teams: a systematic review and meta-analysis. BMJ Open. 2019;9(9):e028280.

For peer review only

41. Ebrahimi Z, Patel H, Wijk H, et al. A systematic review on implementation of person-centered care interventions for older people in out-of-hospital settings. Geriatric Nursing. 2021 2021/01/01/;42(1):213-24.

42. Eliassen M, Moholt JM. Boundary work in task-shifting practices - a qualitative study of reablement teams. Physiother Theory Pract. 2022 Apr 18:1-14.

43. Birkeland A, Tuntland H, Førland O, et al. Interdisciplinary collaboration in reablement - a qualitative study. J Multidiscip Healthc. 2017;10:195-203.

44. Reeves S, Fletcher S, Barr H, et al. A BEME systematic review of the effects of interprofessional education: BEME Guide No. 39. Med Teach. 2016 Jul;38(7):656-68.

45. Lopez-Hartmann M, Wens J, Verhoeven V, et al. The effect of caregiver support interventions for informal caregivers of community-dwelling frail elderly: a systematic review. Int J Integr Care. 2012 Jul-Sep;12:e133.

46. Spiers GF, Liddle J, Kunonga TP, et al. What are the consequences of caring for older people and what interventions are effective for supporting unpaid carers? A rapid review of systematic reviews. BMJ Open. 2021;11(9):e046187.

47. de Vries NM, Staal JB, van Ravensberg CD, et al. Outcome instruments to measure frailty: A systematic review. Ageing Res Rev. 2011 2011/01/01/;10(1):104-14.

48. Clotworthy A, Kusumastuti S, Westendorp RGJ. Reablement through time and space: a scoping review of how the concept of 'reablement' for older people has been defined and operationalised. BMC Geriatr. 2021 2021/01/15;21(1):61.

49. Powell BJ, Waltz TJ, Chinman MJ, et al. A refined compilation of implementation strategies: results from the Expert Recommendations for Implementing Change (ERIC) project. Implementation Science. 2015 2015/02/12;10(1):21.

50. Reay T, Goodrick E, D'Aunno T. Health Care Research and Organization Theory. Cambridge: Cambridge University Press; 2021. Available from:

For peer review only - http://bmjopen.bmj.com/site/about/guidelines.xhtml

https://www.cambridge.org/core/elements/health-care-research-and-organization-theory/18F375C5D5DEBE92B725FAB547EA076D.

51. Rooijackers T, van Rossum E, Zijlstra GAR, et al. Effectiveness of a reablement training program on self-efficacy and outcome expectations regarding client activation in homecare staff: A cluster randomized controlled trial. Geriatric Nursing. 2022 2022/01/01/;43:104-12.

52. Michie S, Prestwich A. Are interventions theory-based? Development of a theory coding scheme. Health Psychol. 2010 Jan;29(1):1-8.

53. Bartholomew LK, Mullen PD. Five roles for using theory and evidence in the design and testing of behavior change interventions. J Public Health Dent. 2011;71(s1):S20-S33.

54. Thuesen J, Feiring M, Doh D, et al. Reablement in need of theories of ageing: would theories of successful ageing do? Ageing and Society. 2021:1-13.

55. Fakha A, Groenvynck L, de Boer B, et al. A myriad of factors influencing the implementation of transitional care innovations: a scoping review. Implementation Science. 2021 2021/02/26;16(1):21.

56. Waltz TJ, Powell BJ, Matthieu MM, et al. Use of concept mapping to characterize relationships among implementation strategies and assess their feasibility and importance: results from the Expert Recommendations for Implementing Change (ERIC) study. Implementation Science. 2015 2015/08/07;10(1):109.

For peer review only

For peer review only - http://bmjopen.bmj.com/site/about/guidelines.xhtml

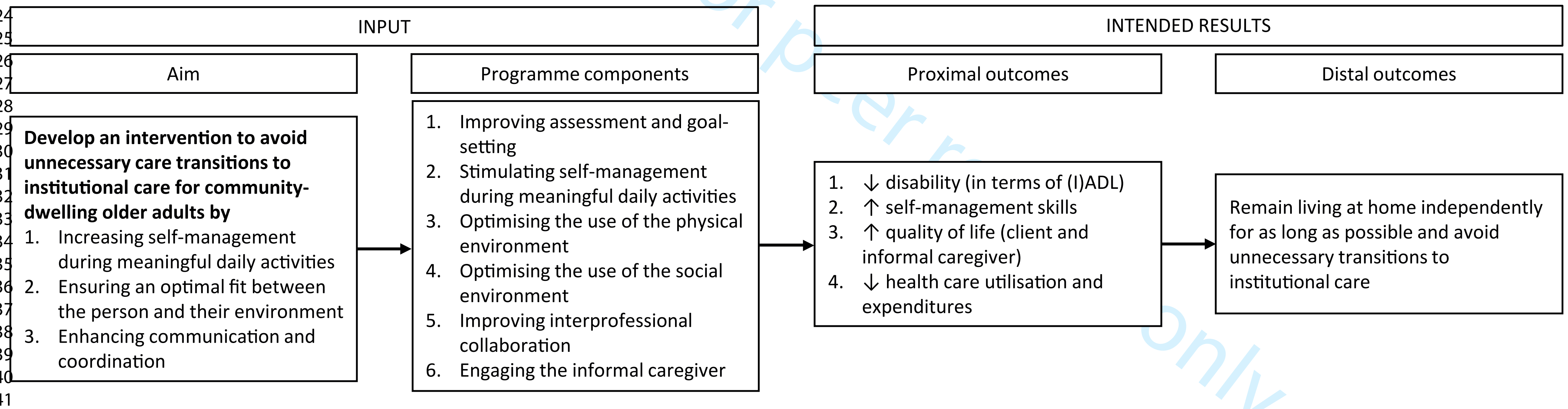

For peer review only

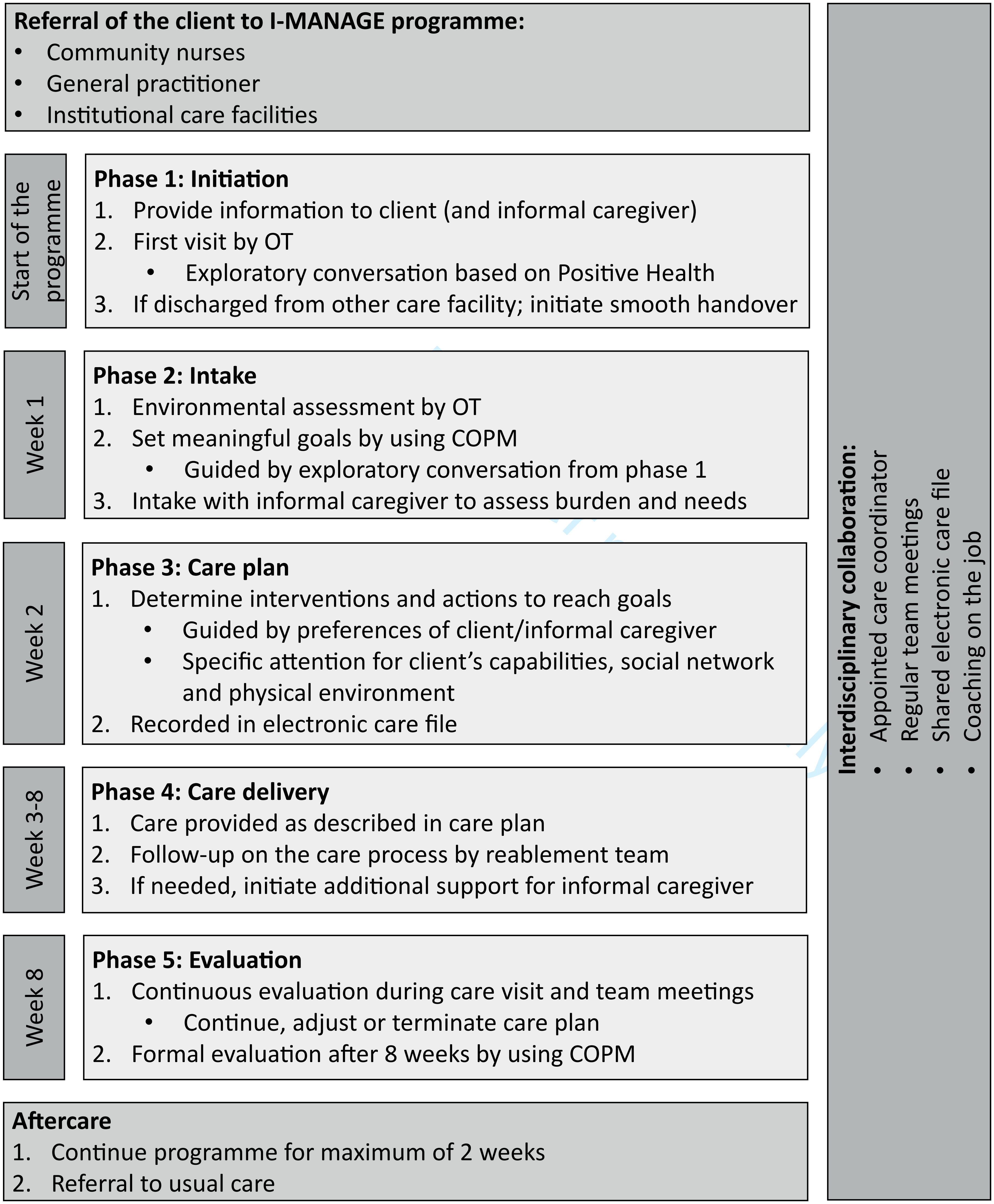
Referral of the client to I-MANAGE programme:
• Community nurses
• General practitioner
• Institutional care facilities
Start of the programme
Phase 1: Initiation
1. Provide information to client (and informal caregiver)
2. First visit by OT
• Exploratory conversation based on Positive Health
3. If discharged from other care facility; initiate smooth handover
Week 1
Phase 2: Intake
1. Environmental assessment by OT
2. Set meaningful goals by using COPM
• Guided by exploratory conversation from phase 1
3. Intake with informal caregiver to assess burden and needs
Week 2
Phase 3: Care plan
1. Determine interventions and actions to reach goals
• Guided by preferences of client/informal caregiver
• Specific attention for client's capabilities, social network and physical environment
2. Recorded in electronic care file
Week 3-8
Phase 4: Care delivery
1. Care provided as described in care plan
2. Follow-up on the care process by reablement team
3. If needed, initiate additional support for informal caregiver
Week 8
Phase 5: Evaluation
1. Continuous evaluation during care visit and team meetings
• Continue, adjust or terminate care plan
2. Formal evaluation after 8 weeks by using COPM
Aftercare
1. Continue programme for maximum of 2 weeks
2. Referral to usual care
Interdisciplinary collaboration:
• Appointed care coordinator
• Regular team meetings
• Shared electronic care file
• Coaching on the job

Supplementary file 1: GUIDED checklist. A guideline for reporting for intervention development studies.

**Item description**	**Explanation**	**Page in manuscript where item is located**	**Other***
Report the context for which the intervention was developed.	Understanding the context in which an intervention was developed informs readers about the suitability and transferability of the intervention to the context in which they are considering evaluating, adapting or using the intervention. Context here can include place, organisational and wider socio- political factors that may influence the development and/or delivery of the intervention.	p5	
Report the purpose of the intervention development process.	Clearly describing the purpose of the intervention specifies what it sets out to achieve. The purpose may be informed by research priorities, for example those identified in systematic reviews, evidence gaps set out in practice guidance such as The National Institute for Health and Care Excellence or specific prioritisation exercises such as those undertaken with patients and practitioners through the James Lind Alliance.	p3-4	
Report the target population for the intervention development process.	The target population is the population that will potentially benefit from the intervention – this may include patients, clinicians, and/or members of the public. If the target population is clearly described then readers will be able to understand the relevance of the intervention to their own research or practice. Health inequalities, gender and ethnicity are features of the target population that may be relevant to intervention development processes.	p10	
Report how any published intervention development approach contributed to the development process.	Many formal intervention development approaches exist and are used to guide the intervention development process (e.g. 6Squid or The Person Based Approach to Intervention Development). Where a formal intervention development approach is used, it is helpful to describe the process that was followed, including any deviations. More general approaches to intervention development also exist and have been categorised as follows: Target Population-centred intervention development; evidence and theory-based intervention development; partnership intervention development; implementation-based intervention development; efficacy- based intervention development; step or phased-based intervention development; and intervention-specific intervention development. These approaches do not always have specific guidance that describe their use. Nevertheless, it is helpful to give a rich description of how any published approach was operationalised	p5	

For peer review only

Report how evidence from different sources informed the intervention development process.	Intervention development is often based on published evidence and/or primary data that has been collected to inform the intervention development process. It is useful to describe and reference all forms of evidence and data that have informed the development of the intervention because evidence bases can change rapidly, and to explain the manner in which the evidence and/or data was used. Understanding what evidence was and was not available at the time of intervention development can help readers to assess transferability to their current situation.	p6	Supplementary file B + [36]
Report how/if published theory informed the intervention development process.	Reporting whether and how theory informed the intervention development process aids the reader's understanding of the theoretical rationale that underpins the intervention. Though not mentioned in the e-Delphi or consensus meeting, it became increasingly apparent through the development of our guidance that this theory item could relate to either existing published theory or programme theory.	p6	Supplementary file B + [37]
Report any use of components from existing interventions in the current intervention development process.	Some interventions are developed with components that have been adopted from existing interventions. Clearly identifying components that have been adopted or adapted and acknowledging their original source helps the reader to understand and distinguish between the novel and adopted components of the new intervention.	p6 + p9	Supplementary file B + [36]
Report any guiding principles, people or factors that were prioritised when making decisions during the intervention development process.	Reporting any guiding principles that governed the development of the application helps the reader to understand the authors' reasoning behind the decisions that were made. These could include the examples of particular populations who views are being considered when designing the intervention, the modality that is viewed as being most appropriate, design features considered important for the target population, or the potential for the intervention to be scaled up.	p5-7	Supplementary file B
Report how stakeholders contributed to the intervention development process.	Potential stakeholders can include patient and community representatives, local and national policy makers, health care providers and those paying for or commissioning health care. Each of these groups may influence the intervention development process in different ways. Specifying how differing groups of stakeholders contributed to the intervention development process helps the reader to understand how stakeholders were involved and the degree of influence they had on the overall process. Further detail on how to integrate stakeholder contributions within intervention reporting are available.	p5-7	
Report how the intervention	Intervention development is frequently an iterative process. The conclusion of the initial	p15-16 /	

For peer review only

changed in content and format from the start of the intervention development process.	phase of intervention development does not necessarily mean that all uncertainties have been addressed. It is helpful to list remaining uncertainties such as the intervention intensity, mode of delivery, materials, procedures, or type of location that the intervention is most suitable for. This can guide other researchers to potential future areas of research and practitioners about uncertainties relevant to their healthcare context.	p9 vs. p12	
Report any changes to the intervention required or likely to be required for subgroups.	Specifying any changes that the intervention development team perceive are required for the intervention to be delivered or tailored to specific sub groups enables readers to understand the applicability of the intervention to their target population or context. These changes could include changes to personnel delivering the intervention, to the content of the intervention, or to the mode of delivery of the intervention.	p15-16	
Report important uncertainties at the end of the development process.	Intervention development is frequently an iterative process. The conclusion of the initial phase of intervention development does not necessarily mean that all uncertainties have been addressed. It is helpful to list remaining uncertainties such as the intervention intensity, mode of delivery, materials, procedures, or type of location that the intervention is most suitable for. This can guide other researchers to potential future areas of research and practitioners about uncertainties relevant to their healthcare context.	p15-16	
Follow TIDieR guidance when describing the developed intervention.	Interventions have been poorly reported for a number of years. In response to this, internationally recognized guidance has been published to support the high quality reporting of health care interventions and public health interventions. This guidance should therefore be followed when describing a developed intervention.		
Brief name.	Provide the name or a phrase that describes the intervention.	p4	
Rationale, theory and aim of the elements essential to the intervention.	Describe any rationale, theory, or goal of the elements essential to the intervention.	p6	Supplementary file B + [37]
Materials used in the intervention delivery or in the training of intervention providers.	Describe any physical or informational materials used in the intervention, including those provided to participants or used in intervention delivery or in training of intervention providers. Provide information on where the materials can be accessed (e.g. online appendix, URL).	p10-13	
Procedures, activities and/or processes used including enabling	Describe each of the procedures, activities, and/or processes used in the intervention, including any enabling or support activities.	p10-13	

For peer review only

or support activities.			
Expertise and background of intervention providers.	For each category of intervention provider (e.g. psychologist, nursing assistant), describe their expertise, background and any specific training given.	p10-11	
How the intervention was provided.	Describe the modes of delivery (e.g. face-to-face or by some other mechanism, such as internet or telephone) of the intervention and whether it was provided individually or in a group.	p10-13	
Location where the intervention occurred (incl. necessary infrastructure or relevant features).	Describe the type(s) of location(s) where the intervention occurred, including any necessary infrastructure or relevant features.	p5	
When and how much the intervention was delivered.	Describe the number of times the intervention was delivered and over what period of time including the number of sessions, their schedule, and their duration, intensity or dose.	p10 + 13	
Tailoring – what, why, when and how.	If the intervention was planned to be personalised, titrated or adapted, then describe what, why, when, and how.	p12-13	
Modifications. †	If the intervention was modified during the course of the study, describe the changes (what, why, when, and how).	N/A	
Strategies to improve or maintain fidelity.	If intervention adherence or fidelity was assessed, describe how and by whom, and if any strategies were used to maintain or improve fidelity, describe them.	p16	
Adherence and fidelity.†	If intervention adherence or fidelity was assessed, describe the extent to which the intervention was delivered as planned.	N/A	
Report the intervention development process in an open access format.	Unless reports of intervention development are available people considering using an intervention cannot understand the process that was undertaken and make a judgement about its appropriateness to their context. It also limits cumulative learning about intervention development methodology and observed consequences at later evaluation, translation and implementation stages. Reporting intervention development in an open access (Gold or Green) publishing format increases the accessibility and visibility of intervention development research and makes it more likely to be read and used. Potential platforms for open access publication of intervention development include open access journal publications, freely accessible funder reports or a study web-page that details the intervention development process.		Aim of this manuscript

Note. * e.g. if item is reported elsewhere, then the location of this information can be stated here. † If completing the TIDieR checklist for a protocol, these items are not relevant to the protocol and cannot be described until the study is complete.

For peer review only

For peer review only

Supplementary file 2. Overview of the intermediate results of the first five steps of the development phase.

Programme development step	Results
1. Problem identification	The identified problems were situated in three main areas:<br>1. A lack of attention to meaningful daily activities. According to the working group, older persons were mainly focused on functional goals related to (I)ADL tasks rather than meaningful daily activities that increase their well-being. Additionally, the focus of care is often on eliminating diseases and symptoms instead of maintaining quality of life [11, 12];<br>2. There appeared a gap between the person's abilities, needs, and wishes, and the environment they reside in. When older people experience a deterioration in health status, their environment is often not adapted, i.e. the necessary home modifications and assistive devices are not always in place [4, 5];<br>3. Communication and coordination within the care trajectory of an older person. There is insufficient communication amongst different care professionals but also with older persons and informal caregivers [1, 4].
2. Identifying the evidence	The identification of the evidence-based practices was mainly based on literature research. A literature review was performed to gain insight into current evidence on effective reablement programmes aimed at improving the independent living of older persons [33]. We specifically sought to gain a general understanding of different reablement programmes, their characteristics (e.g. duration), content, outcome measures, and effectiveness. Thirteen programmes were identified, all intended for (I)ADL-impaired community-dwelling older adults. The most common features were: 1) delivery by a trained and coordinated multidisciplinary team and 2) implementation of an intake assessment and goal-setting tool. The applied interventions focused on supporting (I)ADL and physical functioning, and improving home safety. Nine programmes showed a statistically significant improvement on at least one of the outcome measures (e.g. (I)ADL functioning).
3. Identifying or developing theory	A literature review was performed on the concept of disability and its working mechanisms [34] to identify a solid theoretical foundation for our programme (*step 3*). We concluded that by optimising the use of personal, as well as environmental, resources and focusing on health and functioning, rather than disability, an older person's independence and wellbeing could be improved, especially while performing meaningful daily activities in accordance with the person's needs and preferences.
4. Determine the needs	The determined needs are closely related to the identified problems. Stakeholders expressed the need for:<br>1. Standardised goal-setting at the beginning of the care process. Goals should be meaningful, embedded in the older person's own environment, and not solely focused on (I)ADL;<br>2. Timely assessment and, if necessary, adaptations to the home environment of the older person. This concerns adaptations to the built environment, as well as the availability of assistive devices. Additionally, the social environment of the older person needs to be assessed in order to make optimal use of it or provide additional support if the social network is rather weak;<br>3. Improved communication and coordination within the care trajectory of the older person. For example, a smooth handover of information between care professionals and organisations, involvement of the informal caregiver when discussing a client's care needs, multidisciplinary collaboration with clear roles for each provider, and the appointment of a care coordinator.
5. Examining current practice and context	When examining the current practice and context we focused on positive aspects and possible barriers for implementation. Possible barriers could be different financial arrangements and different communication systems within a multidisciplinary team. Positive aspects include the comprehensive assessment by community nurses at the start

For peer review only

	of receiving community care, a signalling role for domestic support workers, and the presence of various welfare initiatives in the Netherlands. A suggestion was made to include the principles of Positive Health [37] in the programme, a best practice that is already known and applied by some care professionals.

For peer review only

Supplementary file 1: GUIDED checklist. A guideline for reporting for intervention development studies.

Item description	Explanation	Page in manuscript where item is located	Other*
Report the context for which the intervention was developed.	Understanding the context in which an intervention was developed informs readers about the suitability and transferability of the intervention to the context in which they are considering evaluating, adapting or using the intervention. Context here can include place, organisational and wider socio- political factors that may influence the development and/or delivery of the intervention.	p5	
Report the purpose of the intervention development process.	Clearly describing the purpose of the intervention specifies what it sets out to achieve. The purpose may be informed by research priorities, for example those identified in systematic reviews, evidence gaps set out in practice guidance such as The National Institute for Health and Care Excellence or specific prioritisation exercises such as those undertaken with patients and practitioners through the James Lind Alliance.	p3-4	
Report the target population for the intervention development process.	The target population is the population that will potentially benefit from the intervention – this may include patients, clinicians, and/or members of the public. If the target population is clearly described then readers will be able to understand the relevance of the intervention to their own research or practice. Health inequalities, gender and ethnicity are features of the target population that may be relevant to intervention development processes.	p10	
Report how any published intervention development approach contributed to the development process.	Many formal intervention development approaches exist and are used to guide the intervention development process (e.g. 6Squid or The Person Based Approach to Intervention Development). Where a formal intervention development approach is used, it is helpful to describe the process that was followed, including any deviations. More general approaches to intervention development also exist and have been categorised as follows: Target Population-centred intervention development; evidence and theory-based intervention development; partnership intervention development; implementation-based intervention development; efficacy- based intervention development; step or phased-based intervention development; and intervention-specific intervention development. These approaches do not always have specific guidance that describe their use. Nevertheless, it is helpful to give a rich description of how any published approach was operationalised	p5	

For peer review only

Report how evidence from different sources informed the intervention development process.	Intervention development is often based on published evidence and/or primary data that has been collected to inform the intervention development process. It is useful to describe and reference all forms of evidence and data that have informed the development of the intervention because evidence bases can change rapidly, and to explain the manner in which the evidence and/or data was used. Understanding what evidence was and was not available at the time of intervention development can help readers to assess transferability to their current situation.	p6	Supplementary file B + [36]
Report how/if published theory informed the intervention development process.	Reporting whether and how theory informed the intervention development process aids the reader's understanding of the theoretical rationale that underpins the intervention. Though not mentioned in the e-Delphi or consensus meeting, it became increasingly apparent through the development of our guidance that this theory item could relate to either existing published theory or programme theory.	p6	Supplementary file B + [37]
Report any use of components from existing interventions in the current intervention development process.	Some interventions are developed with components that have been adopted from existing interventions. Clearly identifying components that have been adopted or adapted and acknowledging their original source helps the reader to understand and distinguish between the novel and adopted components of the new intervention.	p6 + p9	Supplementary file B + [36]
Report any guiding principles, people or factors that were prioritised when making decisions during the intervention development process.	Reporting any guiding principles that governed the development of the application helps the reader to understand the authors' reasoning behind the decisions that were made. These could include the examples of particular populations who views are being considered when designing the intervention, the modality that is viewed as being most appropriate, design features considered important for the target population, or the potential for the intervention to be scaled up.	p5-7	Supplementary file B
Report how stakeholders contributed to the intervention development process.	Potential stakeholders can include patient and community representatives, local and national policy makers, health care providers and those paying for or commissioning health care. Each of these groups may influence the intervention development process in different ways. Specifying how differing groups of stakeholders contributed to the intervention development process helps the reader to understand how stakeholders were involved and the degree of influence they had on the overall process. Further detail on how to integrate stakeholder contributions within intervention reporting are available.	p5-7	
Report how the intervention	Intervention development is frequently an iterative process. The conclusion of the initial	p15-16 /	

For peer review only

changed in content and format from the start of the intervention development process.	phase of intervention development does not necessarily mean that all uncertainties have been addressed. It is helpful to list remaining uncertainties such as the intervention intensity, mode of delivery, materials, procedures, or type of location that the intervention is most suitable for. This can guide other researchers to potential future areas of research and practitioners about uncertainties relevant to their healthcare context.	p9 vs. p12	
Report any changes to the intervention required or likely to be required for subgroups.	Specifying any changes that the intervention development team perceive are required for the intervention to be delivered or tailored to specific sub groups enables readers to understand the applicability of the intervention to their target population or context. These changes could include changes to personnel delivering the intervention, to the content of the intervention, or to the mode of delivery of the intervention.	p15-16	
Report important uncertainties at the end of the development process.	Intervention development is frequently an iterative process. The conclusion of the initial phase of intervention development does not necessarily mean that all uncertainties have been addressed. It is helpful to list remaining uncertainties such as the intervention intensity, mode of delivery, materials, procedures, or type of location that the intervention is most suitable for. This can guide other researchers to potential future areas of research and practitioners about uncertainties relevant to their healthcare context.	p15-16	
Follow TIDieR guidance when describing the developed intervention.	Interventions have been poorly reported for a number of years. In response to this, internationally recognized guidance has been published to support the high quality reporting of health care interventions and public health interventions. This guidance should therefore be followed when describing a developed intervention.		
Brief name.	Provide the name or a phrase that describes the intervention.	p4	
Rationale, theory and aim of the elements essential to the intervention.	Describe any rationale, theory, or goal of the elements essential to the intervention.	p6	Supplementary file B + [37]
Materials used in the intervention delivery or in the training of intervention providers.	Describe any physical or informational materials used in the intervention, including those provided to participants or used in intervention delivery or in training of intervention providers. Provide information on where the materials can be accessed (e.g. online appendix, URL).	p10-13	
Procedures, activities and/or processes used including enabling	Describe each of the procedures, activities, and/or processes used in the intervention, including any enabling or support activities.	p10-13	

For peer review only

or support activities.			
Expertise and background of intervention providers.	For each category of intervention provider (e.g. psychologist, nursing assistant), describe their expertise, background and any specific training given.	p10-11	
How the intervention was provided.	Describe the modes of delivery (e.g. face-to-face or by some other mechanism, such as internet or telephone) of the intervention and whether it was provided individually or in a group.	p10-13	
Location where the intervention occurred (incl. necessary infrastructure or relevant features).	Describe the type(s) of location(s) where the intervention occurred, including any necessary infrastructure or relevant features.	p5	
When and how much the intervention was delivered.	Describe the number of times the intervention was delivered and over what period of time including the number of sessions, their schedule, and their duration, intensity or dose.	p10 + 13	
Tailoring – what, why, when and how.	If the intervention was planned to be personalised, titrated or adapted, then describe what, why, when, and how.	p12-13	
Modifications. †	If the intervention was modified during the course of the study, describe the changes (what, why, when, and how).	N/A	
Strategies to improve or maintain fidelity.	If intervention adherence or fidelity was assessed, describe how and by whom, and if any strategies were used to maintain or improve fidelity, describe them.	p16	
Adherence and fidelity.†	If intervention adherence or fidelity was assessed, describe the extent to which the intervention was delivered as planned.	N/A	
Report the intervention development process in an open access format.	Unless reports of intervention development are available people considering using an intervention cannot understand the process that was undertaken and make a judgement about its appropriateness to their context. It also limits cumulative learning about intervention development methodology and observed consequences at later evaluation, translation and implementation stages. Reporting intervention development in an open access (Gold or Green) publishing format increases the accessibility and visibility of intervention development research and makes it more likely to be read and used. Potential platforms for open access publication of intervention development include open access journal publications, freely accessible funder reports or a study web-page that details the intervention development process.		Aim of this manuscript

Note. * e.g. if item is reported elsewhere, then the location of this information can be stated here. † If completing the TIDieR checklist for a protocol, these items are not relevant to the protocol and cannot be described until the study is complete.

For peer review only

For peer review only

BMJ Open

Development and content of a community-based reablement programme (I-MANAGE): A co-creation study

Journal:	*BMJ Open*
Manuscript ID	bmjopen-2022-070890.R1
Article Type:	Original research
Date Submitted by the Author:	23-Mar-2023
Complete List of Authors:	Mouchaers, Ines; Maastricht University, Department of Health Services Research; Living Lab of Ageing and Long Term Care<br>Verbeek, Hilde ; Maastricht University, Department of Health Services Research; Living Lab of Ageing and Long Term Care<br>Kempen, Gertrudis; Maastricht University, Department of Health Services Research; Living Lab of Ageing and Long Term Care<br>van Haastregt, Jolanda; Maastricht University, Department of Health Services Research; Living Lab of Ageing and Long Term Care<br>Vlaeyen, Ellen; KU Leuven, Public Health and Primary Care; UHasselt, Faculty of Medicine and Life Sciences<br>Goderis, Geert; KU Leuven, Department of Public Health and Primary Care<br>Metzelthin, Silke; Maastricht University, Department of Health Services Research; Living Lab of Ageing and Long Term Care
**Primary Subject Heading**:	Health services research
Secondary Subject Heading:	Patient-centred medicine, Rehabilitation medicine
Keywords:	Health Services for the Aged, Patient-Centered Care, Primary Health Care, Quality of Life, Rehabilitation medicine < INTERNAL MEDICINE

SCHOLARONE™
Manuscripts

I, the Submitting Author has the right to grant and does grant on behalf of all authors of the Work (as defined in the below author licence), an exclusive licence and/or a non-exclusive licence for contributions from authors who are: i) UK Crown employees; ii) where BMJ has agreed a CC-BY licence shall apply, and/or iii) in accordance with the terms applicable for US Federal Government officers or employees acting as part of their official duties; on a worldwide, perpetual, irrevocable, royalty-free basis to BMJ Publishing Group Ltd ("BMJ") its licensees and where the relevant Journal is co-owned by BMJ to the co-owners of the Journal, to publish the Work in this journal and any other BMJ products and to exploit all rights, as set out in our licence.

The Submitting Author accepts and understands that any supply made under these terms is made by BMJ to the Submitting Author unless you are acting as an employee on behalf of your employer or a postgraduate student of an affiliated institution which is paying any applicable article publishing charge ("APC") for Open Access articles. Where the Submitting Author wishes to make the Work available on an Open Access basis (and intends to pay the relevant APC), the terms of reuse of such Open Access shall be governed by a Creative Commons licence – details of these licences and which Creative Commons licence will apply to this Work are set out in our licence referred to above.

Other than as permitted in any relevant BMJ Author's Self Archiving Policies, I confirm this Work has not been accepted for publication elsewhere, is not being considered for publication elsewhere and does not duplicate material already published. I confirm all authors consent to publication of this Work and authorise the granting of this licence.

For peer review only

TITLE PAGE

Title

Development and content of a community-based reablement programme (I-MANAGE): A co-creation study

Authors

Ines Mouchaers, MSc, [1, 2, 3 *]

Hilde Verbeek, PhD, [1, 2]

Gertrudis I.J.M. Kempen, PhD, [1, 2]

Jolanda C.M. van Haastregt, PhD, [1, 2]

Ellen Vlaeyen, PhD, [3, 5, 6]

Geert Goderis, PhD, [4]

Silke F. Metzelthin, PhD, [1, 2]

Author Affiliations

[1] Department of Health Services Research, Faculty of Health Medicine and Life Sciences, CAPHRI Care and Public Health Research Institute, Maastricht University, Maastricht, the Netherlands

[2] Living Lab of Ageing and Long Term Care, Maastricht, the Netherlands

[3] Department of Public Health and Primary Care, Academic Centre for Nursing and Midwifery, KU Leuven, Leuven, Belgium

[4] Department of Public Health and Primary Care, Academic Centre for General Practice, KU Leuven, Leuven, Belgium

[5] Faculty of Medicine and Life Sciences, Hasselt University, Hasselt, Belgium

[6] University Centre for Nursing and Midwifery, Department of Public Health and Primary Care, Faculty of Medicine and Health Sciences, Ghent University, Belgium

For peer review only

For peer review only - http://bmjopen.bmj.com/site/about/guidelines.xhtml

Corresponding author

Ines Mouchaers: https://orcid.org/0000-0001-7189-4468

* Address correspondence to: Ines Mouchaers, MSc, Department of Health Services Research, P.O. Box 616, 6200 MD Maastricht, The Netherlands.

E-mail: i.mouchaers@maastrichtuniversity.nl; Telephone number: +31-(0)43 388 28 15.

Word count

4945

For peer review only

ABSTRACT

Objectives: With increasing age, people generally start experiencing problems related to independent living, resulting in an increased need for long-term care services. Investing in sustainable solutions to promote independent living is therefore essential. A concept attracting growing interest herein is reablement, a person-centred, holistic approach that promotes older adults' active participation through daily, social, leisure, and physical activities. The aim of this paper is to describe the development and content of I-MANAGE, a model for a reablement programme for community-dwelling older adults.

Design: The development of the programme was performed according to the Medical Research Council framework as part of the TRANS-SENIOR international training and research network. A co-creation design was used, including literature research, observations, interviews, and working group sessions with stakeholders.

Setting and participants: The interviews and working group sessions took place in the Dutch long-term home care context. Stakeholders invited to the individual interviews and working group sessions included care professionals, policymakers, client representatives, informal caregiver representatives, informal caregivers, and scientific experts.

Results: The co-creation process resulted in a 5-phase interdisciplinary primary care programme, called I-MANAGE. The programme focuses on improving the self-management and well-being of older people by working towards their meaningful goals. During the programme, the person's physical and social environment will be put to optimal use, and sufficient support will be provided to informal caregivers to reduce their burden. Lastly, the programme aims for continuity of care and better communication and coordination.

Conclusion: The I-MANAGE programme can be tailored to the local practices and resources and is therefore suitable for the use in different settings, nationally and internationally. If the programme is implemented as described, it is important to closely monitor the process and results.

For peer review only - http://bmjopen.bmj.com/site/about/guidelines.xhtml

STRENGTHS AND LIMITATIONS OF THIS STUDY

- Intervention development was conducted using a systematic approach based on the Medical Research Council framework.
- The intervention and its development is theory- and evidence-based as a result of extensive literature research.
- All end-users were represented during the development process, increasing its acceptability and feasibility.
- Data triangulation was used during the development process, increasing validity of the results.
- Client and informal caregiver representatives were included in the working group instead of clients and informal caregivers themselves, possibly missing an important voice.

For peer review only

BACKGROUND

The amount of older adults experiencing disabilities will increase over time, and while a large proportion of the older population remains independent, others will experience an increased need for support [1]. Moreover, 50% of people aged 85 years or older require care and/or support with daily activities [2]. As a result, it is expected that their demands for long-term care services will increase. When older people live in an environment that is not safe and does not meet their needs, the challenges they might face regarding independent living will become even bigger [3]. Previous research showed that a maladjusted environment negatively affects disability, which could lead to an accumulation of health risks, loss of independence, poor quality of life (QoL), and depression [4-6]. Care and service delivery in the community is often fragmented, with little coordination and poor communication amongst care providers, clients, and informal caregivers [3, 7-9]. Moreover, the focus of care is often on eliminating specific diseases and symptoms instead of supporting the remaining capacity to maintain quality of life and independent living [10, 11]. Care professionals often tend to take over care tasks rather than performing tasks with their clients [11]. Not tackling these challenges properly could lead to increased utilisation of health and social care and related costs [12], and unnecessary (re)hospitalisations or permanent nursing home placement, each with their own risks (e.g. increased mortality) [13], while financial and workforce resources are shrinking [7, 12, 14]. Therefore, it is essential to invest in sustainable solutions to promote independent living [15-17].

A concept attracting growing interest in promoting independent living among older people is reablement, a person-centred, holistic approach that promotes older adults' active participation in daily activities through social, leisure, and physical activities chosen by the older person in line with their preferences, either at home or in the community [18]. Instead of creating dependency by taking over tasks, care professionals identify the capabilities and opportunities of individuals to maximise their independence by supporting them to achieve their goals, through participation in daily activities, home modifications and assistive devices, and involvement of their social network [11, 18-20]. Current evidence on the effectiveness of reablement interventions is inconclusive [11]. However, several

For peer review only - http://bmjopen.bmj.com/site/about/guidelines.xhtml

For peer review only

systematic reviews have indicated that reablement has positive results related to activities of daily living (ADL) functioning and health-related QoL [21-23]. Because of its promising results, the interest in implementing reablement into everyday care is growing internationally. In Denmark, New Zealand and the United Kingdom reablement has been successfully implemented across more-or-less the whole country [1]. For example, in Denmark reablement in long-term care for older people is introduced by law since 2015, meaning that all municipalities must offer reablement interventions and all applicants for home care are assessed for potential for reablement, before being offered conventional home care [2].

Despite the promising results and successful implementation abroad, this is no indication that it is also effective in its current format in the Dutch home care setting due to contextual differences [24]. Implementing reablement is a complex process and if influenced by multiple factors, such as organisational factors, individual and social attitudes towards a new form of care, technological factors related to communication, and financial factors [25, 26]. It is crucial to critically consider these factors in the design, delivery, and evaluation of reablement [27]. Furthermore, to implement reablement in the Dutch home care setting, existing programmes need to be revised and adapted to suit the current context, which is crucial when developing and delivering complex health care interventions [27]. However, the development and content of community care programmes, such as reablement programmes, are often insufficiently described in the scientific literature [19, 28-30]. There are only limited articles available that describe either the development of the programme or its content in detail [31-33], and often these descriptions are included as part of a feasibility or pilot study [29]. This offers little guidance for replication of these programmes or to build on their previous findings, despite this being essential for the development of new and implementation of existing programmes in different settings [29, 30, 34].

Therefore, this paper describes the development, using a co-creation process, and content of I-MANAGE, a reablement programme for community-dwelling older adults to improve older persons' self-management and participation in daily life, while also increasing quality of life and decreasing

For peer review only - http://bmjopen.bmj.com/site/about/guidelines.xhtml

informal caregivers' burden. By describing the development and content of the programme in detail, we increase the replicability and prevent other researchers from re-inventing the wheel. The programme is specifically suitable for the Dutch community care setting, however, due to its extensive description; this manuscript may serve as a model for implementation in other countries as well.

For peer review only

For peer review only - http://bmjopen.bmj.com/site/about/guidelines.xhtml

METHODS

To describe the development process of the programme and ensure completeness of reporting, we used the guidance for reporting for intervention development studies in health research (GUIDED) (Supplemetary file 1) [29]. This checklist provides a clear and structured basis for the reporting of programme development, as well as the description of the content of the programme.

Design

I-MANAGE was developed between September 2019 and June 2021 using a co-creation design. The programme was developed following the first phase (development) of the Medical Research Council (MRC) framework for the development and evaluation of complex interventions [35, 36]. First, a logic model was created. A logic model is a tool to illustrate how a programme will create change [37]. The logic model systematically visualises the aim and sub-aims of the programme, the programme components, and the intended outcomes[37]. The logic model was developed using six iterative programme development steps: 1) identifying the problem, 2) identifying the evidence, 3) identifying or developing a theory, 4) determining needs, 5) examining current practices and the context, and 6) modelling processes and outcomes [38]. The logic model was then translated to practice (step 7).

Patient and public involvement

The co-creation was initiated by the researchers as a response to the challenges that are caused by an ageing society (e.g., increasing care needs, decreasing staff). To deal with these challenges the Dutch government stimulates an ageing in place policy and promotes another way of (home) care delivery from “doing for…” towards “doing with…” clients or in the best case to enable clients to do things by themselves again. This developments are also adopted by Dutch care organisations, which hope to improve the quality and sustainability of their care services [39]. All end-users (care professionals who would be implementing and delivering the programme, as well as the target population) were represented during and involved in the development process of the programme by being a member

For peer review only - http://bmjopen.bmj.com/site/about/guidelines.xhtml

For peer review only

of the working groups and participated in the individual interviews. They were not involved in the development of the study design of dissemination of the findings.

Setting and participants

I-MANAGE is based on international evidence and tailored to the Dutch home care context. Home care in the Netherlands includes personal care (i.e. assistance with activities of daily living (ADL)), nursing care (i.e. medical assistance), and domestic support (i.e. assistance with instrumental activities of daily living (IADL)) [40]. Usually home care is funded by two statutory forms of insurance cover care: the *Health Insurance Act* (ZVW), and the *Social Support Act* (WMO). Clients often use a combination of ZVW (e.g. general practitioner care, therapists, hospital care, or medication), and WMO (e.g. domestic support, home adaptations) [40, 41]. The programme was developed for community-dwelling older people, irrespective of age, cognitive and functional status.

The programme was developed in co-creation with stakeholders who participated in observations, individual interviews and working groups. Supplementary file 2 provides an overview of all stakeholders involved and the research activities they participated in. Participants were recruited from the professional network of the researchers. They were informed about the study and asked to participate via email. When participants agreed to participate, verbal or written informed consent was provided before the start of each interview or working group session.

Data collection

Data collection was performed following a non-linear and iterative process as described by Bleijenberg et al. [38], and by using a variety of data collection methods including literature research, observations, individual interviews, and working groups. Figure 1 illustrates the development process in detail.

For peer review only - http://bmjopen.bmj.com/site/about/guidelines.xhtml

Development of a logic model (steps 1 to 6)

All steps were guided by literature research, especially steps 2 and 3 of the development process. To gain insight into current evidence-based practices (*step 2*), the relevant scientific literature on reablement programmes was reviewed. The methodology and results of this literature research is described elsewhere [42]. To identify a theoretical foundation for the programme (*step 3*), a literature review was performed on the concept of disability and its underlying causal mechanisms [43].

Furthermore, a working group was composed and invited to three sessions (1.5 h each). Members of the working group included physiotherapists (PT), occupation therapists (OT), registered nurses (RN), a domestic support worker (DSW), a policymaker of the local municipality, a psychologist, a client representative, informal caregiver representatives, and a geriatric rehabilitation expert. The first session focused on the identification of the problem (*step 1*) and the examination of the current practice and context (*step 5*). Session two focused on the previously identified evidence, which participants could complement with practices from their own work field (*step 2*), and the determination of needs (*step 4*), closely related to the previously identified problems. The third session focused on modelling the process and outcomes (*step 6*), during which a preliminary logic model was presented on which participants could provide feedback. Between sessions, working group members were consulted for additional input and clarification if needed. The researchers processed the results from each session and the additional information in order to be used as a starting point for the next session. The working group sessions were led by the first and last author (IM and SM).

We also interviewed two PTs, a policy maker of the local municipality, a client and informal caregiver representative, and three experts in the field of geriatric rehabilitation research. In total, eight interviews were conducted, all focusing on identifying the problem (*step 1*), determining the needs (*step 4*), and gaining insight into current practice and context (*step 5*). Additionally, we interviewed two informal caregivers. Topics that were addressed during these interviews included their role as informal caregiver, how involved they were in the care process, how they were or felt supported and by whom, and what they would like to change in the process.

For peer review only - http://bmjopen.bmj.com/site/about/guidelines.xhtml

Lastly, observations were performed by the researchers. The observations were conducted in the traditional community care setting to examine current practice and context of home care services (*step 5*) and to identify problems (*step 1*). Six observations of half a day each were performed by shadowing a DSW, an RN, and a nursing assistant, and three days with allied health. These observations resulted in field notes.

Translation to practice (step 7)

For each of the six programme components in the logic model, members of the working group were invited, as well as a geriatrician, consultant informal care, reablement researcher, informal caregiver, and a manager of community care teams, to a session to translate components into practice (*step 7*). Based on the programme component, the most relevant stakeholders were invited. For example, on informal caregiver support, we invited a consultant informal care, an informal caregiver, and a psychologist; resulting in three to four members per working group. Each working group attended one session (1 h). Each session began with an introduction to the goal of the programme, the logic model, and the results of steps 1 to 6 related to the programme component. Afterwards, participants were asked open-ended questions about the practical implementation of the component, barriers, and facilitators. Lastly, results were summarised and participants were asked for final feedback. Table 1 provides an overview of the different methodologies used in each development step.

Table 1. Overview of the different methodologies used in each development step defined by Bleijenberg et al. [38]

Programme development steps	Methodology			
	Literature research	Observations	Individual interviews	Working groups
1. Problem identification	● (grey)	●	●	●
2. Identifying the evidence	●			●
3. Identifying or developing theory	●			
4. Determine the needs	● (grey)		●	●
5. Examining current practice and context	● (grey)	●	●	●
6. Modelling process and outcomes	● (grey)	● (grey)	● (grey)	●
7. Translation to practice	● (grey)			●

Note: The full black dots ● indicate a main source of information for the particular step, while the full grey dots ● indicate only a minor influence and guidance for the particular step.

For peer review only - http://bmjopen.bmj.com/site/about/guidelines.xhtml

[Insert Figure 1 here]

Data analysis

We used data triangulation to verify the results. Our main source of information was the working group sessions. Sessions were recorded and transcribed non-verbatim. A thematic analysis was conducted. Common themes were identified within each step of the development process (e.g. lack of sufficient communication). Afterwards, the themes from all sessions were compared and searched for associations between, for example, identified problems and determined needs, or current scientific evidence and best practices of the organisation. The results of each session provided input and structure for the next session. Individual interviews were recorded and transcribed non-verbatim. Individual interviews, observations, and literature research were used to complement and check the information obtained through the working group sessions. After each working group session or individual interview, a member check was conducted by summarising the most important findings. The results were discussed regularly within the research team. The research team consists of all authors involved. They work in the field of social gerontology, public health and primary care, and long-term care. A research logbook was kept by the researchers to keep track of all research activities and intermediate results, together with field notes from these activities.

For peer review only

For peer review only - http://bmjopen.bmj.com/site/about/guidelines.xhtml

RESULTS

The following section presents the final model of the I-MANAGE programme in detail, starting with the developed logic model, and eventually the translation to practice, which contains a detailed description of all programme components. A detailed description of the results from the first five programme development steps is provided in Supplementary file 3.

Development of a logic model

The logic model starts with the aim and sub-aims of the programme based on identified problems and needs (*result from development steps 1 and 4*), which are derived mostly from the working group sessions and individual interviews. To fulfil these aims, six programme components were determined: 1) improving assessment and goal-setting, 2) stimulating self-management during meaningful daily activities, 3) optimising the use of the physical environment, 4) optimising the use of the social environment, 5) improving interprofessional collaboration, and 6) engaging the informal caregiver. These originate from both the input from stakeholders (*result from development steps 2, 4, 5 and 6)* and evidence-based practices from the literature (*result from development step 2*). The intended client outcomes of the programme are reducing (I)ADL disability, improving self-management skills, increasing the quality of life of both the client and informal caregiver, and reducing healthcare utilisation and expenditures (proximal outcomes), which are common outcome measures in reablement programmes abroad, to remain living at home independently and avoid unnecessary transitions to institutional care (distal outcomes). Figure 2 presents the logic model.

[Insert Figure 2 here]

Translation to practice: description of the I-MANAGE programme

The I-MANAGE programme is the result of input from stakeholders during the last step of the development process (*step 7*) [38]. The following sections describe the programme in detail. The programme, as described, may serve as a model for the use in different local settings and leaves room for tailoring to the specific needs and resources of the organisation.

For peer review only - http://bmjopen.bmj.com/site/about/guidelines.xhtml

To achieve the intended outcomes presented in the logic model, the programme applies a two-tiered approach: 1) focusing on the capabilities of the client and 2) paying attention to environmental factors (i.e. physical, social, and organisational). I-MANAGE is intended for community-dwelling older people who are still able to (re)learn new skills and apply them in daily life without an indication of terminal illness or planned nursing home admission. The average duration of the programme is 8 weeks.

Interdisciplinary collaboration

I-MANAGE facilitates interdisciplinary collaboration by facilitating intensive collaboration between the reablement team, client, informal caregiver, and other care professionals delivering care and support to the client and informal caregiver. The programme is delivered by an interdisciplinary reablement team, generally consisting of an RN, an OT, and, depending on the local context, supplemented with a PT, social worker, or DSW. The reablement team initiates the programme and is responsible for the direct support of the client and informal caregiver. A care coordinator is appointed, in most cases, the OT. Depending on the necessary care and goals set by the client, the reablement team may consult other care professionals (e.g. usual home care team, psychologist, or general practitioner), for example, during the intake phase for advice or while working on the client's goals. They are informed and coached by the reablement team.

Moreover, (bi-)weekly team meetings are organised to discuss the intake of new clients and informal caregivers, the progress made by clients, and final evaluations of clients' personal goals. Additionally, team meetings provide the opportunity to discuss specific cases with other team members, and to organise training. All members of the reablement team should have access to the client's electronic care file (ECF) to access the care plan, report progress, and follow up on each other's work. Lastly, when discharged from another care facility (e.g. hospital or rehabilitation facility), a smooth handover of client information is initiated so no necessary details of the client's care process are lost.

For peer review only - http://bmjopen.bmj.com/site/about/guidelines.xhtml

Practice-oriented training

Preceding the programme, the reablement team receives practice-oriented training focusing on adequately delivering I-MANAGE. Care professionals receive a detailed manual beforehand, consisting of background information and a description of the programme, including the goal, key components, and I-MANAGE's care process. The manual was developed by the researchers and revised by working group members to make sure it is suitable for practice. Additionally, a toolkit for achieving individual client goals is provided (e.g. exercise booklet based on the OTAGO exercise programme [44], social map including welfare initiatives, and tools to assess needs and wishes of the caregiver). The training consists of multiple sessions. The first is a kick-off meeting for the reablement team, focusing on the goal and content of I-MANAGE. Second, specific training sessions occur for OT and PT concerning the use of the Canadian Occupational Performance Measure (COPM) [45] to set personal goals with the client, and the OTAGO exercise booklet, respectively. Finally, the reablement team receives a booster session to practice and discuss challenging situations and focus on motivational and conversation skills. The kick-off and booster sessions each last two hours, and the specific sessions for OT and PT last one hour. Additionally, the programme stimulates training on the job, meaning that members of the reablement team can coach each other and external care professionals based on their own expertise.

For peer review only

I-MANAGE care process

The I-MANAGE care process consists of five consecutive phases: 1) initiation, 2) intake, 3) care plan, 4) care delivery, and 5) evaluation. Each phase is described in detail below and is presented in Figure 3. The five phases are a result of the practical translation of the logic model described earlier.

Phase 1: Initiation

For peer review only - http://bmjopen.bmj.com/site/about/guidelines.xhtml

People are referred to the programme through community nurses, general practitioners, or institutional care facilities. Community-dwelling older adults eligible for I-MANAGE receive information about the programme. The care coordinator plans a first visit to present the programme to the client and their informal caregiver, and provides the opportunity to ask additional questions. The care coordinator initiates an exploratory conversation based on the principles of positive health to gain insight into the client's needs and wishes [46].

Phase 2: Intake

Within the first week after the initiation phase, the OT performs an environmental assessment identifying necessary home modifications and assistive devices to ensure a safe environment. Additionally, the care coordinator or an assigned social worker performs an intake with the informal caregiver assessing their needs and wishes. Furthermore, the OT sets meaningful goals with the client using COPM [45] (maximum of five goals). Clients must score both their performance and their satisfaction with performing these activities [45]. Setting goals is guided by the exploratory conversation held in phase 1. The ultimate goal is to improve the client's participation and well-being; therefore, goals are not merely (I)ADL-related (e.g. meeting friends at the local café, or painting in the hobby room on the first floor). The goals are recorded in the client's ECF.

Phase 3: Care plan

Possible interventions and actions to achieve the client's goals and provide the informal caregiver with the right support are discussed within the reablement team. These interventions and actions are derived from the available toolkit. Afterwards, the care coordinator determines the final interventions and actions with the client and informal caregiver, guided by their preferences and capabilities, possibilities of the social network, and physical environment. Subsequently, the care plan is recorded in the client's ECF and shared with all members of the reablement team. The intensity of the programme depends on the care needs of the client and their pre-set goals, which may require a higher intensity in the beginning but less at the end when (sub-)goals are (partly) reached.

For peer review only - http://bmjopen.bmj.com/site/about/guidelines.xhtml

For peer review only

Phase 4: Care delivery

The reablement team delivers care and support, as described in the care plan. Care delivery is coordinated by the OT, who is also responsible for (re)learning and practicing meaningful daily activities. The RN is responsible for supporting managing the client's personal care needs. The PT, when part of the reablement team, is responsible for functional training and stimulating participation in daily life. The client's environment is adapted (e.g. (home) modifications, assistive devices, and care technology), to support them in (re)learning activities. During the support with these meaningful activities, the client's self-management is stimulated by practicing these activities and ensuring that tasks are not taken over by care professionals or informal caregivers. If necessary, a social worker provides the informal caregiver with additional support decreasing their burden (e.g. respite care).

Phase 5: Evaluation

The evaluation of the care process is structurally embedded in interdisciplinary team meetings. Based on the conclusions drawn during these meetings, the care coordinator decides whether the care plan is continued, adjusted, or terminated. This is discussed with the client and informal caregiver. At the end of the programme, a formal evaluation of the client's goals takes place using COPM [45]. Afterwards, the care coordinator decides with the client and informal caregiver if the programme should be extended (max. two weeks) to ensure all goals are sufficiently reached or if the client needs referral to usual care.

[Insert Figure 3 here]

DISCUSSION

This manuscript describes the development and content of I-MANAGE, a community-based reablement programme. The programme was developed for community-dwelling older adults to

For peer review only - http://bmjopen.bmj.com/site/about/guidelines.xhtml

For peer review only

improve their self-management and participation in daily life and ensure that they can remain living at home independently as long as possible and avoid unnecessary transitions to institutional care, while also increasing quality of life and decreasing the burden of informal caregivers.

The programme contains several key elements that are considered essential. First, interdisciplinary collaboration is important in I-MANAGE (e.g. appointing a care coordinator, scheduling (bi-)weekly meetings, and a shared ECF). Recent literature indicates that investing in interdisciplinary collaboration stimulates patient-centred care by ensuring a holistic view of the client's situation and creates shared responsibility [47-49]. This is achieved by good communication and coordination within the team but also with the client and informal caregiver [50]. Moreover, delivery of the programme by an interdisciplinary team, including allied health, such as OTs, is deemed valuable because of their educational background [30, 51]. It also stimulates continuous learning and is experienced as exciting and constructive by care professionals [49, 50]. In combination with the practice-oriented training that is integrated in the programme, and training on the job, where care professionals have the chance to learn from other disciplines, helps to invest in the self-efficacy of care professionals, which is essential because it remains a challenge to change behaviour successfully . Second, in the beginning of the programme (phase 1 and 2), we implemented a standardised goal-setting tool, COPM [45], preceded by an extensive intake based on the principles of positive health [46]. Previous research has indicated that using standardised assessment or goal-setting tools could increase the effectiveness of reablement interventions [30]. Additionally, it increased client involvement and helps professionals to identify meaningful activities with the client [52]. Third, when delivering care (phase 4), the programme integrates several important aspect. First, supporting informal caregivers also contributes to the success of I-MANAGE. Previous research found that providing informal caregivers with the right psychosocial and educational support strengthens their ability to cope with their new role [53, 54]. This is an important addition to most reablement programmes, because although making use of the clients' social network is a strategy to reach their goals according to the internationally accepted definition [18], this is often overlooked [55, 56].

For peer review only - http://bmjopen.bmj.com/site/about/guidelines.xhtml

Additionally, I-MANAGE promotes optimal use of the social and physical environment, which is essential since a demanding environment can either stimulate or hinder a person's participation in meaningful activities [3, 57]. Lastly, the programme stimulates self-management through participation in meaningful daily activities, which is a core element within reablement ('doing with…' rather than 'doing for…' the client) [18, 58].

The described programme serves as a model and leaves room to tailor the intervention to a specific context and the needs of the organisation. For example, the programme leaves room to the organisation to choose which target group would benefit the most. Additionally, delivering the programme by an interdisciplinary team is a prerequisite; however, depending on the available resources, the organisation can decide on the composition of the reablement team. Moreover, I-MANAGE is a multi-component programme in which organisations can integrate their own innovative practices, especially when working towards and reaching clients' meaningful goals. Moe and Brinchmann [25] confirmed the necessity of tailoring reablement services to local conditions by arguing that establishing reablement in an existing organisational structure is a complex process. Apart from available resources, during the co-creation process, some influencing factors for implementation were identified. For example, different IT systems, making communication and information transfer less evident. Moe and Brinchmann [25] mentioned that, next to communication and IT systems, habitual ways of offering health services and lacking knowledge about the rehabilitation potential of older people and benefits of active ageing are also experienced as barriers. A recent scoping review identified several factors that act as barriers and facilitators during the implementation of care innovations, among which available resources and communication were listed as possible barriers to implementation [59]. It is critical to consider these factors when implementing I-MANAGE in practice. In order to overcome several barriers to the implementation of I-MANAGE, it could be useful to set up advisory boards and working groups to discuss the implementation process and adjust where needed. Additionally, investing in getting the management of care organisations on board to support the implementation within their organisation and setting up knowledge exchange between sites where

For peer review only - http://bmjopen.bmj.com/site/about/guidelines.xhtml

reablement is being implemented to share experiences and best practices could be beneficial. Investing in a suitable organisational structure is essential because it remains a challenge to change existing organisational structures successfully [25, 60, 61]. Regarding future research, (cost-)effectiveness has not yet been proven, but is currently being implemented and evaluated at different Dutch care providers. The systematic reporting of the development of the programme provides useful insight for future research looking to develop complex health interventions or to implement a similar programme.

A strength of I-MANAGE and its development is that it is theory- and evidence-based [42, 43], which has proven to be advantageous when developing effective interventions [62, 63]. Moreover, Thuesen et al. [64] highlight the demand for making theory explicit in reablement interventions, and addressing the physical, psychological, and sociocultural perspectives of ageing within these interventions. Another strength of the development process was the co-creation process, giving a voice to multiple stakeholders and making the programme suitable for practice. We used the development approach described by Bleijenberg et al. [38], which combined a range of published approaches to intervention development to enrich the MRC framework. This approach was chosen because using the MRC framework would help us in further evaluating and adapting the programme. However, we are aware that multiple approaches to intervention development exist as described by O'Cathain et al. [65]. These different approaches share many similarities (e.g. stepwise approach or involvement of stakeholders), but there are also significant differences (e.g. the focus on implementation or theory). It is important to acknowledge these differences and always chose an approach best suited for the purpose of the research. Additionally, most of these approaches are not set in stone and leave room for the researchers own interpretation. It also has to fit the setting and timing of the development process. There are also some limitations related to this co-creation process. First, during the process, we always informed management to obtain their support, but they were not included in the working groups, which could have been an important opinion lacking. Second, we aimed to include all end users of the programme during the development process (care professionals,

For peer review only - http://bmjopen.bmj.com/site/about/guidelines.xhtml

clients, and informal caregivers). However, we only included client and informal caregiver representatives in the working groups and interviewed only two informal caregivers between sessions. Additionally, the care professionals were recruited from the professional network of the researchers and were recruited in a convenient way. We did not ensure variation among these participants in terms of, for example, gender, age, or years of experience. Last, we also obtained data from many different sources (i.e. scientific literature, individual interviews, working groups, and observations), which made it difficult to find common ground throughout all the sources and forced us to compromise. However, this use of data triangulation is also a strength of the development of the programme because we were able to check our findings multiple times with different sources, increasing the validity of the results.

For peer review only

For peer review only - http://bmjopen.bmj.com/site/about/guidelines.xhtml

FIGURE LEGENDS

Figure 1: The development process over time. This figure presents the development process over time from September 2019 until June 2021. The whole process is guided by scientific literature. The figure presents the two parts of the development process each with their related activities. The dark grey rectangles present the (intermediate) result of each part of the process. The number in between brackets represent the seven steps of the development phase as described by Bleijenberg et al. [38]. PT = physiotherapist; GR = geriatric rehabilitation; DSW = domestic support worker; RN = registered nurse.

Figure 2: Logic model of the I-MANAGE programme. The logic model of the I-MANAGE programme presents the aim and associated sub-aims of the programme, the programme components, and proximal and distal outcomes.

Figure 3: Detailed presentation of the I-MANAGE care process. I-MANAGE has a 5-phase care process (initiation, intake, care plan, care delivery, and evaluation), preceded with the referral of the client through different routes. After an average duration of 8 weeks, clients are referred to aftercare. Interdisciplinary collaboration is a continuous element of the programme.

ETHICS APPROVAL AND CONSENT TO PARTICIPATE

As participants were not being subjected to actions and no rules of behaviour were imposed on them no ethical approval was needed according to the Dutch 'Medical Research Involving Human Subjects Act' (WMO). Nevertheless the study was carried out in accordance with the principles of ethical research and all participants involved in the working group sessions and interviews provided either written or verbal informed consent prior to the start of each session.

AVAILABILITY OF DATA AND MATERIALS

The datasets used and/or analysed during the current study available from the corresponding author on reasonable request.

For peer review only

COMPETING INTEREST

The authors declare that they have no competing interests

FUNDING

This work was supported by the European Union's Horizon 2020 research and innovation programme under the Marie Skłodowska-Curie grant agreement No 812656 as part of the TRANS-SENIOR project (www.trans-senior.eu).

AUTHORS' CONTRIBUTORS

All authors – IM, HV, GIJMK, JCMvH, EV, GG, SFM – were involved in the development of the I-MANAGE programme. IM wrote the first draft of the article. All authors reviewed the manuscript and approved the final version.

ACKNOWLEDGEMENTS

We would like to thank the Dutch stakeholders who have participated in the working group sessions, observations, and individual interviews, and therefore have helped with the development of a reablement programme suitable for the Dutch home care setting: Angelica de Lang, Nelly Jacobs, Jose Blezer, Belkis Bozdemir, Marcel Mannens, Marc Lowis, Maud Scholtes, Mandy Boosten, Hella Senden, Angèle van Kasteren, Anne Gulpers, Eveline van Velthuijsen, Anouschka Feenstra, Elfi Pakbeers, Nadine Klinkers, Marjo Gartener (MeanderGroep Zuid-Limburg), Ewout Smit (Vivium Zorggroep), Teuni Rooijackers, Angelique de Rijk (Maastricht University), Bianca Miltenburg (Cicero Zorggroep), Marie-Therese Bindels, Femke Scheuerman (gemeente Kerkrade), Marja Veenstra (Burgerkracht Limburg).

SUPPLEMENTAL MATERIAL

For peer review only - http://bmjopen.bmj.com/site/about/guidelines.xhtml

Supplementary file 1: GUIDED checklist. Completed guideline for reporting for intervention development studies.

Supplementary file 2: Overview of the different stakeholders involved per research activity.

Supplementary file 3: Overview of the intermediate results of the first five steps of the development phase.

REFERENCES

1. Zingmark M, Norström F. Transitions between levels of dependency among older people receiving social care – a retrospective longitudinal cohort study in a Swedish municipality. BMC Geriatr. 2021 2021/06/02;21(1):342.
2. Brown GC. Living too long: the current focus of medical research on increasing the quantity, rather than the quality, of life is damaging our health and harming the economy. EMBO Rep. 2015;16(2):137-41.
3. Rustad EC, Furnes B, Cronfalk BS, et al. Older patients' experiences during care transition. Patient Prefer Adherence. 2016;10:769-79.
4. Wahl HW, Fange A, Oswald F, et al. The home environment and disability-related outcomes in aging individuals: What is the empirical evidence? Gerontologist. 2009 Jun;49(3):355-67.
5. Arnau A, Espaulella J, Serrarols M, et al. Risk factors for functional decline in a population aged 75 years and older without total dependence: A one-year follow-up. Arch Gerontol Geriatr. 2016;65:239-47.
6. Tappenden P, Campbell F, Rawdin A, et al. The clinical effectiveness and cost-effectiveness of home-based, nurse-led health promotion for older people: A systematic review. Health Technol Assess. 2012;16(20):1-72.
7. Fujisawa R, Colombo F. The Long-Term Care Workforce: Overview and Strategies to Adapt Supply to a Growing Demand. 2009.
8. Arbaje AI, Kansagara DL, Salanitro AH, et al. Regardless of age: Incorporating principles from geriatric medicine to improve care transitions for patients with complex needs. J Gen Intern Med. 2014;29(6):932-9.
9. Koopmans RT, Lavrijsen JC, Hoek JF, et al. Dutch elderly care physician: a new generation of nursing home physician specialists. J Am Geriatr Soc. 2010;58(9):1807-9.
10. Nicholson C, Meyer J, Flatley M, et al. The experience of living at home with frailty in old age: A psychosocial qualitative study. Int J Nurs Stud. 2013 2013/09/01/;50(9):1172-9.

For peer review only

11. Aspinal F, Glasby J, Rostgaard T, et al. New horizons: Reablement - supporting older people towards independence. Age Ageing. 2016;45(5):572-6.
12. Beard JR, Officer A, de Carvalho IA, et al. The World report on ageing and health: a policy framework for healthy ageing. The Lancet. 2016;387(10033):2145-54.
13. Ferrah N, Ibrahim JE, Kipsaina C, et al. Death Following Recent Admission Into Nursing Home From Community Living: A Systematic Review Into the Transition Process. J Aging Health. 2018;30(4):584-604.
14. Oliver D, Foot C, Humphries R. Making our health and care systems fit for an ageing population: King's Fund London: UK; 2014.
15. Wiles JL, Leibing A, Guberman N, et al. The meaning of "aging in place" to older people. Gerontologist. 2012 Jun;52(3):357-66.
16. Cesari M, Vellas B, Hsu F-C, et al. A physical activity intervention to treat the frailty syndrome in older persons-results from the LIFE-P study. J Gerontol A Biol Sci Med Sci. 2015;70(2):216-22.
17. Daniels R, Metzelthin SF, van Rossum E, et al. Interventions to prevent disability in frail community-dwelling older persons: An overview. Eur J Ageing. 2010;7(1):37-55.
18. Metzelthin SF, Rostgaard T, Parsons M, et al. Development of an internationally accepted definition of reablement: a Delphi study. Ageing and Society. 2020:1-16.
19. Doh D, Smith R, Gevers P. Reviewing the reablement approach to caring for older people. Ageing and Society. 2019:1-13.
20. Metzelthin SF, Zijlstra GA, van Rossum E, et al. 'Doing with ...' rather than 'doing for ...' older adults: rationale and content of the 'Stay Active at Home' programme. Clin Rehabil. 2017;31(11):1419-30.
21. Tessier A, Beaulieu M-D, McGinn CA, et al. Effectiveness of Reablement: A Systematic Review. Healthcare policy. 2016;11(4):49-59.
22. Whitehead PJ, Worthington EJ, Parry RH, et al. Interventions to reduce dependency in personal activities of daily living in community dwelling adults who use homecare services: A systematic review. Clin Rehabil. 2015 Nov;29(11):1064-76.
23. Resnick B, Galik E, Boltz M. Function focused care approaches: Literature review of progress and future possibilities. J Am Med Dir Assoc. 2013 2013/05/01/;14(5):313-8.
24. Parsons J, Burton E, Graff L, et al. 4: Reablement as an evolution in home care: a comparison of implementation across five countries. In: Rostgaard T, Parsons J, Tuntland H, editors. Reablement in Long-Term Care for Older People. Bristol: Policy Press; 2023. p. 68-92.

For peer review only - http://bmjopen.bmj.com/site/about/guidelines.xhtml

25. Moe C, Brinchmann BS. Tailoring reablement: A grounded theory study of establishing reablement in a community setting in Norway. Health Soc Care Community. 2018 Jan;26(1):113-21.

26. Wess T, Steiner W, Dür M, et al. Reablement – relevant factors for implementation: an exploratory sequential mixed-methods study design. BMC Health Serv Res. 2022 2022/07/28;22(1):959.

27. Rostgaard T, Tuntland H, Parsons J. 1: Introduction: The concept, rationale, and implications of reablement. Reablement in Long-Term Care for Older People. Bristol: Policy Press; 2023. p. 3-20.

28. Mjøsund HL, Moe CF, Burton E, et al. Integration of Physical Activity in Reablement for Community Dwelling Older Adults: A Systematic Scoping Review. J Multidiscip Healthc. 2020;13:1291-315.

29. Duncan E, Cathain A, Rousseau N, et al. Guidance for reporting intervention development studies in health research (GUIDED): an evidence-based consensus study. BMJ Open. 2020;10(4):e033516.

30. Buma LE, Vluggen S, Zwakhalen S, et al. Effects on clients' daily functioning and common features of reablement interventions: a systematic literature review. Eur J Ageing. 2022 2022/05/03.

31. Assander S, Bergström A, Eriksson C, et al. ASSIST: a reablement program for older adults in Sweden – a feasibility study BMC Geriatr. 2022 2022/07/26;22(1):618.

32. Bergström A, Borell L, Meijer S, et al. Evaluation of an intervention addressing a reablement programme for older, community-dwelling persons in Sweden (ASSIST 1.0): a protocol for a feasibility study. BMJ Open. 2019;9(7):e025870.

33. Zingmark M, Kylén M. Feasibility of a reablement-program in a Swedish municipality. Scand J Occup Ther. 2023 2023/01/02;30(1):53-64.

34. Hoffmann TC, Glasziou PP, Boutron I, et al. Better reporting of interventions: template for intervention description and replication (TIDieR) checklist and guide. BMJ : British Medical Journal. 2014;348:g1687.

35. Craig P, Dieppe P, Macintyre S, et al. Developing and evaluating complex interventions: the new Medical Research Council guidance. BMJ. 2008;337:a1655.

36. Skivington K, Matthews L, Simpson SA, et al. A new framework for developing and evaluating complex interventions: update of Medical Research Council guidance. BMJ. 2021;374:n2061.

37. Centers for Disease Control and Prevention. Evaluation guide. Developing and using a logic model. CDC Division for Heart Disease and Stroke Prevention.

For peer review only - http://bmjopen.bmj.com/site/about/guidelines.xhtml

38. Bleijenberg N, de Man-van Ginkel JM, Trappenburg JCA, et al. Increasing value and reducing waste by optimizing the development of complex interventions: Enriching the development phase of the Medical Research Council (MRC) Framework. Int J Nurs Stud. 2018;79:86-93.

39. Bigonnesse C, Chaudhury H. The Landscape of "Aging in Place" in Gerontology Literature: Emergence, Theoretical Perspectives, and Influencing Factors. Journal of Aging and Environment. 2020 2020/07/02;34(3):233-51.

40. Kroneman M, Boerma W, van den Berg M, et al. Netherlands: Health System Review. Health Syst Transit. 2016 Mar;18(2):1-240.

41. Genet N, Boerma W, Kroneman M, et al. Home Care Across Europe. Current Structure and Future Challenges2013.

42. Mouchaers I, Van Haastregt JCM, Verbeek H, et al. « Reablement » comme outil thérapeutique pour offrir aux personnes âgées une vie de qualité à domicile après une réadaptation gériatrique. Rev Med Brux. 2021;42(4):324-32.

43. Mouchaers I, Verbeek H, Kempen GIJM, et al. The concept of disability and its causal mechanisms in older people over time from a theoretical perspective: a literature review. Eur J Ageing. 2022 2022/01/29.

44. Campbell AJ, Robertson MC. Het Otago Oefenprogramma om valincidenten bij ouderen te voorkomen. Amersfoort: Nederlandse Vereniging voor Fysiotherapie in de Geriatrie; 2013.

45. Law M, Baptiste S, McColl M, et al. The Canadian Occupational Performance Measure: An Outcome Measure for Occupational Therapy. Canadian Journal of Occupational Therapy. 1990;57(2):82-7.

46. Huber M, van Vliet M, Giezenberg M, et al. Towards a 'patient-centred' operationalisation of the new dynamic concept of health: a mixed methods study. BMJ Open. 2016;6(1):e010091.

47. Schmutz JB, Meier LL, Manser T. How effective is teamwork really? The relationship between teamwork and performance in healthcare teams: a systematic review and meta-analysis. BMJ Open. 2019;9(9):e028280.

48. Ebrahimi Z, Patel H, Wijk H, et al. A systematic review on implementation of person-centered care interventions for older people in out-of-hospital settings. Geriatric Nursing. 2021 2021/01/01/;42(1):213-24.

49. Eliassen M, Moholt JM. Boundary work in task-shifting practices - a qualitative study of reablement teams. Physiother Theory Pract. 2022 Apr 18:1-14.

50. Birkeland A, Tuntland H, Førland O, et al. Interdisciplinary collaboration in reablement - a qualitative study. J Multidiscip Healthc. 2017;10:195-203.

For peer review only - http://bmjopen.bmj.com/site/about/guidelines.xhtml

51. Reeves S, Fletcher S, Barr H, et al. A BEME systematic review of the effects of interprofessional education: BEME Guide No. 39. Med Teach. 2016 Jul;38(7):656-68.
52. Rose A, Rosewilliam S, Soundy A. Shared decision making within goal setting in rehabilitation settings: A systematic review. Patient Educ Couns. 2017 Jan;100(1):65-75.
53. Lopez-Hartmann M, Wens J, Verhoeven V, et al. The effect of caregiver support interventions for informal caregivers of community-dwelling frail elderly: a systematic review. Int J Integr Care. 2012 Jul-Sep;12:e133.
54. Spiers GF, Liddle J, Kunonga TP, et al. What are the consequences of caring for older people and what interventions are effective for supporting unpaid carers? A rapid review of systematic reviews. BMJ Open. 2021;11(9):e046187.
55. Hjelle KM, Alvsvåg H, Førland O. The relatives' voice: how do relatives experience participation in reablement? A qualitative study. Journal of multidisciplinary healthcare. 2016;10:1-11.
56. van Wieringen M, Broese van Groenou MI, Groenewegen P. Impact of home care management on the involvement of informal caregivers by formal caregivers. Home Health Care Serv Q. 2015 Apr-Jun;34(2):67-84.
57. de Vries NM, Staal JB, van Ravensberg CD, et al. Outcome instruments to measure frailty: A systematic review. Ageing Res Rev. 2011 2011/01/01/;10(1):104-14.
58. Clotworthy A, Kusumastuti S, Westendorp RGJ. Reablement through time and space: a scoping review of how the concept of 'reablement' for older people has been defined and operationalised. BMC Geriatr. 2021 2021/01/15;21(1):61.
59. Fakha A, Groenvynck L, de Boer B, et al. A myriad of factors influencing the implementation of transitional care innovations: a scoping review. Implementation Science. 2021 2021/02/26;16(1):21.
60. Reay T, Goodrick E, D'Aunno T. Health Care Research and Organization Theory. Cambridge: Cambridge University Press; 2021. Available from: https://www.cambridge.org/core/elements/health-care-research-and-organization-theory/18F375C5D5DEBE92B725FAB547EA076D.
61. Rooijackers T, van Rossum E, Zijlstra GAR, et al. Effectiveness of a reablement training program on self-efficacy and outcome expectations regarding client activation in homecare staff: A cluster randomized controlled trial. Geriatric Nursing. 2022 2022/01/01/;43:104-12.
62. Michie S, Prestwich A. Are interventions theory-based? Development of a theory coding scheme. Health Psychol. 2010 Jan;29(1):1-8.
63. Bartholomew LK, Mullen PD. Five roles for using theory and evidence in the design and testing of behavior change interventions. J Public Health Dent. 2011;71(s1):S20-S33.

For peer review only - http://bmjopen.bmj.com/site/about/guidelines.xhtml

64. Thuesen J, Feiring M, Doh D, et al. Reablement in need of theories of ageing: would theories of successful ageing do? Ageing and Society. 2021:1-13.

65. O'Cathain A, Croot L, Sworn K, et al. Taxonomy of approaches to developing interventions to improve health: a systematic methods overview. Pilot and Feasibility Studies. 2019 2019/03/12;5(1):41.

For peer review only

For peer review only - http://bmjopen.bmj.com/site/about/guidelines.xhtml

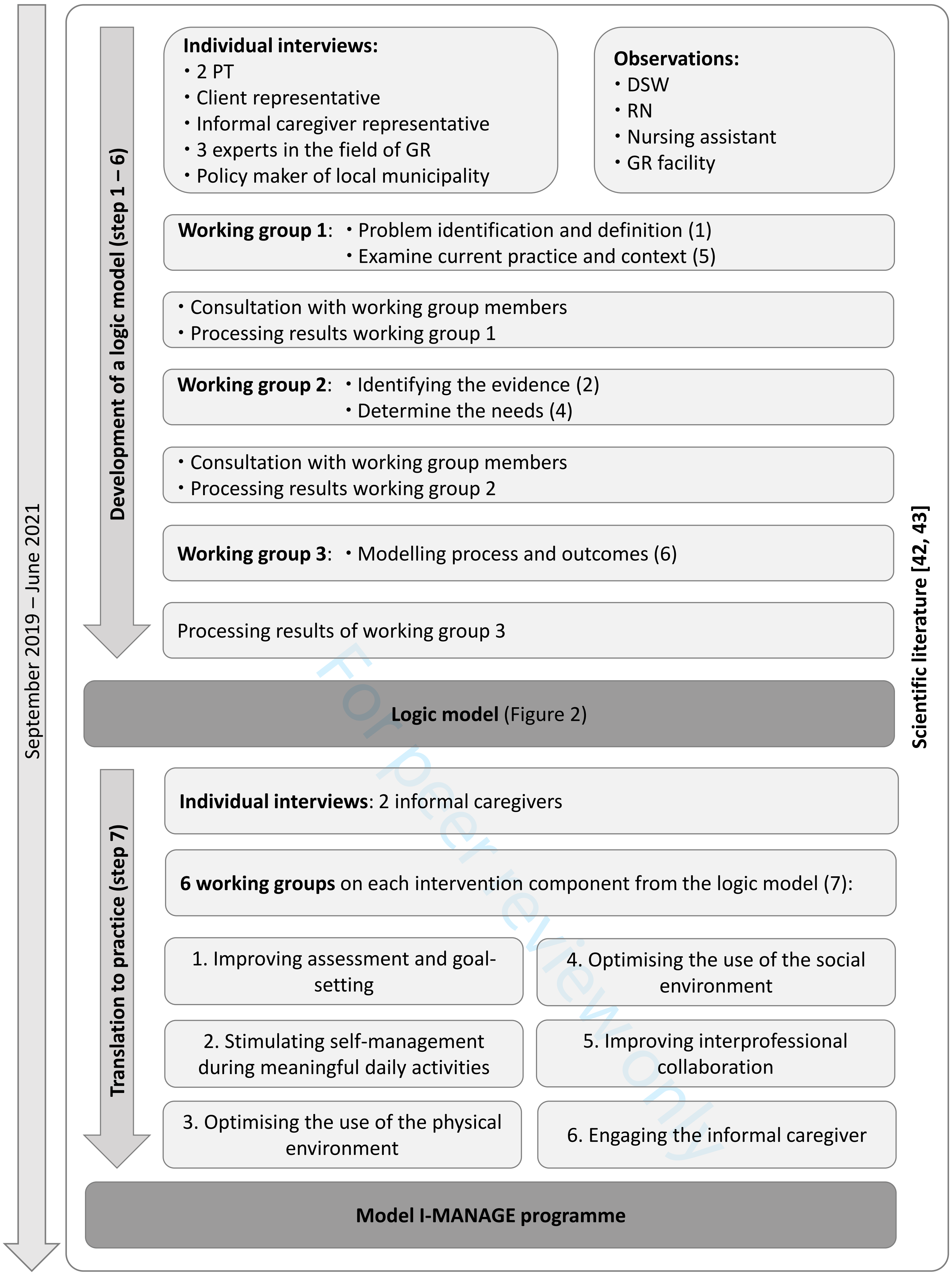

For peer review only

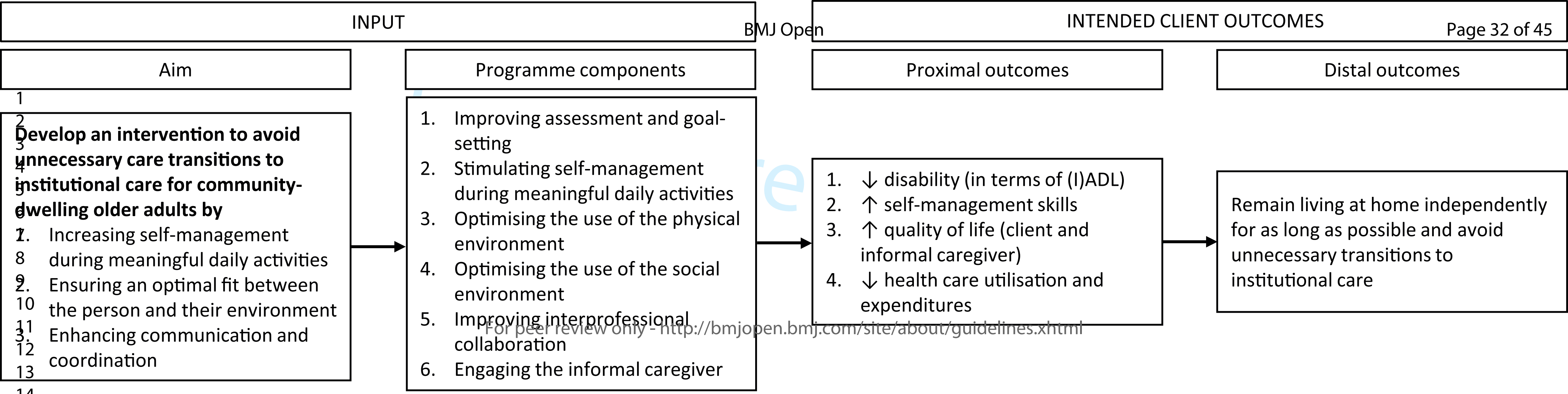

For peer review only - http://bmjopen.bmj.com/site/about/guidelines.xhtml

Referral of the client to I-MANAGE programme:
- Community nurses
- General practitioner
- Institutional care facilities

Start of the programme

Phase 1: Initiation
1. Provide information to client (and informal caregiver)
2. First visit by OT
   - Exploratory conversation based on Positive Health
3. If discharged from other care facility; initiate smooth handover

Week 1

Phase 2: Intake
1. Environmental assessment by OT
2. Set meaningful goals by using COPM
   - Guided by exploratory conversation from phase 1
3. Intake with informal caregiver to assess burden and needs

Week 2

Phase 3: Care plan
1. Determine interventions and actions to reach goals
   - Guided by preferences of client/informal caregiver
   - Specific attention for client's capabilities, social network and physical environment
2. Recorded in electronic care file

Week 3-8

Phase 4: Care delivery
1. Care provided as described in care plan
2. Follow-up on the care process by reablement team
3. If needed, initiate additional support for informal caregiver

Week 8

Phase 5: Evaluation
1. Continuous evaluation during care visit and team meetings
   - Continue, adjust or terminate care plan
2. Formal evaluation after 8 weeks by using COPM

Aftercare
1. Continue programme for maximum of 2 weeks
2. Referral to usual care

Interdisciplinary collaboration:
- Appointed care coordinator
- Regular team meetings
- Shared electronic care file
- Coaching on the job

Supplementary file 1. GUIDED checklist. A guideline for reporting for intervention development studies.

**Item description**	**Explanation**	**Page in manuscript where item is located**	**Other***
Report the context for which the intervention was developed.	Understanding the context in which an intervention was developed informs readers about the suitability and transferability of the intervention to the context in which they are considering evaluating, adapting or using the intervention. Context here can include place, organisational and wider socio- political factors that may influence the development and/or delivery of the intervention.	P9	
Report the purpose of the intervention development process.	Clearly describing the purpose of the intervention specifies what it sets out to achieve. The purpose may be informed by research priorities, for example those identified in systematic reviews, evidence gaps set out in practice guidance such as The National Institute for Health and Care Excellence or specific prioritisation exercises such as those undertaken with patients and practitioners through the James Lind Alliance.	P6-7	
Report the target population for the intervention development process.	The target population is the population that will potentially benefit from the intervention – this may include patients, clinicians, and/or members of the public. If the target population is clearly described then readers will be able to understand the relevance of the intervention to their own research or practice. Health inequalities, gender and ethnicity are features of the target population that may be relevant to intervention development processes.	P9	
Report how any published intervention development approach contributed to the development process.	Many formal intervention development approaches exist and are used to guide the intervention development process (e.g. Squid or The Person Based Approach to Intervention Development). Where a formal intervention development approach is used, it is helpful to describe the process that was followed, including any deviations. More general approaches to intervention development also exist and have been categorised as follows: Target Population-centred intervention development; evidence and theory-based intervention development; partnership intervention development; implementation-based intervention development; efficacy- based intervention development; step or phased-based intervention development; and intervention-specific intervention development. These approaches do not always have specific guidance that describe their use. Nevertheless, it is helpful to give a rich description of how any published approach was operationalised	P8	

For peer review only

Report how evidence from different sources informed the intervention development process.	Intervention development is often based on published evidence and/or primary data that has been collected to inform the intervention development process. It is useful to describe and reference all forms of evidence and data that have informed the development of the intervention because evidence bases can change rapidly, and to explain the manner in which the evidence and/or data was used. Understanding what evidence was and was not available at the time of intervention development can help readers to assess transferability to their current situation.	P10	Supplementary file 3 + [1]
Report how/if published theory informed the intervention development process.	Reporting whether and how theory informed the intervention development process aids the reader's understanding of the theoretical rationale that underpins the intervention. Though not mentioned in the e-Delphi or consensus meeting, it became increasingly apparent through the development of our guidance that this theory item could relate to either existing published theory or programme theory.	P10	Supplementary file 3 + [2]
Report any use of components from existing interventions in the current intervention development process.	Some interventions are developed with components that have been adopted from existing interventions. Clearly identifying components that have been adopted or adapted and acknowledging their original source helps the reader to understand and distinguish between the novel and adopted components of the new intervention.	P10	Supplementary file 3 + [1]
Report any guiding principles, people or factors that were prioritised when making decisions during the intervention development process.	Reporting any guiding principles that governed the development of the application helps the reader to understand the authors' reasoning behind the decisions that were made. These could include the examples of particular populations who views are being considered when designing the intervention, the modality that is viewed as being most appropriate, design features considered important for the target population, or the potential for the intervention to be scaled up.	P9-11	Supplementary file 3
Report how stakeholders contributed to the intervention development process.	Potential stakeholders can include patient and community representatives, local and national policy makers, health care providers and those paying for or commissioning health care. Each of these groups may influence the intervention development process in different ways. Specifying how differing groups of stakeholders contributed to the intervention development process helps the reader to understand how stakeholders were involved and the degree of influence they had on the overall process. Further detail on how to integrate stakeholder contributions within intervention reporting are available.	P9-11	
Report how the intervention	Intervention development is frequently an iterative process. The conclusion of the initial	P13-17	

For peer review only

changed in content and format from the start of the intervention development process.	phase of intervention development does not necessarily mean that all uncertainties have been addressed. It is helpful to list remaining uncertainties such as the intervention intensity, mode of delivery, materials, procedures, or type of location that the intervention is most suitable for. This can guide other researchers to potential future areas of research and practitioners about uncertainties relevant to their healthcare context.		
Report any changes to the intervention required or likely to be required for subgroups.	Specifying any changes that the intervention development team perceive are required for the intervention to be delivered or tailored to specific sub groups enables readers to understand the applicability of the intervention to their target population or context. These changes could include changes to personnel delivering the intervention, to the content of the intervention, or to the mode of delivery of the intervention.	P19-21	
Report important uncertainties at the end of the development process.	Intervention development is frequently an iterative process. The conclusion of the initial phase of intervention development does not necessarily mean that all uncertainties have been addressed. It is helpful to list remaining uncertainties such as the intervention intensity, mode of delivery, materials, procedures, or type of location that the intervention is most suitable for. This can guide other researchers to potential future areas of research and practitioners about uncertainties relevant to their healthcare context.	P19-21	
Follow TIDieR guidance when describing the developed intervention.	Interventions have been poorly reported for a number of years. In response to this, internationally recognized guidance has been published to support the high quality reporting of health care interventions and public health interventions. This guidance should therefore be followed when describing a developed intervention.		
Brief name.	Provide the name or a phrase that describes the intervention.	P1	Title page
Rationale, theory and aim of the elements essential to the intervention.	Describe any rationale, theory, or goal of the elements essential to the intervention.	P13	Supplementary file 3 + [2]
Materials used in the intervention delivery or in the training of intervention providers.	Describe any physical or informational materials used in the intervention, including those provided to participants or used in intervention delivery or in training of intervention providers. Provide information on where the materials can be accessed (e.g. online appendix, URL).	P13-17	
Procedures, activities and/or processes used including enabling	Describe each of the procedures, activities, and/or processes used in the intervention, including any enabling or support activities.	P13-17	

For peer review only

or support activities.			
Expertise and background of intervention providers.	For each category of intervention provider (e.g. psychologist, nursing assistant), describe their expertise, background and any specific training given.	P15-17	
How the intervention was provided.	Describe the modes of delivery (e.g. face-to-face or by some other mechanism, such as internet or telephone) of the intervention and whether it was provided individually or in a group.	P13-17	
Location where the intervention occurred (incl. necessary infrastructure or relevant features).	Describe the type(s) of location(s) where the intervention occurred, including any necessary infrastructure or relevant features.	P9	
When and how much the intervention was delivered.	Describe the number of times the intervention was delivered and over what period of time including the number of sessions, their schedule, and their duration, intensity or dose.	P13-17	
Tailoring – what, why, when and how.	If the intervention was planned to be personalised, titrated or adapted, then describe what, why, when, and how.	P19-20	
Modifications. †	If the intervention was modified during the course of the study, describe the changes (what, why, when, and how).	N/A	
Strategies to improve or maintain fidelity.	If intervention adherence or fidelity was assessed, describe how and by whom, and if any strategies were used to maintain or improve fidelity, describe them.	P19-20	
Adherence and fidelity.†	If intervention adherence or fidelity was assessed, describe the extent to which the intervention was delivered as planned.	N/A	
Report the intervention development process in an open access format.	Unless reports of intervention development are available people considering using an intervention cannot understand the process that was undertaken and make a judgement about its appropriateness to their context. It also limits cumulative learning about intervention development methodology and observed consequences at later evaluation, translation and implementation stages. Reporting intervention development in an open access (Gold or Green) publishing format increases the accessibility and visibility of intervention development research and makes it more likely to be read and used. Potential platforms for open access publication of intervention development include open access journal publications, freely accessible funder reports or a study web-page that details the intervention development process.		Aim of this manuscript

Note. * e.g. if item is reported elsewhere, then the location of this information can be stated here. † If completing the TIDieR checklist for a protocol, these items are not relevant to the protocol and cannot be described until the study is complete.

For peer review only

References

1. Mouchaers I, Van Haastregt JCM, Verbeek H, et al. « Reablement » comme outil thérapeutique pour offrir aux personnes âgées une vie de qualité à domicile après une réadaptation gériatrique. Rev Med Brux. 2021;42(4):324-32.
2. Mouchaers I, Verbeek H, Kempen GIJM, et al. The concept of disability and its causal mechanisms in older people over time from a theoretical perspective: a literature review. Eur J Ageing. 2022 2022/01/29.

For peer review only

Supplementary file 2. Overview of the different stakeholders involved per research activity.

**Stakeholders**[a] (n = 26)	**Observations**[b]	**Individual interviews**	**Working groups**								
			**Development of a logic model**[c]			**Translation to practice**[d]					
			1	2	3	1	2	3	4	5	6
Domestic support worker	•		•	•	•		•			•	
Registered nurse (n = 3)	•		•	•	•	•	•		•	•	
Nursing assistant	•										
Physiotherapist (n = 4)		•	•	•	•	•				•	
Expert in the field of GR research (n = 3)		•	•								
Informal caregiver (n = 3)		•									•
Policy maker of the local municipality (n = 2)		•		•	•			•	•		
Client representative		•	•	•					•		
Informal caregiver representative		•	•	•	•						
Occupational therapist (n = 2)			•	•	•	•	•	•			
Psychologist			•	•	•						•
Geriatrician						•					
Reablement researcher							•				
Manager community care team										•	
Consultant informal care									•		•

GR = geriatric rehabilitation

Note: [a] if n > 1, not all stakeholders participated in every indicated research activity for that group; [b] observations were performed by the researchers; [c] indicates the three consecutive working group sessions throughout which the logic model was being developed following step 1 to 6 as described by Bleijenberg et al. [1]; [d] indicated the 6 working groups related to each programme component described in the logic model (1 = improving assessment and goal-setting, 2 = stimulating self-management during meaningful daily activities, 3 = optimising the use of the physical environment, 4 = optimising the use of the social environment, 5 = improving interprofessional collaboration, and 6 = engaging the informal caregiver).

References

1. Bleijenberg N, de Man-van Ginkel JM, Trappenburg JCA, et al. Increasing value and reducing waste by optimizing the development of complex interventions: Enriching the development phase of the Medical Research Council (MRC) Framework. Int J Nurs Stud. 2018;79:86-93.

For peer review only

Supplementary file 3. Overview of the intermediate results of the first five steps of the development phase.

Programme development step	Results
1. Problem identification	The identified problems were situated in three main areas:<br>1. A lack of attention to meaningful daily activities. According to the working group, older persons were mainly focused on functional goals related to (I)ADL tasks rather than meaningful daily activities that increase their well-being. Additionally, the focus of care is often on eliminating diseases and symptoms instead of maintaining quality of life [1, 2];<br>2. There appeared a gap between the person's abilities, needs, and wishes, and the environment they reside in. When older people experience a deterioration in health status, their environment is often not adapted, i.e. the necessary home modifications and assistive devices are not always in place [3, 4];<br>3. Communication and coordination within the care trajectory of an older person. There is insufficient communication amongst different care professionals but also with older persons and informal caregivers [4-7].
2. Identifying the evidence	The identification of the evidence-based practices was mainly based on literature research. A literature review was performed to gain insight into current evidence on effective reablement programmes aimed at improving the independent living of older persons [8]. We specifically sought to gain a general understanding of different reablement programmes, their characteristics (e.g. duration), content, outcome measures, and effectiveness. Thirteen programmes were identified, all intended for (I)ADL-impaired community-dwelling older adults. The most common features were: 1) delivery by a trained and coordinated multidisciplinary team and 2) implementation of an intake assessment and goal-setting tool. The applied interventions focused on supporting (I)ADL and physical functioning, and improving home safety. Nine programmes showed a statistically significant improvement on at least one of the outcome measures (e.g. (I)ADL functioning).
3. Identifying or developing theory	A literature review was performed on the concept of disability and its working mechanisms [9] to identify a solid theoretical foundation for our programme (*step 3*). We concluded that by optimising the use of personal, as well as environmental, resources and focusing on health and functioning, rather than disability, an older person's independence and wellbeing could be improved, especially while performing meaningful daily activities in accordance with the person's needs and preferences.
4. Determine the needs	The determined needs are closely related to the identified problems. Stakeholders expressed the need for:<br>1. Standardised goal-setting at the beginning of the care process. Goals should be meaningful, embedded in the older person's own environment, and not solely focused on (I)ADL;<br>2. Timely assessment and, if necessary, adaptations to the home environment of the older person. This concerns adaptations to the built environment, as well as the availability of assistive devices. Additionally, the social environment of the older person needs to be assessed in order to make optimal use of it or provide additional support if the social network is rather weak;<br>3. Improved communication and coordination within the care trajectory of the older person. For example, a smooth handover of information between care professionals and organisations, involvement of the informal caregiver when discussing a client's care needs, multidisciplinary collaboration with clear roles for each provider, and the appointment of a care coordinator.
5. Examining current practice and context	When examining the current practice and context we focused on positive aspects and possible barriers for implementation. Possible barriers could be different financial arrangements and different communication systems within a multidisciplinary team. Positive aspects include the comprehensive assessment by community nurses at the start

For peer review only

	of receiving community care, a signalling role for domestic support workers, and the presence of various welfare initiatives in the Netherlands. A suggestion was made to include the principles of Positive Health [10] in the programme, a best practice that is already known and applied by some care professionals.

References

1. Nicholson C, Meyer J, Flatley M, et al. The experience of living at home with frailty in old age: A psychosocial qualitative study. Int J Nurs Stud. 2013 2013/09/01/;50(9):1172-9.
2. Aspinal F, Glasby J, Rostgaard T, et al. New horizons: Reablement - supporting older people towards independence. Age Ageing. 2016;45(5):572-6.
3. Q-Consult zorg. Inventarisatie zorginhoud GRZ: Het net opgehaald na zes jaar GRZ in de Zorgverzekeringswet. Q-Consult zorg, 2019.
4. Rustad EC, Furnes B, Cronfalk BS, et al. Older patients' experiences during care transition. Patient Prefer Adherence. 2016;10:769-79.
5. Everink IHJ, van Haastregt JCM, Kempen GIJM, et al. Uitdagingen in de geriatrische revalidatiezorg: de ontwikkeling van een zorgpad. Tijdschr Gerontol Geriatr. 2015 2015/04/01;46(2):104-12.
6. Bauer M, Fitzgerald L, Haesler E, et al. Hospital discharge planning for frail older people and their family. Are we delivering best practice? A review of the evidence. J Clin Nurs. 2009;18(18):2539-46.
7. Adelman RD, Tmanova LL, Delgado D, et al. Caregiver burden: a clinical review. JAMA. 2014;311(10):1052-60.
8. Mouchaers I, Van Haastregt JCM, Verbeek H, et al. « Reablement » comme outil thérapeutique pour offrir aux personnes âgées une vie de qualité à domicile après une réadaptation gériatrique. Rev Med Brux. 2021;42(4):324-32.
9. Mouchaers I, Verbeek H, Kempen GIJM, et al. The concept of disability and its causal mechanisms in older people over time from a theoretical perspective: a literature review. Eur J Ageing. 2022 2022/01/29.
10. Huber M, van Vliet M, Giezenberg M, et al. Towards a ‘patient-centred’ operationalisation of the new dynamic concept of health: a mixed methods study. BMJ Open. 2016;6(1):e010091.

For peer review only

Supplementary file 1. GUIDED checklist. A guideline for reporting for intervention development studies.

Item description	Explanation	Page in manuscript where item is located	Other*
Report the context for which the intervention was developed.	Understanding the context in which an intervention was developed informs readers about the suitability and transferability of the intervention to the context in which they are considering evaluating, adapting or using the intervention. Context here can include place, organisational and wider socio- political factors that may influence the development and/or delivery of the intervention.	P9	
Report the purpose of the intervention development process.	Clearly describing the purpose of the intervention specifies what it sets out to achieve. The purpose may be informed by research priorities, for example those identified in systematic reviews, evidence gaps set out in practice guidance such as The National Institute for Health and Care Excellence or specific prioritisation exercises such as those undertaken with patients and practitioners through the James Lind Alliance.	P6-7	
Report the target population for the intervention development process.	The target population is the population that will potentially benefit from the intervention – this may include patients, clinicians, and/or members of the public. If the target population is clearly described then readers will be able to understand the relevance of the intervention to their own research or practice. Health inequalities, gender and ethnicity are features of the target population that may be relevant to intervention development processes.	P9	
Report how any published intervention development approach contributed to the development process.	Many formal intervention development approaches exist and are used to guide the intervention development process (e.g. Squid or The Person Based Approach to Intervention Development). Where a formal intervention development approach is used, it is helpful to describe the process that was followed, including any deviations. More general approaches to intervention development also exist and have been categorised as follows: Target Population-centred intervention development; evidence and theory-based intervention development; partnership intervention development; implementation-based intervention development; efficacy- based intervention development; step or phased-based intervention development; and intervention-specific intervention development. These approaches do not always have specific guidance that describe their use. Nevertheless, it is helpful to give a rich description of how any published approach was operationalised	P8	

For peer review only

Report how evidence from different sources informed the intervention development process.	Intervention development is often based on published evidence and/or primary data that has been collected to inform the intervention development process. It is useful to describe and reference all forms of evidence and data that have informed the development of the intervention because evidence bases can change rapidly, and to explain the manner in which the evidence and/or data was used. Understanding what evidence was and was not available at the time of intervention development can help readers to assess transferability to their current situation.	P10	Supplementary file 3 + [1]
Report how/if published theory informed the intervention development process.	Reporting whether and how theory informed the intervention development process aids the reader's understanding of the theoretical rationale that underpins the intervention. Though not mentioned in the e-Delphi or consensus meeting, it became increasingly apparent through the development of our guidance that this theory item could relate to either existing published theory or programme theory.	P10	Supplementary file 3 + [2]
Report any use of components from existing interventions in the current intervention development process.	Some interventions are developed with components that have been adopted from existing interventions. Clearly identifying components that have been adopted or adapted and acknowledging their original source helps the reader to understand and distinguish between the novel and adopted components of the new intervention.	P10	Supplementary file 3 + [1]
Report any guiding principles, people or factors that were prioritised when making decisions during the intervention development process.	Reporting any guiding principles that governed the development of the application helps the reader to understand the authors' reasoning behind the decisions that were made. These could include the examples of particular populations who views are being considered when designing the intervention, the modality that is viewed as being most appropriate, design features considered important for the target population, or the potential for the intervention to be scaled up.	P9-11	Supplementary file 3
Report how stakeholders contributed to the intervention development process.	Potential stakeholders can include patient and community representatives, local and national policy makers, health care providers and those paying for or commissioning health care. Each of these groups may influence the intervention development process in different ways. Specifying how differing groups of stakeholders contributed to the intervention development process helps the reader to understand how stakeholders were involved and the degree of influence they had on the overall process. Further detail on how to integrate stakeholder contributions within intervention reporting are available.	P9-11	
Report how the intervention	Intervention development is frequently an iterative process. The conclusion of the initial	P13-17	

For peer review only

changed in content and format from the start of the intervention development process.	phase of intervention development does not necessarily mean that all uncertainties have been addressed. It is helpful to list remaining uncertainties such as the intervention intensity, mode of delivery, materials, procedures, or type of location that the intervention is most suitable for. This can guide other researchers to potential future areas of research and practitioners about uncertainties relevant to their healthcare context.		
Report any changes to the intervention required or likely to be required for subgroups.	Specifying any changes that the intervention development team perceive are required for the intervention to be delivered or tailored to specific sub groups enables readers to understand the applicability of the intervention to their target population or context. These changes could include changes to personnel delivering the intervention, to the content of the intervention, or to the mode of delivery of the intervention.	P19-21	
Report important uncertainties at the end of the development process.	Intervention development is frequently an iterative process. The conclusion of the initial phase of intervention development does not necessarily mean that all uncertainties have been addressed. It is helpful to list remaining uncertainties such as the intervention intensity, mode of delivery, materials, procedures, or type of location that the intervention is most suitable for. This can guide other researchers to potential future areas of research and practitioners about uncertainties relevant to their healthcare context.	P19-21	
Follow TIDieR guidance when describing the developed intervention.	Interventions have been poorly reported for a number of years. In response to this, internationally recognized guidance has been published to support the high quality reporting of health care interventions and public health interventions. This guidance should therefore be followed when describing a developed intervention.		
Brief name.	Provide the name or a phrase that describes the intervention.	P1	Title page
Rationale, theory and aim of the elements essential to the intervention.	Describe any rationale, theory, or goal of the elements essential to the intervention.	P13	Supplementary file 3 + [2]
Materials used in the intervention delivery or in the training of intervention providers.	Describe any physical or informational materials used in the intervention, including those provided to participants or used in intervention delivery or in training of intervention providers. Provide information on where the materials can be accessed (e.g. online appendix, URL).	P13-17	
Procedures, activities and/or processes used including enabling	Describe each of the procedures, activities, and/or processes used in the intervention, including any enabling or support activities.	P13-17	

For peer review only

or support activities.			
Expertise and background of intervention providers.	For each category of intervention provider (e.g. psychologist, nursing assistant), describe their expertise, background and any specific training given.	P15-17	
How the intervention was provided.	Describe the modes of delivery (e.g. face-to-face or by some other mechanism, such as internet or telephone) of the intervention and whether it was provided individually or in a group.	P13-17	
Location where the intervention occurred (incl. necessary infrastructure or relevant features).	Describe the type(s) of location(s) where the intervention occurred, including any necessary infrastructure or relevant features.	P9	
When and how much the intervention was delivered.	Describe the number of times the intervention was delivered and over what period of time including the number of sessions, their schedule, and their duration, intensity or dose.	P13-17	
Tailoring – what, why, when and how.	If the intervention was planned to be personalised, titrated or adapted, then describe what, why, when, and how.	P19-20	
Modifications. †	If the intervention was modified during the course of the study, describe the changes (what, why, when, and how).	N/A	
Strategies to improve or maintain fidelity.	If intervention adherence or fidelity was assessed, describe how and by whom, and if any strategies were used to maintain or improve fidelity, describe them.	P19-20	
Adherence and fidelity.†	If intervention adherence or fidelity was assessed, describe the extent to which the intervention was delivered as planned.	N/A	
Report the intervention development process in an open access format.	Unless reports of intervention development are available people considering using an intervention cannot understand the process that was undertaken and make a judgement about its appropriateness to their context. It also limits cumulative learning about intervention development methodology and observed consequences at later evaluation, translation and implementation stages. Reporting intervention development in an open access (Gold or Green) publishing format increases the accessibility and visibility of intervention development research and makes it more likely to be read and used. Potential platforms for open access publication of intervention development include open access journal publications, freely accessible funder reports or a study web-page that details the intervention development process.		Aim of this manuscript

Note. * e.g. if item is reported elsewhere, then the location of this information can be stated here. † If completing the TIDieR checklist for a protocol, these items are not relevant to the protocol and cannot be described until the study is complete.

For peer review only

References

1. Mouchaers I, Van Haastregt JCM, Verbeek H, et al. « Reablement » comme outil thérapeutique pour offrir aux personnes âgées une vie de qualité à domicile après une réadaptation gériatrique. Rev Med Brux. 2021;42(4):324-32.
2. Mouchaers I, Verbeek H, Kempen GIJM, et al. The concept of disability and its causal mechanisms in older people over time from a theoretical perspective: a literature review. Eur J Ageing. 2022 2022/01/29.

For peer review only

BMJ Open

Development and content of a community-based reablement programme (I-MANAGE): A co-creation study

Journal:	*BMJ Open*
Manuscript ID	bmjopen-2022-070890.R2
Article Type:	Original research
Date Submitted by the Author:	09-May-2023
Complete List of Authors:	Mouchaers, Ines; Maastricht University, Department of Health Services Research; Living Lab of Ageing and Long Term Care<br>Verbeek, Hilde ; Maastricht University, Department of Health Services Research; Living Lab of Ageing and Long Term Care<br>Kempen, Gertrudis; Maastricht University, Department of Health Services Research; Living Lab of Ageing and Long Term Care<br>van Haastregt, Jolanda; Maastricht University, Department of Health Services Research; Living Lab of Ageing and Long Term Care<br>Vlaeyen, Ellen; KU Leuven, Public Health and Primary Care; UHasselt, Faculty of Medicine and Life Sciences<br>Goderis, Geert; KU Leuven, Department of Public Health and Primary Care<br>Metzelthin, Silke; Maastricht University, Department of Health Services Research; Living Lab of Ageing and Long Term Care
**Primary Subject Heading**:	Health services research
Secondary Subject Heading:	Patient-centred medicine, Rehabilitation medicine
Keywords:	Health Services for the Aged, Patient-Centered Care, Primary Health Care, Quality of Life, Rehabilitation medicine < INTERNAL MEDICINE

SCHOLARONE™
Manuscripts

I, the Submitting Author has the right to grant and does grant on behalf of all authors of the Work (as defined in the below author licence), an exclusive licence and/or a non-exclusive licence for contributions from authors who are: i) UK Crown employees; ii) where BMJ has agreed a CC-BY licence shall apply, and/or iii) in accordance with the terms applicable for US Federal Government officers or employees acting as part of their official duties; on a worldwide, perpetual, irrevocable, royalty-free basis to BMJ Publishing Group Ltd ("BMJ") its licensees and where the relevant Journal is co-owned by BMJ to the co-owners of the Journal, to publish the Work in this journal and any other BMJ products and to exploit all rights, as set out in our licence.

The Submitting Author accepts and understands that any supply made under these terms is made by BMJ to the Submitting Author unless you are acting as an employee on behalf of your employer or a postgraduate student of an affiliated institution which is paying any applicable article publishing charge ("APC") for Open Access articles. Where the Submitting Author wishes to make the Work available on an Open Access basis (and intends to pay the relevant APC), the terms of reuse of such Open Access shall be governed by a Creative Commons licence – details of these licences and which Creative Commons licence will apply to this Work are set out in our licence referred to above.

Other than as permitted in any relevant BMJ Author's Self Archiving Policies, I confirm this Work has not been accepted for publication elsewhere, is not being considered for publication elsewhere and does not duplicate material already published. I confirm all authors consent to publication of this Work and authorise the granting of this licence.

For peer review only

TITLE PAGE

Title

Development and content of a community-based reablement programme (I-MANAGE): A co-creation study

Authors

Ines Mouchaers, MSc, [1, 2, 3 *]

Hilde Verbeek, PhD, [1, 2]

Gertrudis I.J.M. Kempen, PhD, [1, 2]

Jolanda C.M. van Haastregt, PhD, [1, 2]

Ellen Vlaeyen, PhD, [3, 5, 6]

Geert Goderis, PhD, [4]

Silke F. Metzelthin, PhD, [1, 2]

Author Affiliations

[1] Department of Health Services Research, Faculty of Health Medicine and Life Sciences, CAPHRI Care and Public Health Research Institute, Maastricht University, Maastricht, the Netherlands

[2] Living Lab of Ageing and Long Term Care, Maastricht, the Netherlands

[3] Department of Public Health and Primary Care, Academic Centre for Nursing and Midwifery, KU Leuven, Leuven, Belgium

[4] Department of Public Health and Primary Care, Academic Centre for General Practice, KU Leuven, Leuven, Belgium

[5] Faculty of Medicine and Life Sciences, Hasselt University, Hasselt, Belgium

[6] University Centre for Nursing and Midwifery, Department of Public Health and Primary Care, Faculty of Medicine and Health Sciences, Ghent University, Belgium

For peer review only

For peer review only - http://bmjopen.bmj.com/site/about/guidelines.xhtml

Corresponding author

Ines Mouchaers: https://orcid.org/0000-0001-7189-4468

* Address correspondence to: Ines Mouchaers, MSc, Department of Health Services Research, P.O. Box 616, 6200 MD Maastricht, The Netherlands.

E-mail: i.mouchaers@maastrichtuniversity.nl; Telephone number: +31-(0)43 388 28 15.

Word count

5086

For peer review only

For peer review only - http://bmjopen.bmj.com/site/about/guidelines.xhtml

ABSTRACT

Objectives: As age increases, people generally start experiencing problems related to independent living, resulting in an increased need for long-term care services. Investing in sustainable solutions to promote independent living is therefore essential. Subsequently, reablement is a concept attracting growing interest. Reablement is a person-centred, holistic approach promoting older adults' active participation through daily, social, leisure, and physical activities. The aim of this paper is to describe the development and content of I-MANAGE, a model for a reablement programme for community-dwelling older adults.

Design: The development of the programme was performed according to the Medical Research Council framework as part of the TRANS-SENIOR international training and research network. A co-creation design was used, including literature research, observations, interviews, and working group sessions with stakeholders.

Setting and participants: The interviews and working group sessions took place in the Dutch long-term home care context. Stakeholders invited to the individual interviews and working group sessions included care professionals, policymakers, client representatives, informal caregiver representatives, informal caregivers, and scientific experts.

Results: The co-creation process resulted in a 5-phase interdisciplinary primary care programme, called I-MANAGE. The programme focuses on improving the self-management and wellbeing of older adults by working towards their meaningful goals. During the programme, the person's physical and social environment will be put to optimal use, and sufficient support will be provided to informal caregivers to reduce their burden. Lastly, the programme aims for continuity of care and better communication and coordination.

Conclusion: The I-MANAGE programme can be tailored to the local practices and resources and is therefore suitable for the use in different settings, nationally and internationally. If the programme is implemented as described, it is important to closely monitor the process and results.

For peer review only - http://bmjopen.bmj.com/site/about/guidelines.xhtml

STRENGTHS AND LIMITATIONS OF THIS STUDY

- Intervention development was conducted using a systematic approach based on the Medical Research Council framework.
- The intervention and its development is theory and evidence based as a result of extensive literature research.
- All end users were represented during the development process, increasing its acceptability and feasibility.
- Data triangulation was used during the development process, increasing validity of the results.
- Client and informal caregiver representatives were included in the working group instead of clients and informal caregivers themselves, possibly missing an important voice.

For peer review only

BACKGROUND

The amount of older adults experiencing disabilities will increase over time and, while a large proportion of the older population remains independent, others will experience an increased need for support [1]. Moreover, 50% of people aged 85 years or older require care and/or support with daily activities [2]. As a result, it is expected that their demands for long-term care services will increase. When older adults live in an environment that is unsafe and does not meet their needs, the challenges they might face regarding independent living will increase further [3]. Previous research showed that a maladjusted environment negatively affects disability, which could lead to an accumulation of health risks, loss of independence, poor quality of life (QoL), and depression [4-6]. Care and service delivery in the community is often fragmented, with little coordination and poor communication amongst care providers, clients, and informal caregivers [3, 7-9]. Furthermore, the focus of care is often on eliminating specific diseases and symptoms instead of supporting the remaining capacity to maintain QoL and independent living [10, 11]. Rather than performing tasks *with* their clients, care professionals often tend to take over [11]. Failing to properly tackle these challenges could increase the use of health and social care and related costs [12]. In addition, this could lead to unnecessary (re)hospitalisations or permanent nursing home placement, which each have their own risks (e.g. increased mortality) [13] and at a time when financial and workforce resources are shrinking [7, 12, 14]. It is therefore essential to invest in sustainable solutions to promote independent living [15-17].

A concept attracting growing interest in promoting independent living among older adults is reablement. This is a person-centred, holistic approach promoting active participation of older adults in daily activities through social, leisure, and physical activities chosen by the older adult in line with their preferences, either at home or in the community [18]. Instead of creating dependency by taking over tasks, care professionals identify the capabilities and opportunities of individuals to maximise their independence by supporting them to achieve their goals, through participation in daily activities, home modifications, assistive devices, and involvement of their social network [11, 18-20]. Current evidence on the effectiveness of reablement interventions is inconclusive [11], however several

For peer review only

systematic reviews have indicated the positive results of reablement relating to activities of daily living (ADL) functioning and health-related QoL [21-23]. Due to the promising results, interest in implementing reablement into everyday care is growing internationally. In Denmark, New Zealand and the United Kingdom, reablement has more-or-less been successfully implemented across the whole country [1]. For example, in Denmark reablement in long-term care for older adults was legally introduced in 2015, meaning that all municipalities must offer reablement interventions and all applicants for home care are assessed for potential for reablement before being offered conventional home care [2].

Despite the promising results and successful implementation abroad, contextual differences mean this is no indication that it would necessarily be effective in its current format in the Dutch home care setting [24]. Implementing reablement is a complex process and influenced by multiple factors, such as organisational factors, individual and social attitudes towards a new form of care, technological factors related to communication, and financial factors [25, 26]. It is crucial to critically consider these factors in the design, delivery, and evaluation of reablement [27]. Moreover, to implement reablement in the Dutch home care setting, existing programmes need to be revised and adapted to suit the current context, which is crucial when developing and delivering complex health care interventions [27]. However, the development and content of community care programmes, such as reablement programmes, are often insufficiently described in the scientific literature [19, 28-30]. There are only limited articles available that describe either the development of the programme or its content in detail [31-33] and these descriptions are often included as part of a feasibility or pilot study [29]. This offers little guidance to replicate or build on the previous findings of such programmes, despite this being essential for the development of new and implementation of existing programmes in different settings [29, 30, 34].

This paper therefore describes the development, using a co-creation process, and content of I-MANAGE, a reablement programme for community-dwelling older adults to improve older adults' self-management and participation in daily life, while also increasing QoL and decreasing informal

For peer review only - http://bmjopen.bmj.com/site/about/guidelines.xhtml

For peer review only

caregivers' burden. By describing the development and content of the programme in detail, we increase the replicability and prevent other researchers from reinventing the wheel. The programme is specifically suitable for the Dutch community care setting, however, due to its extensive description, this manuscript may also provide a model for implementation in other countries.

For peer review only

For peer review only - http://bmjopen.bmj.com/site/about/guidelines.xhtml

METHODS

To describe the development process of the programme and ensure completeness of reporting, we used the guidance for reporting for intervention development studies in health research (GUIDED) (Supplementary file 1) [29]. This checklist provides a clear and structured basis for the reporting of programme development, as well as the description of the content of the programme.

Design

I-MANAGE was developed between September 2019 and June 2021 using a co-creation design. The programme was developed following the first phase (development) of the Medical Research Council (MRC) framework for the development and evaluation of complex interventions [35, 36].

Patient and public involvement

Co-creation was initiated by the researchers as a response to the challenges that are caused by an ageing society (e.g., increasing care needs, decreasing staff). To deal with these challenges the Dutch government stimulates an ageing in place policy and promotes another way of (home) care delivery moving from “doing for…” towards “doing with…” clients, or in the best case to enable clients to do things by themselves again. These developments are also adopted by Dutch care organisations, which hope to improve the quality and sustainability of their care services [37]. All end users (care professionals who would be implementing and delivering the programme, as well as the target population) were represented and involved during the development process of the programme as members of the working groups and by participating in the individual interviews. However, they were not involved in the development of the study design of dissemination of the findings.

Setting and participants

I-MANAGE is based on international evidence and tailored to the Dutch home care context. Home care in the Netherlands includes personal care (i.e. assistance with ADL), nursing care (i.e. medical

For peer review only - http://bmjopen.bmj.com/site/about/guidelines.xhtml

assistance), and domestic support (i.e. assistance with instrumental activities of daily living (IADL)) [38]. Usually home care is funded by two statutory forms of insurance cover care: the *Health Insurance Act* (ZVW) and the *Social Support Act* (WMO). Clients often use a combination of ZVW (e.g. general practitioner care, therapists, hospital care, or medication), and WMO (e.g. domestic support, home adaptations) [38, 39]. The programme was developed for community-dwelling older adults, irrespective of age or cognitive and functional status.

The programme was developed in co-creation with stakeholders who participated in observations, individual interviews and working groups. Supplementary file 2 provides an overview of all stakeholders involved and the research activities they participated in. Participants were recruited from the professional network of the researchers. They were informed about the study and asked to participate via email. When participants agreed to participate, verbal or written informed consent was provided before the start of each interview or working group session.

Data collection

First, a logic model was created. A logic model is a tool to illustrate how a programme will create change [40]. The logic model systematically visualises the aim and sub-aims of the programme, the programme components, and the intended outcomes [40]. The logic model was developed using six iterative programme development steps: 1) identifying the problem, 2) identifying the evidence, 3) identifying or developing a theory, 4) determining needs, 5) examining current practices and the context, and 6) modelling processes and outcomes [41]. The logic model was then translated to practice (step 7). Data collection was performed following a non-linear and iterative process as described by Bleijenberg et al. [41], and by using a variety of data collection methods, including literature research, observations, individual interviews, and working groups. Figure 1 illustrates the development process in detail.

[Insert Figure 1 here]

For peer review only - http://bmjopen.bmj.com/site/about/guidelines.xhtml

Development of a logic model (steps 1 to 6)

All steps were guided by literature research, especially steps 2 and 3 of the development process. To gain insight into current evidence-based practices (*step 2*), the relevant scientific literature on reablement programmes was reviewed. The methodology and results of this literature research is described elsewhere [42]. To identify a theoretical foundation for the programme (*step 3*), a literature review was performed on the concept of disability and its underlying causal mechanisms [43].

Furthermore, a working group was composed and invited to three sessions (1.5 h each). Members of the working group included physiotherapists (PT), occupation therapists (OT), registered nurses (RN), a domestic support worker (DSW), a policymaker of the local municipality, a psychologist, a client representative, informal caregiver representatives, and a geriatric rehabilitation expert. The first session focused on the identification of the problem (*step 1*) and the examination of the current practice and context (*step 5*). Session two focused on the previously identified evidence, which participants could complement with practices from their own field of work (*step 2*), and the determination of needs (*step 4*), closely related to the previously identified problems. The third session focused on modelling the process and outcomes (*step 6*), during which a preliminary logic model was presented, on which participants could provide feedback. Between sessions, working group members were consulted for additional input and clarification if needed. The researchers processed the results from each session and the additional information in order to be used as a starting point for the next session. The working group sessions were led by the first and last author (IM and SM).

We then interviewed two PTs, a policy maker of the local municipality, a client and informal caregiver representative and three experts in the field of geriatric rehabilitation research. In total, eight interviews were conducted, all focusing on identifying the problem (*step 1*), determining the needs (*step 4*), and gaining insight into current practice and context (*step 5*). In addition, we interviewed two informal caregivers. Topics addressed during these interviews included their role as

For peer review only - http://bmjopen.bmj.com/site/about/guidelines.xhtml

informal caregiver, how involved they were in the care process, how they were or felt supported and by whom and what they would like to change in the process.

Lastly, observations were performed by the researchers. Observations were conducted in the traditional community care setting to examine current practice and context of home care services (*step 5*) and to identify problems (*step 1*). Six observations, each lasting half a day, were conducted by shadowing a DSW, an RN, and a nursing assistant, and three days were spent with allied health professionals. Field notes were taken throughout the observation periods.

Translation to practice (step 7)

For each of the six programme components in the logic model, we invited members of the working group, a geriatrician, informal care consultant, reablement researcher, informal caregiver, and a community care teams' manager to a session to translate components into practice (*step 7*). Based on the programme component, the most relevant stakeholders were invited. For example, on informal caregiver support, we invited an informal care consultant, an informal caregiver, and a psychologist; resulting in three to four members per working group. Each working group attended one session lasting 1 hour. Each session began with an introduction to the goal of the programme, the logic model, and the results of steps 1 to 6 related to the programme component. Afterwards, participants were asked open-ended questions about the practical implementation as well as barriers and facilitators of the component. Lastly, results were summarised and participants were asked for final feedback. Table 1 provides an overview of the different methodologies used in each development step.

Table 1. Overview of the different methodologies used in each development step defined by Bleijenberg et al. [41]

**Programme development steps**	**Methodology**			
	**Literature research**	**Observations**	**Individual interviews**	**Working groups**
1. Problem identification	○	●	●	●
2. Identifying the evidence	●			●
3. Identifying or developing theory	●			
4. Determine the needs	○		●	●
5. Examining current practice and context	○	●	●	●
6. Modelling process and outcomes	○	○	○	●

For peer review only - http://bmjopen.bmj.com/site/about/guidelines.xhtml

7. Translation to practice	●	●

Note: The full black dots ● indicate a main source of information for the particular step, while the full grey dots ● indicate only a minor influence and guidance for the particular step.

Data analysis

We used data triangulation to verify the results. Our main source of information was the working group sessions, as they provided the richest data on the perspectives of the different target groups. Individual interviews, observations, and literature research were used to complement and check the information obtained throughout the working group sessions. Working group sessions and individual interviews were recorded and transcribed non-verbatim. A thematic analysis was conducted based on the steps described by Braun and Clarke [44]. Common themes were identified within each step of the development process (e.g. lack of sufficient communication). Afterwards, the themes from all sessions were compared and associations were found between, for example, identified problems and determined needs, or current scientific evidence and best practices of the organisation. The results of each session provided input and structure for the next session. After each working group session or individual interview, a member check was conducted by summarising the most important findings. The results were discussed regularly within the research team. The research team consists of all authors involved. They work in the field of social gerontology, public health and primary and long-term care. A research logbook was kept by the researchers to keep track of all research activities and intermediate results, together with field notes from these activities.

For peer review only

For peer review only - http://bmjopen.bmj.com/site/about/guidelines.xhtml

RESULTS

The following section presents the final model of the I-MANAGE programme in detail, starting with the developed logic model, and eventually the translation to practice, which contains a detailed description of all programme components. A detailed description of the results from the first five programme development steps is provided in Supplementary file 3.

Development of a logic model

The logic model starts with the aim and sub-aims of the programme based on identified problems and needs (*result from development steps 1 and 4*), which are derived mostly from the working group sessions and individual interviews. To fulfil these aims, six programme components were determined: 1) improving assessment and goal-setting; 2) stimulating self-management during meaningful daily activities; 3) optimising the use of the physical environment; 4) optimising the use of the social environment; 5) improving interprofessional collaboration; and 6) supporting the informal caregiver. These originate from both the input from stakeholders (*result from development steps 2, 4, 5 and 6)* and evidence-based practices from the literature (*result from development step 2*). The intended client outcomes of the programme are reducing (I)ADL disability, improving self-management skills, increasing QoL of both the client and informal caregiver and reducing healthcare utilisation and expenditures (proximal outcomes), which are common outcome measures in reablement programmes abroad. Eventually, improving these proximal outcomes would help the older adults to remain living at home independently and avoid unnecessary transitions to institutional care (distal outcomes). Figure 2 presents the logic model.

[Insert Figure 2 here]

Translation to practice: description of the I-MANAGE programme

The I-MANAGE programme is the result of input from stakeholders during the last step of the development process (*step 7*) [41]. The following sections describe the programme in detail. The

For peer review only - http://bmjopen.bmj.com/site/about/guidelines.xhtml

programme, as described, may serve as a model for the use in different local settings and leaves room for tailoring to the specific needs and resources of the organisation.

To achieve the intended outcomes presented in the logic model, the programme intends to apply a two-tiered approach: 1) focusing on the capabilities of the client and 2) paying attention to environmental factors (i.e. physical, social, and organisational). I-MANAGE is intended for community-dwelling older adults who are still able to (re)learn new skills and apply them in daily life without an indication of terminal illness or planned nursing home admission. The average duration of the programme should be 8 weeks according to the members of the working groups.

Interdisciplinary collaboration

I-MANAGE aims to facilitate interdisciplinary collaboration by encouraging intensive collaboration between the reablement team, client, informal caregiver and other care professionals delivering care and support to the client and informal caregiver. The programme should be delivered by an interdisciplinary reablement team, generally consisting of an RN, an OT, and, depending on the local context, supplemented with a PT, social worker, DSW, or other disciplines. The reablement team initiates the programme and is responsible for the direct support of the client and informal caregiver. A care coordinator should be appointed, which in most cases, is the OT. Depending on the necessary care and goals set by the client, the reablement team may consult other care professionals (e.g. usual home care team, psychologist, or general practitioner); for example, during the intake phase for advice or while working on the client's goals. They should be informed and coached by the reablement team.

Furthermore, (bi-)weekly team meetings should be organised to discuss the intake of new clients and informal caregivers, the progress made by clients, and the final evaluation of clients' personal goals. Additionally, team meetings provide the opportunity to discuss specific cases with other team members and to organise training. All members of the reablement team should have access to the client's electronic care file (ECF) to access the care plan, report progress, and follow up on each other's work. Lastly, when discharged from another care facility (e.g. hospital or rehabilitation

For peer review only - http://bmjopen.bmj.com/site/about/guidelines.xhtml

facility), a smooth handover of client information should be initiated so no necessary details of the client's care process are lost.

Practice-oriented training

Preceding the programme, the reablement team should receive practice-oriented training focusing on adequately delivering I-MANAGE. Within this training, care professionals receive a detailed manual beforehand, consisting of background information and a description of the programme, including the goal, key components, and I-MANAGE's care process. The manual was developed by the researchers and revised by working group members to make sure it is suitable for practice. Additionally, a toolkit for achieving individual client goals is provided (e.g. exercise booklet based on the OTAGO exercise programme [45], social map including welfare initiatives, and tools to assess needs and wishes of the caregiver). The training consists of multiple sessions. The first is a kick-off meeting for the reablement team, focusing on the goal and content of I-MANAGE. Secondly, specific training sessions occur for OT and PT concerning the use of the Canadian Occupational Performance Measure (COPM) [46] to set personal goals with the client and the OTAGO exercise booklet respectively. Finally, the reablement team receives a booster session to practice and discuss challenging situations and focus on motivational and conversation skills. The kick-off and booster sessions each last two hours and the specific sessions for OT and PT last one hour. Additionally, the programme stimulates training on the job, meaning that members of the reablement team can coach each other and external care professionals based on their own expertise.

I-MANAGE care process

The I-MANAGE care process consists of five consecutive phases: 1) initiation; 2) intake; 3) care plan; 4) care delivery; and 5) evaluation. Each phase is described in detail below and presented in Figure 3. The five phases are a result of the practical translation of the previously described logic model.

For peer review only - http://bmjopen.bmj.com/site/about/guidelines.xhtml

Phase 1: Initiation

Programme referrals will be done through community nurses, general practitioners, or institutional care facilities. Community-dwelling older adults eligible for I-MANAGE should receive information about the programme. The care coordinator has to plan a first visit to present the programme to the client and, if applicable, their informal caregiver to provide the opportunity to ask additional questions. The care coordinator should initiate an exploratory conversation based on the principles of positive health to gain insight into the client's needs and wishes [47].

Phase 2: Intake

Within the first week after the initiation phase, the OT should perform an environmental assessment, identifying necessary home modifications and assistive devices to ensure a safe environment. The environmental assessment is not limited to the inside environment but does also include the entrance and outside environment. Additionally, the care coordinator, or an assigned social worker, should perform an intake with the informal caregiver assessing their needs and wishes. Furthermore, the OT must set meaningful goals with the client using COPM [46] (maximum of five goals). This instrument requires the clients to score both their performance and satisfaction when performing these activities [46]. Goal setting is guided by the exploratory conversation held in phase 1. The ultimate goal is to improve the client's participation and wellbeing; therefore, goals should not merely be (I)ADL-related (e.g. meeting friends at the local café, or painting in the hobby room on the first floor). Goals should be recorded in the client's ECF.

Phase 3: Care plan

Possible interventions and actions to achieve the client's goals and provide the informal caregiver with the right support should be discussed within the reablement team. These interventions and actions are derived from the available toolkit. Afterwards, the care coordinator should determine the final interventions and actions with the client and informal caregiver, guided by their preferences and

For peer review only - http://bmjopen.bmj.com/site/about/guidelines.xhtml

capabilities, possibilities of the social network and the physical environment. Subsequently, the care plan should be recorded in the client's ECF and shared with all members of the reablement team. The intensity of the programme depends on the care needs of the client and their pre-set goals, which may require a higher intensity in the beginning but less at the end when (sub-)goals are (partly) reached.

Phase 4: Care delivery

The reablement team should deliver care and support as described in the care plan. Care delivery should be coordinated by the OT, who is also responsible for assisting with (re)learning and practicing meaningful daily activities. The RN is responsible for supporting managing the client's personal care needs. The PT, when part of the reablement team, is responsible for functional training and stimulating participation in daily life. The client's environment should be adapted (e.g. home modifications, assistive devices, and care technology), to support them in (re)learning activities. While supporting meaningful activities, the client's self-management should be stimulated by practicing activities and ensuring that tasks are not taken over by care professionals or informal caregivers. If necessary, a social worker should provide the informal caregiver with additional support to decrease their burden (e.g. respite care).

Phase 5: Evaluation

The evaluation of the care process should be structurally embedded in interdisciplinary team meetings. Based on conclusions drawn during these meetings, the care coordinator can decide whether the care plan is continued, adjusted or terminated. This must be discussed with the client and informal caregiver. At the end of the programme, a formal evaluation of the client's goals should take place using COPM [46], including scoring the performance and satisfaction within activities. Afterwards, the care coordinator decides with the client and informal caregiver if the programme

For peer review only

For peer review only - http://bmjopen.bmj.com/site/about/guidelines.xhtml

should be extended (max. two weeks) to ensure all goals are sufficiently reached or if the client needs referral to usual care.

[Insert Figure 3 here]

For peer review only

For peer review only - http://bmjopen.bmj.com/site/about/guidelines.xhtml

DISCUSSION

This manuscript describes the development and content of I-MANAGE, a community-based reablement programme. The programme was developed for community-dwelling older adults to improve their self-management and participation in daily life and ensure that they can remain living at home independently as long as possible and avoid unnecessary transitions to institutional care, while also increasing QoL and decreasing informal caregiver burden.

The programme contains several key elements that are considered essential. Firstly, interdisciplinary collaboration is important in I-MANAGE (e.g. appointing a care coordinator, scheduling (bi-)weekly meetings, and a shared ECF). Recent literature indicates that investing in interdisciplinary collaboration stimulates patient-centred care by ensuring a holistic view of the client's situation and creates shared responsibility [48-50]. This is achieved by good communication and coordination within the team but also with the client and informal caregiver [51]. Moreover, delivery of the programme by an interdisciplinary team, including allied health, such as OTs, is deemed valuable because of their educational background [30, 52]. It also stimulates continuous learning and is experienced as exciting and constructive by care professionals [50, 51]. Additionally, the integrated practice-oriented and on the job training, where care professionals can learn from other disciplines, help to invest in the self-efficacy of care professionals. This is essential, because successfully changing behaviours remains a challenge. Secondly, at the start of the programme (phase 1 and 2), we implemented a standardised goal-setting tool, COPM [46], preceded by an extensive intake based on the principles of positive health [47]. Previous research has indicated that using standardised assessment or goal-setting tools could increase the effectiveness of reablement interventions [30]. Additionally, it increased client involvement and helps professionals to identify meaningful activities with the client [53]. Thirdly, when delivering care (phase 4), the programme integrates several important aspects. Firstly, supporting informal caregivers is assumed to contribute to the effectiveness of I-MANAGE. Previous research found that providing informal caregivers with the right psychosocial and educational support strengthens their ability to cope with their new role [54, 55].

For peer review only - http://bmjopen.bmj.com/site/about/guidelines.xhtml

This is an important addition to most reablement programmes because, although making use of the clients' social network is a strategy to reach their goals according to the internationally accepted definition [18], this is often overlooked [56, 57]. Additionally, I-MANAGE promotes optimal use of the social and physical environment, which is essential since a demanding environment can either stimulate or hinder a person's participation in meaningful activities [3, 58]. Lastly, the programme stimulates self-management through participation in meaningful daily activities, which is a core element within reablement ('doing with…' rather than 'doing for…' the client) [18, 59].

The described programme serves as a model and leaves room to tailor the intervention to a specific context and the needs of the organisation. For example, the programme leaves room for the organisation to choose which target group would benefit most. Additionally, delivering the programme by an interdisciplinary team is a prerequisite. However, depending on available resources, the organisation can decide on the composition of the reablement team. Moreover, I-MANAGE is a multi-component programme in which organisations can integrate their own innovative practices, especially when working towards and reaching clients' meaningful goals. Moe and Brinchmann [25] confirmed the necessity of tailoring reablement services to local conditions by arguing that establishing reablement in an existing organisational structure is a complex process. Apart from available resources, during the co-creation process, some influencing factors for implementation were identified. For example, different IT systems, which make communication and information transfer less evident. Moe and Brinchmann [25] mentioned that, next to communication and IT systems, habitual ways of offering health services, a lack of knowledge about the rehabilitation potential of older adults and active ageing benefits are also experienced as barriers. A recent scoping review identified several factors that act as barriers and facilitators during the implementation of care innovations, among which, available resources and communication were listed as possible barriers to implementation [60]. It is critical to consider these factors when implementing I-MANAGE in practice. In order to overcome several barriers to the implementation of I-MANAGE, it could be useful to set up advisory boards and working groups to discuss the implementation process and adjust where needed.

For peer review only - http://bmjopen.bmj.com/site/about/guidelines.xhtml

Additionally, investing in getting the management of care organisations on board to support the implementation within their organisation and setting up knowledge exchange between sites where reablement is being implemented to share experiences and best practices could be beneficial. Investing in a suitable organisational structure is essential because it remains a challenge to successfully change existing organisational structures [25, 61, 62]. Regarding future research, further knowledge is needed to explore feasibility and (cost-)effectiveness of I-MANAGE, as it has not yet been proven. Since the programme is very context specific and can be tailored according to the needs and resources of an organisation, it would be beneficial to investigate what works for which target group and under what circumstances, for example, by means of a realist evaluation [63]. The programme is currently being implemented and evaluated at different Dutch care providers. The systematic reporting of the development of the programme provides useful insight for future research looking to develop complex health interventions or to implement a similar programme.

A strength of I-MANAGE and its development is that it is both theory and evidence-based [42, 43], which has proven to be advantageous when developing effective interventions [64, 65]. Moreover, Thuesen et al. [66] highlight the demand for making theory explicit in reablement interventions and in addressing the physical, psychological, and sociocultural perspectives of ageing within these interventions. Another strength of the development process was the co-creation process, which gave a voice to multiple stakeholders and made the programme suitable for practice. We used the development approach described by Bleijenberg et al. [41], which combined a range of published approaches to intervention development to enrich the MRC framework. This approach was chosen because using the MRC framework further assists us in evaluating and adapting the programme. However, we are aware that multiple approaches to intervention development exist as described by O'Cathain et al. [67]. These different approaches share many similarities (e.g. stepwise approach or involvement of stakeholders), but there are also significant differences (e.g. the focus on implementation or theory). It is important to acknowledge these differences and always chose an approach best suited to the purpose of the research. Additionally, most of these approaches are not

For peer review only - http://bmjopen.bmj.com/site/about/guidelines.xhtml

set in stone and leave room for the researchers own interpretation. It must also fit the setting and timing of the development process. There are also some limitations related to this co-creation process. Firstly, during the process, we always informed management to obtain their support, but they were not included in the working groups, which could have blocked important insight. Secondly, we aimed to include all end users of the programme during the development process (care professionals, clients, and informal caregivers). However, we only included client and informal caregiver representatives in the working groups and interviewed only two informal caregivers between sessions. Additionally, care professionals were recruited from the professional network of researchers and were recruited in a convenient way. We therefore did not ensure variation among these participants in terms of, for example, gender, age, or years of experience. Lastly, we obtained data from many different sources (i.e. scientific literature, individual interviews, working groups, and observations), which made it difficult to find common ground throughout all the sources and forced compromise. However, this use of data triangulation is also a strength of the development of the programme as findings could be checked multiple times with different sources, increasing the validity of the results.

For peer review only - http://bmjopen.bmj.com/site/about/guidelines.xhtml

FIGURE LEGENDS

Figure 1: The development process over time. This figure presents the development process over time from September 2019 until June 2021. The whole process is guided by scientific literature. The figure presents the two parts of the development process each with their related activities. The dark grey rectangles present the (intermediate) result of each part of the process. The number in between brackets represent the seven steps of the development phase as described by Bleijenberg et al. [41]. PT = physiotherapist; GR = geriatric rehabilitation; DSW = domestic support worker; RN = registered nurse.

Figure 2: Logic model of the I-MANAGE programme. The logic model of the I-MANAGE programme presents the aim and associated sub-aims of the programme, the programme components, and proximal and distal outcomes.

Figure 3: Detailed presentation of the I-MANAGE care process. I-MANAGE has a 5-phase care process (initiation, intake, care plan, care delivery, and evaluation), preceded with the referral of the client through different routes. After an average duration of 8 weeks, clients are referred to aftercare. Interdisciplinary collaboration is a continuous element of the programme.

ETHICS APPROVAL AND CONSENT TO PARTICIPATE

As participants were not subjected to actions and no rules of behaviour were imposed, no ethical approval was needed according to the Dutch ‘Medical Research Involving Human Subjects Act’ (WMO). Nevertheless, the study was carried out in accordance with the principles of ethical research and all participants involved in the working group sessions and interviews provided either written or verbal informed consent prior to the start of each session.

AVAILABILITY OF DATA AND MATERIALS

For peer review only - http://bmjopen.bmj.com/site/about/guidelines.xhtml

The datasets used and/or analysed during the current study available from the corresponding author on reasonable request.

COMPETING INTEREST

The authors declare that they have no competing interests

FUNDING

This work was supported by the European Union's Horizon 2020 research and innovation programme under the Marie Skłodowska-Curie grant agreement No 812656 as part of the TRANS-SENIOR project (www.trans-senior.eu).

AUTHORS' CONTRIBUTORS

All authors – IM, HV, GIJMK, JCMvH, EV, GG, SFM – were involved in the development of the I-MANAGE programme. IM wrote the first draft of the article. All authors reviewed the manuscript and approved the final version.

ACKNOWLEDGEMENTS

We would like to thank the Dutch stakeholders who have participated in the working group sessions, observations, and individual interviews, and therefore have helped with the development of a reablement programme suitable for the Dutch home care setting: Angelica de Lang, Nelly Jacobs, Jose Blezer, Belkis Bozdemir, Marcel Mannens, Marc Lowis, Maud Scholtes, Mandy Boosten, Hella Senden, Angèle van Kasteren, Anne Gulpers, Eveline van Velthuijsen, Anouschka Feenstra, Elfi Pakbeers, Nadine Klinkers, Marjo Gartener (MeanderGroep Zuid-Limburg), Ewout Smit (Vivium Zorggroep), Teuni Rooijackers, Angelique de Rijk (Maastricht University), Bianca Miltenburg (Cicero Zorggroep), Marie-Therese Bindels, Femke Scheuerman (gemeente Kerkrade), Marja Veenstra (Burgerkracht Limburg).

For peer review only - http://bmjopen.bmj.com/site/about/guidelines.xhtml

For peer review only

SUPPLEMENTAL MATERIAL

Supplementary file 1: GUIDED checklist. Completed guideline for reporting for intervention development studies.

Supplementary file 2: Overview of the different stakeholders involved per research activity.

Supplementary file 3: Overview of the intermediate results of the first five steps of the development phase.

For peer review only

REFERENCES

1. Zingmark M, Norström F. Transitions between levels of dependency among older people receiving social care – a retrospective longitudinal cohort study in a Swedish municipality. BMC Geriatr. 2021 2021/06/02;21(1):342.
2. Brown GC. Living too long: the current focus of medical research on increasing the quantity, rather than the quality, of life is damaging our health and harming the economy. EMBO Rep. 2015;16(2):137-41.
3. Rustad EC, Furnes B, Cronfalk BS, et al. Older patients' experiences during care transition. Patient Prefer Adherence. 2016;10:769-79.
4. Wahl HW, Fange A, Oswald F, et al. The home environment and disability-related outcomes in aging individuals: What is the empirical evidence? Gerontologist. 2009 Jun;49(3):355-67.
5. Arnau A, Espaulella J, Serrarols M, et al. Risk factors for functional decline in a population aged 75 years and older without total dependence: A one-year follow-up. Arch Gerontol Geriatr. 2016;65:239-47.
6. Tappenden P, Campbell F, Rawdin A, et al. The clinical effectiveness and cost-effectiveness of home-based, nurse-led health promotion for older people: A systematic review. Health Technol Assess. 2012;16(20):1-72.
7. Fujisawa R, Colombo F. The Long-Term Care Workforce: Overview and Strategies to Adapt Supply to a Growing Demand. 2009.
8. Arbaje AI, Kansagara DL, Salanitro AH, et al. Regardless of age: Incorporating principles from geriatric medicine to improve care transitions for patients with complex needs. J Gen Intern Med. 2014;29(6):932-9.
9. Koopmans RT, Lavrijsen JC, Hoek JF, et al. Dutch elderly care physician: a new generation of nursing home physician specialists. J Am Geriatr Soc. 2010;58(9):1807-9.
10. Nicholson C, Meyer J, Flatley M, et al. The experience of living at home with frailty in old age: A psychosocial qualitative study. Int J Nurs Stud. 2013 2013/09/01/;50(9):1172-9.

For peer review only - http://bmjopen.bmj.com/site/about/guidelines.xhtml

11. Aspinal F, Glasby J, Rostgaard T, et al. New horizons: Reablement - supporting older people towards independence. Age Ageing. 2016;45(5):572-6.

12. Beard JR, Officer A, de Carvalho IA, et al. The World report on ageing and health: a policy framework for healthy ageing. The Lancet. 2016;387(10033):2145-54.

13. Ferrah N, Ibrahim JE, Kipsaina C, et al. Death Following Recent Admission Into Nursing Home From Community Living: A Systematic Review Into the Transition Process. J Aging Health. 2018;30(4):584-604.

14. Oliver D, Foot C, Humphries R. Making our health and care systems fit for an ageing population: King's Fund London: UK; 2014.

15. Wiles JL, Leibing A, Guberman N, et al. The meaning of "aging in place" to older people. Gerontologist. 2012 Jun;52(3):357-66.

16. Cesari M, Vellas B, Hsu F-C, et al. A physical activity intervention to treat the frailty syndrome in older persons-results from the LIFE-P study. J Gerontol A Biol Sci Med Sci. 2015;70(2):216-22.

17. Daniels R, Metzelthin SF, van Rossum E, et al. Interventions to prevent disability in frail community-dwelling older persons: An overview. Eur J Ageing. 2010;7(1):37-55.

18. Metzelthin SF, Rostgaard T, Parsons M, et al. Development of an internationally accepted definition of reablement: a Delphi study. Ageing and Society. 2020:1-16.

19. Doh D, Smith R, Gevers P. Reviewing the reablement approach to caring for older people. Ageing and Society. 2019:1-13.

20. Metzelthin SF, Zijlstra GA, van Rossum E, et al. 'Doing with ...' rather than 'doing for ...' older adults: rationale and content of the 'Stay Active at Home' programme. Clin Rehabil. 2017;31(11):1419-30.

21. Tessier A, Beaulieu M-D, McGinn CA, et al. Effectiveness of Reablement: A Systematic Review. Healthcare policy. 2016;11(4):49-59.

For peer review only

22. Whitehead PJ, Worthington EJ, Parry RH, et al. Interventions to reduce dependency in personal activities of daily living in community dwelling adults who use homecare services: A systematic review. Clin Rehabil. 2015 Nov;29(11):1064-76.

23. Resnick B, Galik E, Boltz M. Function focused care approaches: Literature review of progress and future possibilities. J Am Med Dir Assoc. 2013 2013/05/01/;14(5):313-8.

24. Parsons J, Burton E, Graff L, et al. 4: Reablement as an evolution in home care: a comparison of implementation across five countries. In: Rostgaard T, Parsons J, Tuntland H, editors. Reablement in Long-Term Care for Older People: International Perspectives and Future Directions. Bristol: Policy Press; 2023. p. 68-92.

25. Moe C, Brinchmann BS. Tailoring reablement: A grounded theory study of establishing reablement in a community setting in Norway. Health Soc Care Community. 2018 Jan;26(1):113-21.

26. Wess T, Steiner W, Dür M, et al. Reablement – relevant factors for implementation: an exploratory sequential mixed-methods study design. BMC Health Serv Res. 2022 2022/07/28;22(1):959.

27. Rostgaard T, Tuntland H, Parsons J. 1: Introduction: The concept, rationale, and implications of reablement. In: Rostgaard T, Parsons J, Tuntland H, editors. Reablement in Long-Term Care for Older People: International Perspectives and Future Directions. Bristol: Policy Press; 2023. p. 3-20.

28. Mjøsund HL, Moe CF, Burton E, et al. Integration of Physical Activity in Reablement for Community Dwelling Older Adults: A Systematic Scoping Review. J Multidiscip Healthc. 2020;13:1291-315.

29. Duncan E, Cathain A, Rousseau N, et al. Guidance for reporting intervention development studies in health research (GUIDED): an evidence-based consensus study. BMJ Open. 2020;10(4):e033516.

For peer review only - http://bmjopen.bmj.com/site/about/guidelines.xhtml

30. Buma LE, Vluggen S, Zwakhalen S, et al. Effects on clients' daily functioning and common features of reablement interventions: a systematic literature review. Eur J Ageing. 2022 2022/05/03.

31. Assander S, Bergström A, Eriksson C, et al. ASSIST: a reablement program for older adults in Sweden – a feasibility study BMC Geriatr. 2022 2022/07/26;22(1):618.

32. Bergström A, Borell L, Meijer S, et al. Evaluation of an intervention addressing a reablement programme for older, community-dwelling persons in Sweden (ASSIST 1.0): a protocol for a feasibility study. BMJ Open. 2019;9(7):e025870.

33. Zingmark M, Kylén M. Feasibility of a reablement-program in a Swedish municipality. Scand J Occup Ther. 2023 2023/01/02;30(1):53-64.

34. Hoffmann TC, Glasziou PP, Boutron I, et al. Better reporting of interventions: template for intervention description and replication (TIDieR) checklist and guide. BMJ : British Medical Journal. 2014;348:g1687.

35. Craig P, Dieppe P, Macintyre S, et al. Developing and evaluating complex interventions: the new Medical Research Council guidance. BMJ. 2008;337:a1655.

36. Skivington K, Matthews L, Simpson SA, et al. A new framework for developing and evaluating complex interventions: update of Medical Research Council guidance. BMJ. 2021;374:n2061.

37. Bigonnesse C, Chaudhury H. The Landscape of "Aging in Place" in Gerontology Literature: Emergence, Theoretical Perspectives, and Influencing Factors. Journal of Aging and Environment. 2020 2020/07/02;34(3):233-51.

38. Kroneman M, Boerma W, van den Berg M, et al. Netherlands: Health System Review. Health Syst Transit. 2016 Mar;18(2):1-240.

39. Genet N, Boerma W, Kroneman M, et al. Home Care Across Europe. Current Structure and Future Challenges2013.

40. Centers for Disease Control and Prevention. Evaluation guide. Developing and using a logic model. CDC Division for Heart Disease and Stroke Prevention.

For peer review only - http://bmjopen.bmj.com/site/about/guidelines.xhtml

41. Bleijenberg N, de Man-van Ginkel JM, Trappenburg JCA, et al. Increasing value and reducing waste by optimizing the development of complex interventions: Enriching the development phase of the Medical Research Council (MRC) Framework. Int J Nurs Stud. 2018;79:86-93.

42. Mouchaers I, Van Haastregt JCM, Verbeek H, et al. « Reablement » comme outil thérapeutique pour offrir aux personnes âgées une vie de qualité à domicile après une réadaptation gériatrique. Rev Med Brux. 2021;42(4):324-32.

43. Mouchaers I, Verbeek H, Kempen GIJM, et al. The concept of disability and its causal mechanisms in older people over time from a theoretical perspective: a literature review. Eur J Ageing. 2022 2022/01/29.

44. Braun V, Clarke V. Using thematic analysis in psychology. Qualitative Research in Psychology. 2006 2006/01/01;3(2):77-101.

45. Campbell AJ, Robertson MC. Het Otago Oefenprogramma om valincidenten bij ouderen te voorkomen. Amersfoort: Nederlandse Vereniging voor Fysiotherapie in de Geriatrie; 2013.

46. Law M, Baptiste S, McColl M, et al. The Canadian Occupational Performance Measure: An Outcome Measure for Occupational Therapy. Canadian Journal of Occupational Therapy. 1990;57(2):82-7.

47. Huber M, van Vliet M, Giezenberg M, et al. Towards a 'patient-centred' operationalisation of the new dynamic concept of health: a mixed methods study. BMJ Open. 2016;6(1):e010091.

48. Schmutz JB, Meier LL, Manser T. How effective is teamwork really? The relationship between teamwork and performance in healthcare teams: a systematic review and meta-analysis. BMJ Open. 2019;9(9):e028280.

49. Ebrahimi Z, Patel H, Wijk H, et al. A systematic review on implementation of person-centered care interventions for older people in out-of-hospital settings. Geriatric Nursing. 2021 2021/01/01/;42(1):213-24.

50. Eliassen M, Moholt JM. Boundary work in task-shifting practices - a qualitative study of reablement teams. Physiother Theory Pract. 2022 Apr 18:1-14.

For peer review only - http://bmjopen.bmj.com/site/about/guidelines.xhtml

51. Birkeland A, Tuntland H, Førland O, et al. Interdisciplinary collaboration in reablement - a qualitative study. J Multidiscip Healthc. 2017;10:195-203.

52. Reeves S, Fletcher S, Barr H, et al. A BEME systematic review of the effects of interprofessional education: BEME Guide No. 39. Med Teach. 2016 Jul;38(7):656-68.

53. Rose A, Rosewilliam S, Soundy A. Shared decision making within goal setting in rehabilitation settings: A systematic review. Patient Educ Couns. 2017 Jan;100(1):65-75.

54. Lopez-Hartmann M, Wens J, Verhoeven V, et al. The effect of caregiver support interventions for informal caregivers of community-dwelling frail elderly: a systematic review. Int J Integr Care. 2012 Jul-Sep;12:e133.

55. Spiers GF, Liddle J, Kunonga TP, et al. What are the consequences of caring for older people and what interventions are effective for supporting unpaid carers? A rapid review of systematic reviews. BMJ Open. 2021;11(9):e046187.

56. Hjelle KM, Alvsvåg H, Førland O. The relatives' voice: how do relatives experience participation in reablement? A qualitative study. Journal of multidisciplinary healthcare. 2016;10:1-11.

57. van Wieringen M, Broese van Groenou MI, Groenewegen P. Impact of home care management on the involvement of informal caregivers by formal caregivers. Home Health Care Serv Q. 2015 Apr-Jun;34(2):67-84.

58. de Vries NM, Staal JB, van Ravensberg CD, et al. Outcome instruments to measure frailty: A systematic review. Ageing Res Rev. 2011 2011/01/01/;10(1):104-14.

59. Clotworthy A, Kusumastuti S, Westendorp RGJ. Reablement through time and space: a scoping review of how the concept of 'reablement' for older people has been defined and operationalised. BMC Geriatr. 2021 2021/01/15;21(1):61.

60. Fakha A, Groenvynck L, de Boer B, et al. A myriad of factors influencing the implementation of transitional care innovations: a scoping review. Implementation Science. 2021 2021/02/26;16(1):21.

For peer review only - http://bmjopen.bmj.com/site/about/guidelines.xhtml

61. Reay T, Goodrick E, D'Aunno T. Health Care Research and Organization Theory. Cambridge: Cambridge University Press; 2021. Available from: https://www.cambridge.org/core/elements/health-care-research-and-organization-theory/18F375C5D5DEBE92B725FAB547EA076D.

62. Rooijackers T, van Rossum E, Zijlstra GAR, et al. Effectiveness of a reablement training program on self-efficacy and outcome expectations regarding client activation in homecare staff: A cluster randomized controlled trial. Geriatric Nursing. 2022 2022/01/01/;43:104-12.

63. Pawson R, Tilley N. Realistic evaluation. London: Sage; 1997.

64. Michie S, Prestwich A. Are interventions theory-based? Development of a theory coding scheme. Health Psychol. 2010 Jan;29(1):1-8.

65. Bartholomew LK, Mullen PD. Five roles for using theory and evidence in the design and testing of behavior change interventions. J Public Health Dent. 2011;71(s1):S20-S33.

66. Thuesen J, Feiring M, Doh D, et al. Reablement in need of theories of ageing: would theories of successful ageing do? Ageing and Society. 2021:1-13.

67. O'Cathain A, Croot L, Sworn K, et al. Taxonomy of approaches to developing interventions to improve health: a systematic methods overview. Pilot and Feasibility Studies. 2019 2019/03/12;5(1):41.

For peer review only

For peer review only - http://bmjopen.bmj.com/site/about/guidelines.xhtml

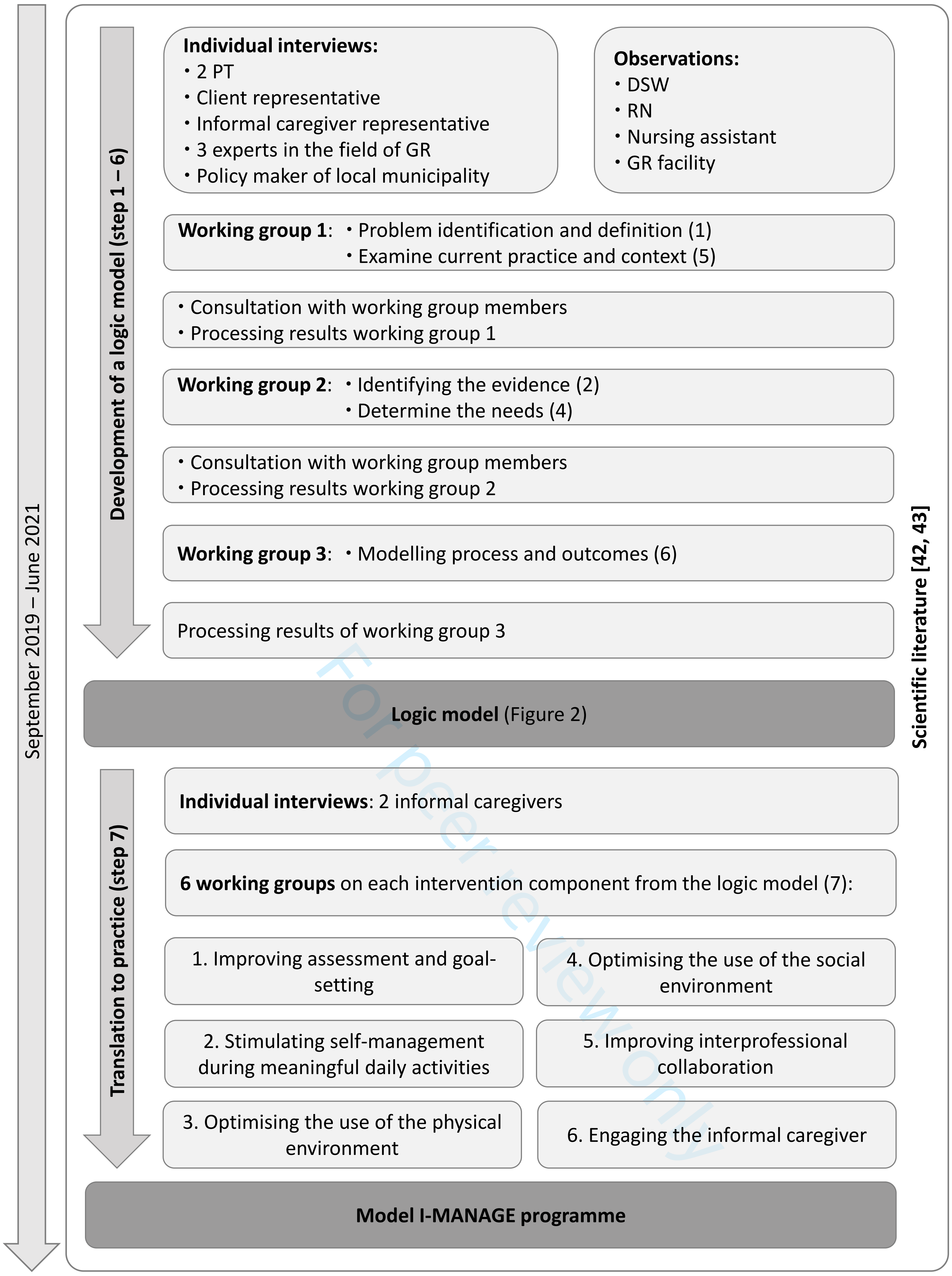

For peer review only

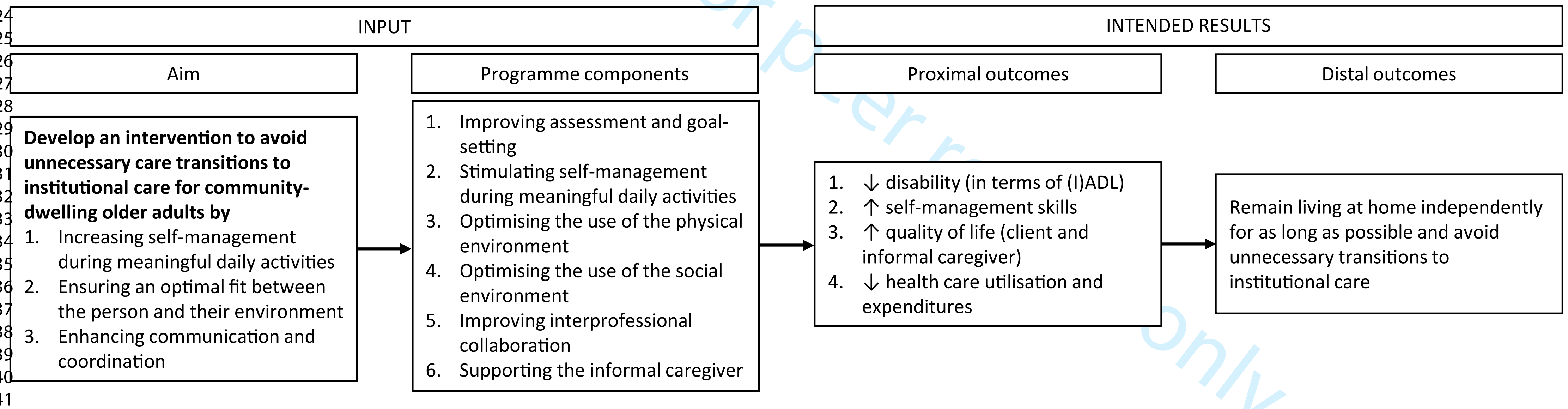

For peer review only

Referral of the client to I-MANAGE programme:
- Community nurses
- General practitioner
- Institutional care facilities

Start of the programme

Phase 1: Initiation
1. Provide information to client (and informal caregiver)
2. First visit by OT
    - Exploratory conversation based on Positive Health
3. If discharged from other care facility; initiate smooth handover

Week 1

Phase 2: Intake
1. Environmental assessment by OT
2. Set meaningful goals by using COPM
    - Guided by exploratory conversation from phase 1
3. Intake with informal caregiver to assess burden and needs

Week 2

Phase 3: Care plan
1. Determine interventions and actions to reach goals
    - Guided by preferences of client/informal caregiver
    - Specific attention for client's capabilities, social network and physical environment
2. Recorded in electronic care file

Week 3-8

Phase 4: Care delivery
1. Care provided as described in care plan
2. Follow-up on the care process by reablement team
3. If needed, initiate additional support for informal caregiver

Week 8

Phase 5: Evaluation
1. Continuous evaluation during care visit and team meetings
    - Continue, adjust or terminate care plan
2. Formal evaluation after 8 weeks by using COPM

Aftercare
1. Continue programme for maximum of 2 weeks
2. Referral to usual care

Interdisciplinary collaboration:
- Appointed care coordinator
- Regular team meetings
- Shared electronic care file
- Coaching on the job

For peer review only - http://bmjopen.bmj.com/site/about/guidelines.xhtml

Supplementary file 1. GUIDED checklist. A guideline for reporting for intervention development studies.

Item description	Explanation	Page in manuscript where item is located	Other*
Report the context for which the intervention was developed.	Understanding the context in which an intervention was developed informs readers about the suitability and transferability of the intervention to the context in which they are considering evaluating, adapting or using the intervention. Context here can include place, organisational and wider socio- political factors that may influence the development and/or delivery of the intervention.	P 8 – 9	
Report the purpose of the intervention development process.	Clearly describing the purpose of the intervention specifies what it sets out to achieve. The purpose may be informed by research priorities, for example those identified in systematic reviews, evidence gaps set out in practice guidance such as The National Institute for Health and Care Excellence or specific prioritisation exercises such as those undertaken with patients and practitioners through the James Lind Alliance.	P 6 – 7	
Report the target population for the intervention development process.	The target population is the population that will potentially benefit from the intervention – this may include patients, clinicians, and/or members of the public. If the target population is clearly described then readers will be able to understand the relevance of the intervention to their own research or practice. Health inequalities, gender and ethnicity are features of the target population that may be relevant to intervention development processes.	P 8 – 9	
Report how any published intervention development approach contributed to the development process.	Many formal intervention development approaches exist and are used to guide the intervention development process (e.g. Squid or The Person Based Approach to Intervention Development). Where a formal intervention development approach is used, it is helpful to describe the process that was followed, including any deviations. More general approaches to intervention development also exist and have been categorised as follows: Target Population-centred intervention development; evidence and theory-based intervention development; partnership intervention development; implementation-based intervention development; efficacy- based intervention development; step or phased-based intervention development; and intervention-specific intervention development. These approaches do not always have specific guidance that describe their use. Nevertheless, it is helpful to give a rich description of how any published approach was operationalised	P 8	

For peer review only

Report how evidence from different sources informed the intervention development process.	Intervention development is often based on published evidence and/or primary data that has been collected to inform the intervention development process. It is useful to describe and reference all forms of evidence and data that have informed the development of the intervention because evidence bases can change rapidly, and to explain the manner in which the evidence and/or data was used. Understanding what evidence was and was not available at the time of intervention development can help readers to assess transferability to their current situation.	P 10	Supplementary file 3 + [1]
Report how/if published theory informed the intervention development process.	Reporting whether and how theory informed the intervention development process aids the reader's understanding of the theoretical rationale that underpins the intervention. Though not mentioned in the e-Delphi or consensus meeting, it became increasingly apparent through the development of our guidance that this theory item could relate to either existing published theory or programme theory.	P 10	Supplementary file 3 + [2]
Report any use of components from existing interventions in the current intervention development process.	Some interventions are developed with components that have been adopted from existing interventions. Clearly identifying components that have been adopted or adapted and acknowledging their original source helps the reader to understand and distinguish between the novel and adopted components of the new intervention.	P 10	Supplementary file 3 + [1]
Report any guiding principles, people or factors that were prioritised when making decisions during the intervention development process.	Reporting any guiding principles that governed the development of the application helps the reader to understand the authors' reasoning behind the decisions that were made. These could include the examples of particular populations who views are being considered when designing the intervention, the modality that is viewed as being most appropriate, design features considered important for the target population, or the potential for the intervention to be scaled up.	P 9 – 11	Supplementary file 3
Report how stakeholders contributed to the intervention development process.	Potential stakeholders can include patient and community representatives, local and national policy makers, health care providers and those paying for or commissioning health care. Each of these groups may influence the intervention development process in different ways. Specifying how differing groups of stakeholders contributed to the intervention development process helps the reader to understand how stakeholders were involved and the degree of influence they had on the overall process. Further detail on how to integrate stakeholder contributions within intervention reporting are available.	P 9 – 11	
Report how the intervention	Intervention development is frequently an iterative process. The conclusion of the initial	P 13 – 18	

For peer review only

changed in content and format from the start of the intervention development process.	phase of intervention development does not necessarily mean that all uncertainties have been addressed. It is helpful to list remaining uncertainties such as the intervention intensity, mode of delivery, materials, procedures, or type of location that the intervention is most suitable for. This can guide other researchers to potential future areas of research and practitioners about uncertainties relevant to their healthcare context.		
Report any changes to the intervention required or likely to be required for subgroups.	Specifying any changes that the intervention development team perceive are required for the intervention to be delivered or tailored to specific sub groups enables readers to understand the applicability of the intervention to their target population or context. These changes could include changes to personnel delivering the intervention, to the content of the intervention, or to the mode of delivery of the intervention.	P 20 – 21	
Report important uncertainties at the end of the development process.	Intervention development is frequently an iterative process. The conclusion of the initial phase of intervention development does not necessarily mean that all uncertainties have been addressed. It is helpful to list remaining uncertainties such as the intervention intensity, mode of delivery, materials, procedures, or type of location that the intervention is most suitable for. This can guide other researchers to potential future areas of research and practitioners about uncertainties relevant to their healthcare context.	P 21 – 22	
Follow TIDieR guidance when describing the developed intervention.	Interventions have been poorly reported for a number of years. In response to this, internationally recognized guidance has been published to support the high quality reporting of health care interventions and public health interventions. This guidance should therefore be followed when describing a developed intervention.		
Brief name.	Provide the name or a phrase that describes the intervention.	P 1	Title page
Rationale, theory and aim of the elements essential to the intervention.	Describe any rationale, theory, or goal of the elements essential to the intervention.	P 13	Supplementary file 3 + [2]
Materials used in the intervention delivery or in the training of intervention providers.	Describe any physical or informational materials used in the intervention, including those provided to participants or used in intervention delivery or in training of intervention providers. Provide information on where the materials can be accessed (e.g. online appendix, URL).	P 13 – 18	
Procedures, activities and/or processes used including enabling	Describe each of the procedures, activities, and/or processes used in the intervention, including any enabling or support activities.	P 13 – 18	

For peer review only

or support activities.			
Expertise and background of intervention providers.	For each category of intervention provider (e.g. psychologist, nursing assistant), describe their expertise, background and any specific training given.	P 14 – 18	
How the intervention was provided.	Describe the modes of delivery (e.g. face-to-face or by some other mechanism, such as internet or telephone) of the intervention and whether it was provided individually or in a group.	P 13 – 18	
Location where the intervention occurred (incl. necessary infrastructure or relevant features).	Describe the type(s) of location(s) where the intervention occurred, including any necessary infrastructure or relevant features.	P 8 – 9	
When and how much the intervention was delivered.	Describe the number of times the intervention was delivered and over what period of time including the number of sessions, their schedule, and their duration, intensity or dose.	P 13 – 18	
Tailoring – what, why, when and how.	If the intervention was planned to be personalised, titrated or adapted, then describe what, why, when, and how.	P 20 – 21	
Modifications. †	If the intervention was modified during the course of the study, describe the changes (what, why, when, and how).	N/A	
Strategies to improve or maintain fidelity.	If intervention adherence or fidelity was assessed, describe how and by whom, and if any strategies were used to maintain or improve fidelity, describe them.	P 20 – 21	
Adherence and fidelity.†	If intervention adherence or fidelity was assessed, describe the extent to which the intervention was delivered as planned.	N/A	
Report the intervention development process in an open access format.	Unless reports of intervention development are available people considering using an intervention cannot understand the process that was undertaken and make a judgement about its appropriateness to their context. It also limits cumulative learning about intervention development methodology and observed consequences at later evaluation, translation and implementation stages. Reporting intervention development in an open access (Gold or Green) publishing format increases the accessibility and visibility of intervention development research and makes it more likely to be read and used. Potential platforms for open access publication of intervention development include open access journal publications, freely accessible funder reports or a study web-page that details the intervention development process.		Aim of this manuscript

Note. * e.g. if item is reported elsewhere, then the location of this information can be stated here. † If completing the TIDieR checklist for a protocol, these items are not relevant to the protocol and cannot be described until the study is complete.

For peer review only

References

1. Mouchaers I, Van Haastregt JCM, Verbeek H, et al. « Reablement » comme outil thérapeutique pour offrir aux personnes âgées une vie de qualité à domicile après une réadaptation gériatrique. Rev Med Brux. 2021;42(4):324-32.

2. Mouchaers I, Verbeek H, Kempen GIJM, et al. The concept of disability and its causal mechanisms in older people over time from a theoretical perspective: a literature review. Eur J Ageing. 2022 2022/01/29.

For peer review only

Supplementary file 2. Overview of the different stakeholders involved per research activity.

**Stakeholders**[a] (n = 26)	**Observations**[b]	**Individual interviews**	**Working groups**								
			**Development of a logic model**[c]			**Translation to practice**[d]					
			1	2	3	1	2	3	4	5	6
Domestic support worker	•		•	•	•		•			•	
Registered nurse (n = 3)	•		•	•	•	•	•		•	•	
Nursing assistant	•										
Physiotherapist (n = 4)		•	•	•	•	•				•	
Expert in the field of GR research (n = 3)		•	•								
Informal caregiver (n = 3)		•									•
Policy maker of the local municipality (n = 2)		•		•	•			•	•		
Client representative		•	•	•					•		
Informal caregiver representative		•	•	•	•						
Occupational therapist (n = 2)			•	•	•	•	•	•			
Psychologist			•	•	•						•
Geriatrician						•					
Reablement researcher							•				
Manager community care team										•	
Consultant informal care									•		•

GR = geriatric rehabilitation

Note: [a] if n > 1, not all stakeholders participated in every indicated research activity for that group; [b] observations were performed by the researchers; [c] indicates the three consecutive working group sessions throughout which the logic model was being developed following step 1 to 6 as described by Bleijenberg et al. [1]; [d] indicated the 6 working groups related to each programme component described in the logic model (1 = improving assessment and goal-setting, 2 = stimulating self-management during meaningful daily activities, 3 = optimising the use of the physical environment, 4 = optimising the use of the social environment, 5 = improving interprofessional collaboration, and 6 = engaging the informal caregiver).

References

1. Bleijenberg N, de Man-van Ginkel JM, Trappenburg JCA, et al. Increasing value and reducing waste by optimizing the development of complex interventions: Enriching the development phase of the Medical Research Council (MRC) Framework. Int J Nurs Stud. 2018;79:86-93.

For peer review only

Supplementary file 3. Overview of the intermediate results of the first five steps of the development phase.

Programme development step	Results
1. Problem identification	The identified problems were situated in three main areas:<br>1. A lack of attention to meaningful daily activities. According to the working group, older persons were mainly focused on functional goals related to (I)ADL tasks rather than meaningful daily activities that increase their well-being. Additionally, the focus of care is often on eliminating diseases and symptoms instead of maintaining quality of life [1, 2];<br>2. There appeared a gap between the person's abilities, needs, and wishes, and the environment they reside in. When older people experience a deterioration in health status, their environment is often not adapted, i.e. the necessary home modifications and assistive devices are not always in place [3, 4];<br>3. Communication and coordination within the care trajectory of an older person. There is insufficient communication amongst different care professionals but also with older persons and informal caregivers [4-7].
2. Identifying the evidence	The identification of the evidence-based practices was mainly based on literature research. A literature review was performed to gain insight into current evidence on effective reablement programmes aimed at improving the independent living of older persons [8]. We specifically sought to gain a general understanding of different reablement programmes, their characteristics (e.g. duration), content, outcome measures, and effectiveness. Thirteen programmes were identified, all intended for (I)ADL-impaired community-dwelling older adults. The most common features were: 1) delivery by a trained and coordinated multidisciplinary team and 2) implementation of an intake assessment and goal-setting tool. The applied interventions focused on supporting (I)ADL and physical functioning, and improving home safety. Nine programmes showed a statistically significant improvement on at least one of the outcome measures (e.g. (I)ADL functioning).
3. Identifying or developing theory	A literature review was performed on the concept of disability and its working mechanisms [9] to identify a solid theoretical foundation for our programme (*step 3*). We concluded that by optimising the use of personal, as well as environmental, resources and focusing on health and functioning, rather than disability, an older person's independence and wellbeing could be improved, especially while performing meaningful daily activities in accordance with the person's needs and preferences.
4. Determine the needs	The determined needs are closely related to the identified problems. Stakeholders expressed the need for:<br>1. Standardised goal-setting at the beginning of the care process. Goals should be meaningful, embedded in the older person's own environment, and not solely focused on (I)ADL;<br>2. Timely assessment and, if necessary, adaptations to the home environment of the older person. This concerns adaptations to the built environment, as well as the availability of assistive devices. Additionally, the social environment of the older person needs to be assessed in order to make optimal use of it or provide additional support if the social network is rather weak;<br>3. Improved communication and coordination within the care trajectory of the older person. For example, a smooth handover of information between care professionals and organisations, involvement of the informal caregiver when discussing a client's care needs, multidisciplinary collaboration with clear roles for each provider, and the appointment of a care coordinator.
5. Examining current practice and context	When examining the current practice and context we focused on positive aspects and possible barriers for implementation. Possible barriers could be different financial arrangements and different communication systems within a multidisciplinary team. Positive aspects include the comprehensive assessment by community nurses at the start

For peer review only

	of receiving community care, a signalling role for domestic support workers, and the presence of various welfare initiatives in the Netherlands. A suggestion was made to include the principles of Positive Health [10] in the programme, a best practice that is already known and applied by some care professionals.

References

1. Nicholson C, Meyer J, Flatley M, et al. The experience of living at home with frailty in old age: A psychosocial qualitative study. Int J Nurs Stud. 2013 2013/09/01/;50(9):1172-9.
2. Aspinal F, Glasby J, Rostgaard T, et al. New horizons: Reablement - supporting older people towards independence. Age Ageing. 2016;45(5):572-6.
3. Q-Consult zorg. Inventarisatie zorginhoud GRZ: Het net opgehaald na zes jaar GRZ in de Zorgverzekeringswet. Q-Consult zorg, 2019.
4. Rustad EC, Furnes B, Cronfalk BS, et al. Older patients' experiences during care transition. Patient Prefer Adherence. 2016;10:769-79.
5. Everink IHJ, van Haastregt JCM, Kempen GIJM, et al. Uitdagingen in de geriatrische revalidatiezorg: de ontwikkeling van een zorgpad. Tijdschr Gerontol Geriatr. 2015 2015/04/01;46(2):104-12.
6. Bauer M, Fitzgerald L, Haesler E, et al. Hospital discharge planning for frail older people and their family. Are we delivering best practice? A review of the evidence. J Clin Nurs. 2009;18(18):2539-46.
7. Adelman RD, Tmanova LL, Delgado D, et al. Caregiver burden: a clinical review. JAMA. 2014;311(10):1052-60.
8. Mouchaers I, Van Haastregt JCM, Verbeek H, et al. « Reablement » comme outil thérapeutique pour offrir aux personnes âgées une vie de qualité à domicile après une réadaptation gériatrique. Rev Med Brux. 2021;42(4):324-32.
9. Mouchaers I, Verbeek H, Kempen GIJM, et al. The concept of disability and its causal mechanisms in older people over time from a theoretical perspective: a literature review. Eur J Ageing. 2022 2022/01/29.
10. Huber M, van Vliet M, Giezenberg M, et al. Towards a ‘patient-centred’ operationalisation of the new dynamic concept of health: a mixed methods study. BMJ Open. 2016;6(1):e010091.

For peer review only

Supplementary file 1. GUIDED checklist. A guideline for reporting for intervention development studies.

Item description	Explanation	Page in manuscript where item is located	Other*
Report the context for which the intervention was developed.	Understanding the context in which an intervention was developed informs readers about the suitability and transferability of the intervention to the context in which they are considering evaluating, adapting or using the intervention. Context here can include place, organisational and wider socio- political factors that may influence the development and/or delivery of the intervention.	P 8 – 9	
Report the purpose of the intervention development process.	Clearly describing the purpose of the intervention specifies what it sets out to achieve. The purpose may be informed by research priorities, for example those identified in systematic reviews, evidence gaps set out in practice guidance such as The National Institute for Health and Care Excellence or specific prioritisation exercises such as those undertaken with patients and practitioners through the James Lind Alliance.	P 6 – 7	
Report the target population for the intervention development process.	The target population is the population that will potentially benefit from the intervention – this may include patients, clinicians, and/or members of the public. If the target population is clearly described then readers will be able to understand the relevance of the intervention to their own research or practice. Health inequalities, gender and ethnicity are features of the target population that may be relevant to intervention development processes.	P 8 – 9	
Report how any published intervention development approach contributed to the development process.	Many formal intervention development approaches exist and are used to guide the intervention development process (e.g. Squid or The Person Based Approach to Intervention Development). Where a formal intervention development approach is used, it is helpful to describe the process that was followed, including any deviations. More general approaches to intervention development also exist and have been categorised as follows: Target Population-centred intervention development; evidence and theory-based intervention development; partnership intervention development; implementation-based intervention development; efficacy- based intervention development; step or phased-based intervention development; and intervention-specific intervention development. These approaches do not always have specific guidance that describe their use. Nevertheless, it is helpful to give a rich description of how any published approach was operationalised	P 8	

For peer review only

Report how evidence from different sources informed the intervention development process.	Intervention development is often based on published evidence and/or primary data that has been collected to inform the intervention development process. It is useful to describe and reference all forms of evidence and data that have informed the development of the intervention because evidence bases can change rapidly, and to explain the manner in which the evidence and/or data was used. Understanding what evidence was and was not available at the time of intervention development can help readers to assess transferability to their current situation.	P 10	Supplementary file 3 + [1]
Report how/if published theory informed the intervention development process.	Reporting whether and how theory informed the intervention development process aids the reader's understanding of the theoretical rationale that underpins the intervention. Though not mentioned in the e-Delphi or consensus meeting, it became increasingly apparent through the development of our guidance that this theory item could relate to either existing published theory or programme theory.	P 10	Supplementary file 3 + [2]
Report any use of components from existing interventions in the current intervention development process.	Some interventions are developed with components that have been adopted from existing interventions. Clearly identifying components that have been adopted or adapted and acknowledging their original source helps the reader to understand and distinguish between the novel and adopted components of the new intervention.	P 10	Supplementary file 3 + [1]
Report any guiding principles, people or factors that were prioritised when making decisions during the intervention development process.	Reporting any guiding principles that governed the development of the application helps the reader to understand the authors' reasoning behind the decisions that were made. These could include the examples of particular populations who views are being considered when designing the intervention, the modality that is viewed as being most appropriate, design features considered important for the target population, or the potential for the intervention to be scaled up.	P 9 – 11	Supplementary file 3
Report how stakeholders contributed to the intervention development process.	Potential stakeholders can include patient and community representatives, local and national policy makers, health care providers and those paying for or commissioning health care. Each of these groups may influence the intervention development process in different ways. Specifying how differing groups of stakeholders contributed to the intervention development process helps the reader to understand how stakeholders were involved and the degree of influence they had on the overall process. Further detail on how to integrate stakeholder contributions within intervention reporting are available.	P 9 – 11	
Report how the intervention	Intervention development is frequently an iterative process. The conclusion of the initial	P 13 – 18	

For peer review only

changed in content and format from the start of the intervention development process.	phase of intervention development does not necessarily mean that all uncertainties have been addressed. It is helpful to list remaining uncertainties such as the intervention intensity, mode of delivery, materials, procedures, or type of location that the intervention is most suitable for. This can guide other researchers to potential future areas of research and practitioners about uncertainties relevant to their healthcare context.		
Report any changes to the intervention required or likely to be required for subgroups.	Specifying any changes that the intervention development team perceive are required for the intervention to be delivered or tailored to specific sub groups enables readers to understand the applicability of the intervention to their target population or context. These changes could include changes to personnel delivering the intervention, to the content of the intervention, or to the mode of delivery of the intervention.	P 20 – 21	
Report important uncertainties at the end of the development process.	Intervention development is frequently an iterative process. The conclusion of the initial phase of intervention development does not necessarily mean that all uncertainties have been addressed. It is helpful to list remaining uncertainties such as the intervention intensity, mode of delivery, materials, procedures, or type of location that the intervention is most suitable for. This can guide other researchers to potential future areas of research and practitioners about uncertainties relevant to their healthcare context.	P 21 – 22	
Follow TIDieR guidance when describing the developed intervention.	Interventions have been poorly reported for a number of years. In response to this, internationally recognized guidance has been published to support the high quality reporting of health care interventions and public health interventions. This guidance should therefore be followed when describing a developed intervention.		
Brief name.	Provide the name or a phrase that describes the intervention.	P 1	Title page
Rationale, theory and aim of the elements essential to the intervention.	Describe any rationale, theory, or goal of the elements essential to the intervention.	P 13	Supplementary file 3 + [2]
Materials used in the intervention delivery or in the training of intervention providers.	Describe any physical or informational materials used in the intervention, including those provided to participants or used in intervention delivery or in training of intervention providers. Provide information on where the materials can be accessed (e.g. online appendix, URL).	P 13 – 18	
Procedures, activities and/or processes used including enabling	Describe each of the procedures, activities, and/or processes used in the intervention, including any enabling or support activities.	P 13 – 18	

For peer review only

or support activities.			
Expertise and background of intervention providers.	For each category of intervention provider (e.g. psychologist, nursing assistant), describe their expertise, background and any specific training given.	P 14 – 18	
How the intervention was provided.	Describe the modes of delivery (e.g. face-to-face or by some other mechanism, such as internet or telephone) of the intervention and whether it was provided individually or in a group.	P 13 – 18	
Location where the intervention occurred (incl. necessary infrastructure or relevant features).	Describe the type(s) of location(s) where the intervention occurred, including any necessary infrastructure or relevant features.	P 8 – 9	
When and how much the intervention was delivered.	Describe the number of times the intervention was delivered and over what period of time including the number of sessions, their schedule, and their duration, intensity or dose.	P 13 – 18	
Tailoring – what, why, when and how.	If the intervention was planned to be personalised, titrated or adapted, then describe what, why, when, and how.	P 20 – 21	
Modifications. †	If the intervention was modified during the course of the study, describe the changes (what, why, when, and how).	N/A	
Strategies to improve or maintain fidelity.	If intervention adherence or fidelity was assessed, describe how and by whom, and if any strategies were used to maintain or improve fidelity, describe them.	P 20 – 21	
Adherence and fidelity.†	If intervention adherence or fidelity was assessed, describe the extent to which the intervention was delivered as planned.	N/A	
Report the intervention development process in an open access format.	Unless reports of intervention development are available people considering using an intervention cannot understand the process that was undertaken and make a judgement about its appropriateness to their context. It also limits cumulative learning about intervention development methodology and observed consequences at later evaluation, translation and implementation stages. Reporting intervention development in an open access (Gold or Green) publishing format increases the accessibility and visibility of intervention development research and makes it more likely to be read and used. Potential platforms for open access publication of intervention development include open access journal publications, freely accessible funder reports or a study web-page that details the intervention development process.		Aim of this manuscript

Note. * e.g. if item is reported elsewhere, then the location of this information can be stated here. † If completing the TIDieR checklist for a protocol, these items are not relevant to the protocol and cannot be described until the study is complete.

For peer review only

References

1. Mouchaers I, Van Haastregt JCM, Verbeek H, et al. « Reablement » comme outil thérapeutique pour offrir aux personnes âgées une vie de qualité à domicile après une réadaptation gériatrique. Rev Med Brux. 2021;42(4):324-32.

2. Mouchaers I, Verbeek H, Kempen GIJM, et al. The concept of disability and its causal mechanisms in older people over time from a theoretical perspective: a literature review. Eur J Ageing. 2022 2022/01/29.

For peer review only

BMJ Open

Development and content of a community-based reablement programme (I-MANAGE): A co-creation study

Journal:	*BMJ Open*
Manuscript ID	bmjopen-2022-070890.R3
Article Type:	Original research
Date Submitted by the Author:	20-Jun-2023
Complete List of Authors:	Mouchaers, Ines; Maastricht University, Department of Health Services Research; Living Lab in Ageing and Long Term Care<br>Verbeek, Hilde ; Maastricht University, Department of Health Services Research; Living Lab in Ageing and Long Term Care<br>Kempen, Gertrudis; Maastricht University, Department of Health Services Research; Living Lab in Ageing and Long Term Care<br>van Haastregt, Jolanda; Maastricht University, Department of Health Services Research; Living Lab in Ageing and Long Term Care<br>Vlaeyen, Ellen; KU Leuven, Public Health and Primary Care; UHasselt, Faculty of Medicine and Life Sciences<br>Goderis, Geert; KU Leuven, Department of Public Health and Primary Care<br>Metzelthin, Silke; Maastricht University, Department of Health Services Research; Living Lab in Ageing and Long Term Care
**Primary Subject Heading**:	Health services research
Secondary Subject Heading:	Patient-centred medicine, Rehabilitation medicine
Keywords:	Health Services for the Aged, Patient-Centered Care, Primary Health Care, Quality of Life, Rehabilitation medicine < INTERNAL MEDICINE

SCHOLARONE™
Manuscripts

I, the Submitting Author has the right to grant and does grant on behalf of all authors of the Work (as defined in the below author licence), an exclusive licence and/or a non-exclusive licence for contributions from authors who are: i) UK Crown employees; ii) where BMJ has agreed a CC-BY licence shall apply, and/or iii) in accordance with the terms applicable for US Federal Government officers or employees acting as part of their official duties; on a worldwide, perpetual, irrevocable, royalty-free basis to BMJ Publishing Group Ltd ("BMJ") its licensees and where the relevant Journal is co-owned by BMJ to the co-owners of the Journal, to publish the Work in this journal and any other BMJ products and to exploit all rights, as set out in our licence.

The Submitting Author accepts and understands that any supply made under these terms is made by BMJ to the Submitting Author unless you are acting as an employee on behalf of your employer or a postgraduate student of an affiliated institution which is paying any applicable article publishing charge ("APC") for Open Access articles. Where the Submitting Author wishes to make the Work available on an Open Access basis (and intends to pay the relevant APC), the terms of reuse of such Open Access shall be governed by a Creative Commons licence – details of these licences and which Creative Commons licence will apply to this Work are set out in our licence referred to above.

Other than as permitted in any relevant BMJ Author's Self Archiving Policies, I confirm this Work has not been accepted for publication elsewhere, is not being considered for publication elsewhere and does not duplicate material already published. I confirm all authors consent to publication of this Work and authorise the granting of this licence.

For peer review only

TITLE PAGE

Title

Development and content of a community-based reablement programme (I-MANAGE): A co-creation study

Authors

Ines Mouchaers, MSc, [1, 2, 3 *]

Hilde Verbeek, PhD, [1, 2]

Gertrudis I.J.M. Kempen, PhD, [1, 2]

Jolanda C.M. van Haastregt, PhD, [1, 2]

Ellen Vlaeyen, PhD, [3, 5, 6]

Geert Goderis, PhD, [4]

Silke F. Metzelthin, PhD, [1, 2]

Author Affiliations

[1] Department of Health Services Research, Faculty of Health Medicine and Life Sciences, CAPHRI Care and Public Health Research Institute, Maastricht University, Maastricht, the Netherlands

[2] Living Lab in Ageing and Long Term Care, Maastricht, the Netherlands

[3] Department of Public Health and Primary Care, Academic Centre for Nursing and Midwifery, KU Leuven, Leuven, Belgium

[4] Department of Public Health and Primary Care, Academic Centre for General Practice, KU Leuven, Leuven, Belgium

[5] Faculty of Medicine and Life Sciences, Hasselt University, Hasselt, Belgium

[6] University Centre for Nursing and Midwifery, Department of Public Health and Primary Care, Faculty of Medicine and Health Sciences, Ghent University, Belgium

For peer review only

Corresponding author

Ines Mouchaers: https://orcid.org/0000-0001-7189-4468

* Address correspondence to: Ines Mouchaers, MSc, Department of Health Services Research, P.O. Box 616, 6200 MD Maastricht, The Netherlands.

E-mail: i.mouchaers@maastrichtuniversity.nl; Telephone number: +31-(0)43 388 28 15.

Word count

5218

For peer review only

ABSTRACT

Objectives: As age increases, people generally start experiencing problems related to independent living, resulting in an increased need for long-term care services. Investing in sustainable solutions to promote independent living is therefore essential. Subsequently, reablement is a concept attracting growing interest. Reablement is a person-centred, holistic approach promoting older adults' active participation through daily, social, leisure, and physical activities. The aim of this paper is to describe the development and content of I-MANAGE, a model for a reablement programme for community-dwelling older adults.

Design: The development of the programme was performed according to the Medical Research Council framework as part of the TRANS-SENIOR international training and research network. A co-creation design was used, including literature research, observations, interviews, and working group sessions with stakeholders.

Setting and participants: The interviews and working group sessions took place in the Dutch long-term home care context. Stakeholders invited to the individual interviews and working group sessions included care professionals, policymakers, client representatives, informal caregiver representatives, informal caregivers, and scientific experts.

Results: The co-creation process resulted in a 5-phase interdisciplinary primary care programme, called I-MANAGE. The programme focuses on improving the self-management and wellbeing of older adults by working towards their meaningful goals. During the programme, the person's physical and social environment will be put to optimal use, and sufficient support will be provided to informal caregivers to reduce their burden. Lastly, the programme aims for continuity of care and better communication and coordination.

Conclusion: The I-MANAGE programme can be tailored to the local practices and resources and is therefore suitable for the use in different settings, nationally and internationally. If the programme is implemented as described, it is important to closely monitor the process and results.

For peer review only - http://bmjopen.bmj.com/site/about/guidelines.xhtml

STRENGTHS AND LIMITATIONS OF THIS STUDY

- Intervention development was conducted using a systematic approach based on the Medical Research Council framework.
- The intervention and its development is theory and evidence based as a result of extensive literature research.
- All end users were represented during the development process, increasing its acceptability and feasibility.
- Data triangulation was used during the development process, increasing validity of the results.
- Client and informal caregiver representatives were included in the working group instead of clients and informal caregivers themselves, possibly missing an important voice.

For peer review only

For peer review only - http://bmjopen.bmj.com/site/about/guidelines.xhtml

BACKGROUND

The amount of older adults experiencing disabilities will increase over time and, while a large proportion of the older population remains independent, others will experience an increased need for support [1]. Moreover, 50% of people aged 85 years or older require care and/or support with daily activities [2]. As a result, it is expected that their demands for long-term care services will increase. When older adults live in an environment that is unsafe and does not meet their needs, the challenges they might face regarding independent living will increase further [3]. Previous research showed that a maladjusted environment negatively affects disability, which could lead to an accumulation of health risks, loss of independence, poor quality of life (QoL), and depression [4-6]. Care and service delivery in the community is often fragmented, with little coordination and poor communication amongst care providers, clients, and informal caregivers [3, 7-9]. Furthermore, the focus of care is often on eliminating specific diseases and symptoms instead of supporting the remaining capacity to maintain QoL and independent living [10, 11]. Rather than performing tasks *with* their clients, care professionals often tend to take over [11]. Failing to properly tackle these challenges could increase the use of health and social care and related costs [12]. In addition, this could lead to unnecessary (re)hospitalisations or permanent nursing home placement, which each have their own risks (e.g. increased mortality) [13] and at a time when financial and workforce resources are shrinking [7, 12, 14]. It is therefore essential to invest in sustainable solutions to promote independent living [15-17].

A concept attracting growing interest in promoting independent living among older adults is reablement. This is a person-centred, holistic approach promoting active participation of older adults in daily activities through social, leisure, and physical activities chosen by the older adult in line with their preferences, either at home or in the community [18]. Instead of creating dependency by taking over tasks, care professionals identify the capabilities and opportunities of individuals to maximise their independence by supporting them to achieve their goals, through participation in daily activities, home modifications, assistive devices, and involvement of their social network [11, 18-20]. Current evidence on the effectiveness of reablement interventions is inconclusive [11], however several

For peer review only

systematic reviews have indicated the positive results of reablement relating to activities of daily living (ADL) functioning and health-related QoL [21-23]. Due to the promising results, interest in implementing reablement into everyday care is growing internationally. In Denmark, New Zealand and the United Kingdom, reablement has more-or-less been successfully implemented across the whole country [1]. For example, in Denmark reablement in long-term care for older adults was legally introduced in 2015, meaning that all municipalities must offer reablement interventions and all applicants for home care are assessed for potential for reablement before being offered conventional home care [2].

Despite the promising results and successful implementation abroad, contextual differences mean this is no indication that it would necessarily be effective in its current format in the Dutch home care setting [24]. Implementing reablement is a complex process and influenced by multiple factors, such as organisational factors, individual and social attitudes towards a new form of care, technological factors related to communication, and financial factors [25, 26]. It is crucial to critically consider these factors in the design, delivery, and evaluation of reablement [27]. Moreover, to implement reablement in the Dutch home care setting, existing programmes need to be revised and adapted to suit the current context, which is crucial when developing and delivering complex health care interventions [27]. However, the development and content of community care programmes, such as reablement programmes, are often insufficiently described in the scientific literature [19, 28-30]. There are only limited articles available that describe either the development of the programme or its content in detail [31-33] and these descriptions are often included as part of a feasibility or pilot study [29]. This offers little guidance to replicate or build on the previous findings of such programmes, despite this being essential for the development of new and implementation of existing programmes in different settings [29, 30, 34].

This paper therefore describes the development, using a co-creation process, and content of I-MANAGE, a reablement programme for community-dwelling older adults to improve older adults' self-management and participation in daily life, while also increasing QoL and decreasing informal

For peer review only - http://bmjopen.bmj.com/site/about/guidelines.xhtml

caregivers' burden. By describing the development and content of the programme in detail, we increase the replicability and prevent other researchers from reinventing the wheel. The programme is specifically suitable for the Dutch community care setting, however, due to its extensive description, this manuscript may also provide a model for implementation in other countries.

For peer review only

For peer review only - http://bmjopen.bmj.com/site/about/guidelines.xhtml

METHODS

To describe the development process of the programme and ensure completeness of reporting, we used the guidance for reporting for intervention development studies in health research (GUIDED) (Supplementary file 1) [29]. This checklist provides a clear and structured basis for the reporting of programme development, as well as the description of the content of the programme.

Design

I-MANAGE was developed between September 2019 and June 2021 using a co-creation design. The programme was developed following the first phase (development) of the Medical Research Council (MRC) framework for the development and evaluation of complex interventions [35, 36].

Patient and public involvement

Co-creation was initiated by the researchers as a response to the challenges that are caused by an ageing society (e.g., increasing care needs, decreasing staff). To deal with these challenges the Dutch government stimulates an ageing in place policy and promotes another way of (home) care delivery moving from “doing for…” towards “doing with…” clients, or in the best case to enable clients to do things by themselves again. These developments are also adopted by Dutch care organisations, which hope to improve the quality and sustainability of their care services [37]. All end users (care professionals who would be implementing and delivering the programme, as well as the target population) were represented and involved during the development process of the programme as members of the working groups and by participating in the individual interviews. However, they were not involved in the development of the study design of dissemination of the findings.

Setting and participants

I-MANAGE is based on international evidence and tailored to the Dutch home care context. Home care in the Netherlands includes personal care (i.e. assistance with ADL), nursing care (i.e. medical

For peer review only - http://bmjopen.bmj.com/site/about/guidelines.xhtml

For peer review only

assistance), and domestic support (i.e. assistance with instrumental activities of daily living (IADL)) [38]. Usually home care is funded by two statutory forms of insurance cover care: the *Health Insurance Act* (ZVW) and the *Social Support Act* (WMO). Clients often use a combination of ZVW (e.g. general practitioner care, therapists, hospital care, or medication), and WMO (e.g. domestic support, home adaptations) [38, 39]. The programme was developed for community-dwelling older adults, irrespective of age or cognitive and functional status.

The programme was developed in co-creation with stakeholders who participated in observations, individual interviews and working groups. Supplementary file 2 provides an overview of all stakeholders involved and the research activities they participated in. Participants were recruited from the professional network of the researchers. They were informed about the study and asked to participate via email. When participants agreed to participate, verbal or written informed consent was provided before the start of each interview or working group session.

Data collection

First, a logic model was created. A logic model is a tool to illustrate how a programme will create change [40]. The logic model systematically visualises the aim and sub-aims of the programme, the programme components, and the intended outcomes [40]. The logic model was developed using six iterative programme development steps: 1) identifying the problem, 2) identifying the evidence, 3) identifying or developing a theory, 4) determining needs, 5) examining current practices and the context, and 6) modelling processes and outcomes [41]. The logic model was then translated to practice (step 7). Data collection was performed following a non-linear and iterative process as described by Bleijenberg et al. [41], and by using a variety of data collection methods, including literature research, observations, individual interviews, and working groups. Figure 1 illustrates the development process in detail.

[Insert Figure 1 here]

For peer review only - http://bmjopen.bmj.com/site/about/guidelines.xhtml

For peer review only

Development of a logic model (steps 1 to 6)

All steps were guided by literature research, especially steps 2 and 3 of the development process. To gain insight into current evidence-based practices (*step 2*), the relevant scientific literature on reablement programmes was reviewed. The methodology and results of this literature research is described elsewhere [42]. To identify a theoretical foundation for the programme (*step 3*), a literature review was performed on the concept of disability and its underlying causal mechanisms [43].

Furthermore, a working group was composed and invited to three sessions (1.5 h each). Members of the working group included physiotherapists (PT), occupation therapists (OT), registered nurses (RN), a domestic support worker (DSW), a policymaker of the local municipality, a psychologist, a client representative, informal caregiver representatives, and a geriatric rehabilitation expert. The first session focused on the identification of the problem (*step 1*) and the examination of the current practice and context (*step 5*). Session two focused on the previously identified evidence, which participants could complement with practices from their own field of work (*step 2*), and the determination of needs (*step 4*), closely related to the previously identified problems. The third session focused on modelling the process and outcomes (*step 6*), during which a preliminary logic model was presented, on which participants could provide feedback. Between sessions, working group members were consulted for additional input and clarification if needed. The researchers processed the results from each session and the additional information in order to be used as a starting point for the next session. The working group sessions were led by the first and last author (IM and SM).

We then interviewed two PTs, a policy maker of the local municipality, a client and informal caregiver representative and three experts in the field of geriatric rehabilitation research. In total, eight interviews were conducted, all focusing on identifying the problem (*step 1*), determining the needs (*step 4*), and gaining insight into current practice and context (*step 5*). In addition, we interviewed two informal caregivers. Topics addressed during these interviews included their role as

For peer review only - http://bmjopen.bmj.com/site/about/guidelines.xhtml

informal caregiver, how involved they were in the care process, how they were or felt supported and by whom and what they would like to change in the process.

Lastly, observations were performed by the researchers. Observations were conducted in the traditional community care setting to examine current practice and context of home care services (*step 5*) and to identify problems (*step 1*). Six observations, each lasting half a day, were conducted by shadowing a DSW, an RN, and a nursing assistant, and three days were spent with allied health professionals. Field notes were taken throughout the observation periods.

Translation to practice (step 7)

For each of the six programme components in the logic model, we invited members of the working group, a geriatrician, informal care consultant, reablement researcher, informal caregiver, and a community care teams' manager to a session to translate components into practice (*step 7*). Based on the programme component, the most relevant stakeholders were invited. For example, on informal caregiver support, we invited an informal care consultant, an informal caregiver, and a psychologist; resulting in three to four members per working group. Each working group attended one session lasting 1 hour. Each session began with an introduction to the goal of the programme, the logic model, and the results of steps 1 to 6 related to the programme component. Afterwards, participants were asked open-ended questions about the practical implementation as well as barriers and facilitators of the component. Lastly, results were summarised and participants were asked for final feedback. Table 1 provides an overview of the different methodologies used in each development step.

Table 1. Overview of the different methodologies used in each development step defined by Bleijenberg et al. [41]

Programme development steps	Methodology			
	Literature research	Observations	Individual interviews	Working groups
1. Problem identification	○	●	●	●
2. Identifying the evidence	●			●
3. Identifying or developing theory	●			
4. Determine the needs	○		●	●
5. Examining current practice and context	○	●	●	●
6. Modelling process and outcomes	○	○	○	●

For peer review only - http://bmjopen.bmj.com/site/about/guidelines.xhtml

7.	Translation to practice	● (grey)	●

Note: The full black dots ● indicate a main source of information for the particular step, while the full grey dots ● indicate only a minor influence and guidance for the particular step.

Data analysis

We used data triangulation to verify the results. Our main source of information was the working group sessions, as they provided the richest data on the perspectives of the different target groups. Individual interviews, observations, and literature research were used to complement and check the information obtained throughout the working group sessions. Working group sessions and individual interviews were recorded and transcribed non-verbatim. A thematic analysis was conducted based on the steps described by Braun and Clarke [44]. Common themes were identified within each step of the development process (e.g. lack of sufficient communication). Afterwards, the themes from all sessions were compared and associations were found between, for example, identified problems and determined needs, or current scientific evidence and best practices of the organisation. The results of each session provided input and structure for the next session. After each working group session or individual interview, a member check was conducted by summarising the most important findings. The results were discussed regularly within the research team. The research team consists of all authors involved. They work in the field of social gerontology, public health and primary and long-term care. A research logbook was kept by the researchers to keep track of all research activities and intermediate results, together with field notes from these activities.

For peer review only - http://bmjopen.bmj.com/site/about/guidelines.xhtml

RESULTS

The following section presents the final model of the I-MANAGE programme in detail, starting with the developed logic model, and eventually the translation to practice, which contains a detailed description of all programme components. A detailed description of the results from the first five programme development steps is provided in Supplementary file 3.

Development of a logic model

The logic model starts with the aim and sub-aims of the programme based on identified problems and needs (*result from development steps 1 and 4*), which are derived mostly from the working group sessions and individual interviews. To fulfil these aims, six programme components were determined: 1) improving assessment and goal-setting; 2) stimulating self-management during meaningful daily activities; 3) optimising the use of the physical environment; 4) optimising the use of the social environment; 5) improving interprofessional collaboration; and 6) supporting the informal caregiver. These originate from both the input from stakeholders (*result from development steps 2, 4, 5 and 6)* and evidence-based practices from the literature (*result from development step 2*). The intended client outcomes of the programme are reducing (I)ADL disability, improving self-management skills, increasing QoL of both the client and informal caregiver and reducing healthcare utilisation and expenditures (proximal outcomes), which are common outcome measures in reablement programmes abroad. Eventually, improving these proximal outcomes would help the older adults to remain living at home independently and avoid unnecessary transitions to institutional care (distal outcomes). Figure 2 presents the logic model.

[Insert Figure 2 here]

Translation to practice: description of the I-MANAGE programme

The I-MANAGE programme, as described here, is the result of input from stakeholders and literature research during the last step of the development process (*step 7*) [41]. The following sections describe

For peer review only - http://bmjopen.bmj.com/site/about/guidelines.xhtml

the programme in detail. The programme, as described, may serve as a model for the use in different local settings and leaves room for tailoring to the specific needs and resources of the organisation.

To achieve the intended outcomes presented in the logic model, the programme should intend to apply a two-tiered approach: 1) focusing on the capabilities of the client and 2) paying attention to environmental factors (i.e. physical, social, and organisational). I-MANAGE should be intended for community-dwelling older adults who are still able to (re)learn new skills and apply them in daily life without an indication of terminal illness or planned nursing home admission. The average duration of the programme is preferably 8 weeks according to the members of the working groups.

Interdisciplinary collaboration

I-MANAGE aims to facilitate interdisciplinary collaboration by encouraging intensive collaboration between the reablement team, client, informal caregiver and other care professionals delivering care and support to the client and informal caregiver. The programme should be delivered by an interdisciplinary reablement team, generally consisting of an RN, an OT, and, depending on the local context, supplemented with a PT, social worker, DSW, or other disciplines. The reablement team should initiate the programme and is responsible for the direct support of the client and informal caregiver. A care coordinator should be appointed, which in most cases, is the OT. Depending on the necessary care and goals set by the client, the reablement team may consult other care professionals (e.g. usual home care team, psychologist, or general practitioner); for example, during the intake phase for advice or while working on the client's goals. They should be informed and coached by the reablement team.

Furthermore, regular team meetings should be organised (e.g. (bi-)weekly to discuss the intake of new clients and informal caregivers, the progress made by clients, and the final evaluation of clients' personal goals. Additionally, team meetings could provide the opportunity to discuss specific cases with other team members and to organise training. All members of the reablement team should have access to access the care plan, report progress, and follow up on each other's work,

For peer review only - http://bmjopen.bmj.com/site/about/guidelines.xhtml

preferably through a shared electronic care file (ECF) of the client. Lastly, when discharged from another care facility (e.g. hospital or rehabilitation facility), a smooth handover of client information should be initiated so no necessary details of the client's care process are lost.

Practice-oriented training

Preceding the programme, the reablement team should receive practice-oriented training focusing on adequately delivering I-MANAGE. Within this training, care professionals should receive a detailed manual beforehand, consisting of background information and a description of the programme, including the goal, key components, and I-MANAGE's care process. The manual was developed by the researchers and revised by working group members to make sure it is suitable for practice. Additionally, a toolkit for achieving individual client goals should be provided (e.g. exercise booklet based on the OTAGO exercise programme [45], social map including welfare initiatives, and tools to assess needs and wishes of the caregiver). The training should consist of multiple sessions, preferable in the following structure; the first is a kick-off meeting for the reablement team, focusing on the goal and content of I-MANAGE. Secondly, specific training sessions occur for OT and PT concerning the use of the Canadian Occupational Performance Measure (COPM) [46] to set personal goals with the client and the OTAGO exercise booklet respectively. Finally, the reablement team receives a booster session to practice and discuss challenging situations and focus on motivational and conversation skills. The kick-off and booster sessions each last two hours and the specific sessions for OT and PT last one hour. Additionally, the programme should stimulate training on the job, meaning that members of the reablement team can coach each other and external care professionals based on their own expertise.

I-MANAGE care process

For peer review only - http://bmjopen.bmj.com/site/about/guidelines.xhtml

The I-MANAGE care process consists of five consecutive phases: 1) initiation; 2) intake; 3) care plan; 4) care delivery; and 5) evaluation. Each phase is described in detail below and presented in Figure 3. The five phases are a result of the practical translation of the previously described logic model.

Phase 1: Initiation

Programme referrals could be done through community nurses, general practitioners, or institutional care facilities. Community-dwelling older adults eligible for I-MANAGE should receive information about the programme. The care coordinator has to plan a first visit to present the programme to the client and, if applicable, their informal caregiver to provide the opportunity to ask additional questions. The care coordinator should initiate an exploratory conversation based on the principles of positive health to gain insight into the client's needs and wishes [47].

Phase 2: Intake

Within the first week after the initiation phase, the OT should perform an environmental assessment, identifying necessary home modifications and assistive devices to ensure a safe environment. The environmental assessment is not limited to the inside environment but does also include the entrance and outside environment. Additionally, the care coordinator, or an assigned social worker, should perform an intake with the informal caregiver assessing their needs and wishes. Furthermore, the OT must set meaningful goals with the client using COPM [46] (maximum of five goals). This instrument requires the clients to score both their performance and satisfaction when performing these activities [46]. Goal setting is guided by the exploratory conversation held in phase 1. The ultimate goal is to improve the client's participation and wellbeing; therefore, goals should not merely be (I)ADL-related (e.g. meeting friends at the local café, or painting in the hobby room on the first floor). Goals should be recorded in the client's ECF.

Phase 3: Care plan

For peer review only - http://bmjopen.bmj.com/site/about/guidelines.xhtml

Possible interventions and actions to achieve the client's goals and provide the informal caregiver with the right support should be discussed within the reablement team. These interventions and actions are derived from the available toolkit. Afterwards, the care coordinator should determine the final interventions and actions with the client and informal caregiver, guided by their preferences and capabilities, possibilities of the social network and the physical environment. Subsequently, the care plan should be recorded in the client's ECF and shared with all members of the reablement team. The intensity of the programme depends on the care needs of the client and their pre-set goals, which may require a higher intensity in the beginning but less at the end when (sub-)goals are (partly) reached.

Phase 4: Care delivery

The reablement team should deliver care and support as described in the care plan. Care delivery should be coordinated by the OT, who is also responsible for assisting with (re)learning and practicing meaningful daily activities. The RN is responsible for supporting managing the client's personal care needs. The PT, when part of the reablement team, is responsible for functional training and stimulating participation in daily life. The client's environment should be adapted (e.g. home modifications, assistive devices, and care technology), to support them in (re)learning activities. While supporting meaningful activities, the client's self-management should be stimulated by practicing activities and ensuring that tasks are not taken over by care professionals or informal caregivers. If necessary, a social worker should provide the informal caregiver with additional support to decrease their burden (e.g. respite care).

Phase 5: Evaluation

The evaluation of the care process should be structurally embedded in interdisciplinary team meetings. Based on conclusions drawn during these meetings, the care coordinator can decide whether the care plan is continued, adjusted or terminated. This must be discussed with the client

For peer review only - http://bmjopen.bmj.com/site/about/guidelines.xhtml

and informal caregiver. At the end of the programme, a formal evaluation of the client's goals should take place using COPM [46], including scoring the performance and satisfaction within activities. Afterwards, the care coordinator decides with the client and informal caregiver if the programme should be extended (max. two weeks) to ensure all goals are sufficiently reached or if the client needs referral to usual care.

[Insert Figure 3 here]

For peer review only

For peer review only - http://bmjopen.bmj.com/site/about/guidelines.xhtml

DISCUSSION

This manuscript describes the development and content of I-MANAGE, a community-based reablement programme. The programme was developed for community-dwelling older adults to improve their self-management and participation in daily life and ensure that they can remain living at home independently as long as possible and avoid unnecessary transitions to institutional care, while also increasing QoL and decreasing informal caregiver burden.

The programme contains several key elements that are considered essential and should be present when implementing the programme in any care setting. Firstly, in line with the conceptual definition of reablement [18], interdisciplinary collaboration is important in I-MANAGE. However, how this element is implemented in practice depends on the contextual circumstances of a country or region and the resources available. In this study it was recommended by Dutch stakeholder to appoint a care coordinator, schedule (bi-)weekly meetings, and implement a shared ECF. Recent literature indicates that investing in interdisciplinary collaboration stimulates patient-centred care by ensuring a holistic view of the client's situation and creates shared responsibility [48-50]. This is achieved by good communication and coordination within the team but also with the client and informal caregiver [51]. Moreover, delivery of the programme by an interdisciplinary team, including allied health, such as OTs, is deemed valuable because of their educational background [30, 52]. It also stimulates continuous learning and is experienced as exciting and constructive by care professionals [50, 51]. Additionally, the integrated practice-oriented and on the job training, where care professionals can learn from other disciplines, help to invest in the self-efficacy of care professionals. This is essential, because successfully changing behaviours remains a challenge. The training entails several key topics as mentioned before, however, depending on the local context the extend of the training may vary, for example, due to previously received education or training. Secondly, at the start of the programme (phase 1 and 2), we implemented a standardised goal-setting tool, COPM [46], preceded by an extensive intake based on the principles of positive health [47]. Previous research has indicated that using standardised assessment or goal-setting tools could increase the effectiveness of reablement

For peer review only - http://bmjopen.bmj.com/site/about/guidelines.xhtml

interventions, and is therefore considered an essential element of the programme [30]. Additionally, it increased client involvement and helps professionals to identify meaningful activities with the client [53]. Thirdly, when delivering care (phase 4), the programme integrates several important aspects. Firstly, supporting informal caregivers is assumed to contribute to the effectiveness of I-MANAGE. Previous research found that providing informal caregivers with the right psychosocial and educational support strengthens their ability to cope with their new role [54, 55]. This is an important addition to most reablement programmes because, although making use of the clients' social network is a strategy to reach their goals according to the internationally accepted definition [18], this is often overlooked [56, 57]. Additionally, I-MANAGE promotes optimal use of the social and physical environment, which is essential since a demanding environment can either stimulate or hinder a person's participation in meaningful activities [3, 58]. Lastly, the programme stimulates self-management through participation in meaningful daily activities, which is a core element within reablement ('doing with…' rather than 'doing for…' the client) [18, 59].

The described programme serves as a model and leaves room to tailor the intervention to a specific context and the needs of the organisation. For example, the programme leaves room for the organisation to choose which target group would benefit most. Additionally, delivering the programme by an interdisciplinary team is a prerequisite. However, depending on available resources, the organisation can decide on the composition of the reablement team. Moreover, I-MANAGE is a multi-component programme in which organisations can integrate their own innovative practices, especially when working towards and reaching clients' meaningful goals. Also, the duration and intensity of the programme may vary according to the needs of the local context and chosen target population. Moe and Brinchmann [25] confirmed the necessity of tailoring reablement services to local conditions by arguing that establishing reablement in an existing organisational structure is a complex process. Apart from available resources, during the co-creation process, some influencing factors for implementation were identified. For example, different IT systems, which make communication and information transfer less evident. Moe and Brinchmann [25] mentioned that, next

For peer review only - http://bmjopen.bmj.com/site/about/guidelines.xhtml

to communication and IT systems, habitual ways of offering health services, a lack of knowledge about the rehabilitation potential of older adults and active ageing benefits are also experienced as barriers. A recent scoping review identified several factors that act as barriers and facilitators during the implementation of care innovations, among which, available resources and communication were listed as possible barriers to implementation [60]. It is critical to consider these factors when implementing I-MANAGE in practice. In order to overcome several barriers to the implementation of I-MANAGE, it could be useful to set up advisory boards and working groups to discuss the implementation process and adjust where needed. Additionally, investing in getting the management of care organisations on board to support the implementation within their organisation and setting up knowledge exchange between sites where reablement is being implemented to share experiences and best practices could be beneficial. Investing in a suitable organisational structure is essential because it remains a challenge to successfully change existing organisational structures [25, 61, 62]. Regarding future research, further knowledge is needed to explore feasibility and (cost-)effectiveness of I-MANAGE, as it has not yet been proven. Since the programme is very context specific and can be tailored according to the needs and resources of an organisation, it would be beneficial to investigate what works for which target group and under what circumstances, for example, by means of a realist evaluation [63]. The programme is currently being implemented and evaluated at different Dutch care providers. The systematic reporting of the development of the programme provides useful insight for future research looking to develop complex health interventions or to implement a similar programme.

A strength of I-MANAGE and its development is that it is both theory and evidence-based [42, 43], which has proven to be advantageous when developing effective interventions [64, 65]. Moreover, Thuesen et al. [66] highlight the demand for making theory explicit in reablement interventions and in addressing the physical, psychological, and sociocultural perspectives of ageing within these interventions. Another strength of the development process was the co-creation process, which gave a voice to multiple stakeholders and made the programme suitable for practice. We used

For peer review only - http://bmjopen.bmj.com/site/about/guidelines.xhtml

the development approach described by Bleijenberg et al. [41], which combined a range of published approaches to intervention development to enrich the MRC framework. This approach was chosen because using the MRC framework further assists us in evaluating and adapting the programme. However, we are aware that multiple approaches to intervention development exist as described by O'Cathain et al. [67]. These different approaches share many similarities (e.g. stepwise approach or involvement of stakeholders), but there are also significant differences (e.g. the focus on implementation or theory). It is important to acknowledge these differences and always chose an approach best suited to the purpose of the research. Additionally, most of these approaches are not set in stone and leave room for the researchers own interpretation. It must also fit the setting and timing of the development process. There are also some limitations related to this co-creation process. Firstly, during the process, we always informed management to obtain their support, but they were not included in the working groups, which could have blocked important insight. Secondly, we aimed to include all end users of the programme during the development process (care professionals, clients, and informal caregivers). However, we only included client and informal caregiver representatives in the working groups and interviewed only two informal caregivers between sessions. Additionally, care professionals were recruited from the professional network of researchers and were recruited in a convenient way. We therefore did not ensure variation among these participants in terms of, for example, gender, age, or years of experience. Lastly, we obtained data from many different sources (i.e. scientific literature, individual interviews, working groups, and observations), which made it difficult to find common ground throughout all the sources and forced compromise. However, this use of data triangulation is also a strength of the development of the programme as findings could be checked multiple times with different sources, increasing the validity of the results.

For peer review only - http://bmjopen.bmj.com/site/about/guidelines.xhtml

FIGURE LEGENDS

Figure 1: The development process over time. This figure presents the development process over time from September 2019 until June 2021. The whole process is guided by scientific literature. The figure presents the two parts of the development process each with their related activities. The dark grey rectangles present the (intermediate) result of each part of the process. The number in between brackets represent the seven steps of the development phase as described by Bleijenberg et al. [41]. PT = physiotherapist; GR = geriatric rehabilitation; DSW = domestic support worker; RN = registered nurse.

Figure 2: Logic model of the I-MANAGE programme. The logic model of the I-MANAGE programme presents the aim and associated sub-aims of the programme, the programme components, and proximal and distal outcomes.

Figure 3: Detailed presentation of the I-MANAGE care process. I-MANAGE has a 5-phase care process (initiation, intake, care plan, care delivery, and evaluation), preceded with the referral of the client through different routes. After an average duration of 8 weeks, clients are referred to aftercare. Interdisciplinary collaboration is a continuous element of the programme.

ETHICS APPROVAL AND CONSENT TO PARTICIPATE

As participants were not subjected to actions and no rules of behaviour were imposed, no ethical approval was needed according to the Dutch 'Medical Research Involving Human Subjects Act' (WMO). Nevertheless, the study was carried out in accordance with the principles of ethical research and all participants involved in the working group sessions and interviews provided either written or verbal informed consent prior to the start of each session.

AVAILABILITY OF DATA AND MATERIALS

For peer review only - http://bmjopen.bmj.com/site/about/guidelines.xhtml

The datasets used and/or analysed during the current study available from the corresponding author on reasonable request.

COMPETING INTEREST

The authors declare that they have no competing interests

FUNDING

This work was supported by the European Union's Horizon 2020 research and innovation programme under the Marie Skłodowska-Curie grant agreement No 812656 as part of the TRANS-SENIOR project (www.trans-senior.eu).

AUTHORS' CONTRIBUTORS

All authors – IM, HV, GIJMK, JCMvH, EV, GG, SFM – were involved in the development of the I-MANAGE programme. IM wrote the first draft of the article. All authors reviewed the manuscript and approved the final version.

ACKNOWLEDGEMENTS

We would like to thank the Dutch stakeholders who have participated in the working group sessions, observations, and individual interviews, and therefore have helped with the development of a reablement programme suitable for the Dutch home care setting: Angelica de Lang, Nelly Jacobs, Jose Blezer, Belkis Bozdemir, Marcel Mannens, Marc Lowis, Maud Scholtes, Mandy Boosten, Hella Senden, Angèle van Kasteren, Anne Gulpers, Eveline van Velthuijsen, Anouschka Feenstra, Elfi Pakbeers, Nadine Klinkers, Marjo Gartener (MeanderGroep Zuid-Limburg), Ewout Smit (Vivium Zorggroep), Teuni Rooijackers, Angelique de Rijk (Maastricht University), Bianca Miltenburg (Cicero Zorggroep), Marie-Therese Bindels, Femke Scheuerman (gemeente Kerkrade), Marja Veenstra (Burgerkracht Limburg).

For peer review only - http://bmjopen.bmj.com/site/about/guidelines.xhtml

SUPPLEMENTAL MATERIAL

Supplementary file 1: GUIDED checklist. Completed guideline for reporting for intervention development studies.

Supplementary file 2: Overview of the different stakeholders involved per research activity.

Supplementary file 3: Overview of the intermediate results of the first five steps of the development phase.

For peer review only

REFERENCES

1. Zingmark M, Norström F. Transitions between levels of dependency among older people receiving social care – a retrospective longitudinal cohort study in a Swedish municipality. BMC Geriatr. 2021 2021/06/02;21(1):342.

2. Brown GC. Living too long: the current focus of medical research on increasing the quantity, rather than the quality, of life is damaging our health and harming the economy. EMBO Rep. 2015;16(2):137-41.

3. Rustad EC, Furnes B, Cronfalk BS, et al. Older patients' experiences during care transition. Patient Prefer Adherence. 2016;10:769-79.

4. Wahl HW, Fange A, Oswald F, et al. The home environment and disability-related outcomes in aging individuals: What is the empirical evidence? Gerontologist. 2009 Jun;49(3):355-67.

5. Arnau A, Espaulella J, Serrarols M, et al. Risk factors for functional decline in a population aged 75 years and older without total dependence: A one-year follow-up. Arch Gerontol Geriatr. 2016;65:239-47.

6. Tappenden P, Campbell F, Rawdin A, et al. The clinical effectiveness and cost-effectiveness of home-based, nurse-led health promotion for older people: A systematic review. Health Technol Assess. 2012;16(20):1-72.

7. Fujisawa R, Colombo F. The Long-Term Care Workforce: Overview and Strategies to Adapt Supply to a Growing Demand. 2009.

8. Arbaje AI, Kansagara DL, Salanitro AH, et al. Regardless of age: Incorporating principles from geriatric medicine to improve care transitions for patients with complex needs. J Gen Intern Med. 2014;29(6):932-9.

9. Koopmans RT, Lavrijsen JC, Hoek JF, et al. Dutch elderly care physician: a new generation of nursing home physician specialists. J Am Geriatr Soc. 2010;58(9):1807-9.

10. Nicholson C, Meyer J, Flatley M, et al. The experience of living at home with frailty in old age: A psychosocial qualitative study. Int J Nurs Stud. 2013 2013/09/01/;50(9):1172-9.

11. Aspinal F, Glasby J, Rostgaard T, et al. New horizons: Reablement - supporting older people towards independence. Age Ageing. 2016;45(5):572-6.

12. Beard JR, Officer A, de Carvalho IA, et al. The World report on ageing and health: a policy framework for healthy ageing. The Lancet. 2016;387(10033):2145-54.

13. Ferrah N, Ibrahim JE, Kipsaina C, et al. Death Following Recent Admission Into Nursing Home From Community Living: A Systematic Review Into the Transition Process. J Aging Health. 2018;30(4):584-604.

For peer review only - http://bmjopen.bmj.com/site/about/guidelines.xhtml

14. Oliver D, Foot C, Humphries R. Making our health and care systems fit for an ageing population: King's Fund London: UK; 2014.

15. Wiles JL, Leibing A, Guberman N, et al. The meaning of "aging in place" to older people. Gerontologist. 2012 Jun;52(3):357-66.

16. Cesari M, Vellas B, Hsu F-C, et al. A physical activity intervention to treat the frailty syndrome in older persons-results from the LIFE-P study. J Gerontol A Biol Sci Med Sci. 2015;70(2):216-22.

17. Daniels R, Metzelthin SF, van Rossum E, et al. Interventions to prevent disability in frail community-dwelling older persons: An overview. Eur J Ageing. 2010;7(1):37-55.

18. Metzelthin SF, Rostgaard T, Parsons M, et al. Development of an internationally accepted definition of reablement: a Delphi study. Ageing and Society. 2022;42(3):703-18.

19. Doh D, Smith R, Gevers P. Reviewing the reablement approach to caring for older people. Ageing and Society. 2019:1-13.

20. Metzelthin SF, Zijlstra GA, van Rossum E, et al. 'Doing with ...' rather than 'doing for ...' older adults: rationale and content of the 'Stay Active at Home' programme. Clin Rehabil. 2017;31(11):1419-30.

21. Tessier A, Beaulieu M-D, McGinn CA, et al. Effectiveness of Reablement: A Systematic Review. Healthcare policy. 2016;11(4):49-59.

22. Whitehead PJ, Worthington EJ, Parry RH, et al. Interventions to reduce dependency in personal activities of daily living in community dwelling adults who use homecare services: A systematic review. Clin Rehabil. 2015 Nov;29(11):1064-76.

23. Resnick B, Galik E, Boltz M. Function focused care approaches: Literature review of progress and future possibilities. J Am Med Dir Assoc. 2013 2013/05/01/;14(5):313-8.

24. Parsons J, Burton E, Graff L, et al. 4: Reablement as an evolution in home care: a comparison of implementation across five countries. In: Rostgaard T, Parsons J, Tuntland H, editors. Reablement in Long-Term Care for Older People: International Perspectives and Future Directions. Bristol: Policy Press; 2023. p. 68-92.

25. Moe C, Brinchmann BS. Tailoring reablement: A grounded theory study of establishing reablement in a community setting in Norway. Health Soc Care Community. 2018 Jan;26(1):113-21.

26. Wess T, Steiner W, Dür M, et al. Reablement – relevant factors for implementation: an exploratory sequential mixed-methods study design. BMC Health Serv Res. 2022 2022/07/28;22(1):959.

27. Rostgaard T, Tuntland H, Parsons J. 1: Introduction: The concept, rationale, and implications of reablement. In: Rostgaard T, Parsons J, Tuntland H, editors. Reablement in Long-Term Care for Older People: International Perspectives and Future Directions. Bristol: Policy Press; 2023. p. 3-20.

For peer review only

28. Mjøsund HL, Moe CF, Burton E, et al. Integration of Physical Activity in Reablement for Community Dwelling Older Adults: A Systematic Scoping Review. J Multidiscip Healthc. 2020;13:1291-315.

29. Duncan E, Cathain A, Rousseau N, et al. Guidance for reporting intervention development studies in health research (GUIDED): an evidence-based consensus study. BMJ Open. 2020;10(4):e033516.

30. Buma LE, Vluggen S, Zwakhalen S, et al. Effects on clients' daily functioning and common features of reablement interventions: a systematic literature review. Eur J Ageing. 2022 2022/05/03.

31. Assander S, Bergström A, Eriksson C, et al. ASSIST: a reablement program for older adults in Sweden – a feasibility study BMC Geriatr. 2022 2022/07/26;22(1):618.

32. Bergström A, Borell L, Meijer S, et al. Evaluation of an intervention addressing a reablement programme for older, community-dwelling persons in Sweden (ASSIST 1.0): a protocol for a feasibility study. BMJ Open. 2019;9(7):e025870.

33. Zingmark M, Kylén M. Feasibility of a reablement-program in a Swedish municipality. Scand J Occup Ther. 2023 2023/01/02;30(1):53-64.

34. Hoffmann TC, Glasziou PP, Boutron I, et al. Better reporting of interventions: template for intervention description and replication (TIDieR) checklist and guide. BMJ : British Medical Journal. 2014;348:g1687.

35. Craig P, Dieppe P, Macintyre S, et al. Developing and evaluating complex interventions: the new Medical Research Council guidance. BMJ. 2008;337:a1655.

36. Skivington K, Matthews L, Simpson SA, et al. A new framework for developing and evaluating complex interventions: update of Medical Research Council guidance. BMJ. 2021;374:n2061.

37. Bigonnesse C, Chaudhury H. The Landscape of "Aging in Place" in Gerontology Literature: Emergence, Theoretical Perspectives, and Influencing Factors. Journal of Aging and Environment. 2020 2020/07/02;34(3):233-51.

38. Kroneman M, Boerma W, van den Berg M, et al. Netherlands: Health System Review. Health Syst Transit. 2016 Mar;18(2):1-240.

39. Genet N, Boerma W, Kroneman M, et al. Home Care Across Europe. Current Structure and Future Challenges2013.

40. Centers for Disease Control and Prevention. Evaluation guide. Developing and using a logic model. CDC Division for Heart Disease and Stroke Prevention.

41. Bleijenberg N, de Man-van Ginkel JM, Trappenburg JCA, et al. Increasing value and reducing waste by optimizing the development of complex interventions: Enriching the development phase of the Medical Research Council (MRC) Framework. Int J Nurs Stud. 2018;79:86-93.

For peer review only - http://bmjopen.bmj.com/site/about/guidelines.xhtml

42. Mouchaers I, Van Haastregt JCM, Verbeek H, et al. « Reablement » comme outil thérapeutique pour offrir aux personnes âgées une vie de qualité à domicile après une réadaptation gériatrique. Rev Med Brux. 2021;42(4):324-32.

43. Mouchaers I, Verbeek H, Kempen GIJM, et al. The concept of disability and its causal mechanisms in older people over time from a theoretical perspective: a literature review. Eur J Ageing. 2022 2022/01/29.

44. Braun V, Clarke V. Using thematic analysis in psychology. Qualitative Research in Psychology. 2006 2006/01/01;3(2):77-101.

45. Campbell AJ, Robertson MC. Het Otago Oefenprogramma om valincidenten bij ouderen te voorkomen. Amersfoort: Nederlandse Vereniging voor Fysiotherapie in de Geriatrie; 2013.

46. Law M, Baptiste S, McColl M, et al. The Canadian Occupational Performance Measure: An Outcome Measure for Occupational Therapy. Canadian Journal of Occupational Therapy. 1990;57(2):82-7.

47. Huber M, van Vliet M, Giezenberg M, et al. Towards a 'patient-centred' operationalisation of the new dynamic concept of health: a mixed methods study. BMJ Open. 2016;6(1):e010091.

48. Schmutz JB, Meier LL, Manser T. How effective is teamwork really? The relationship between teamwork and performance in healthcare teams: a systematic review and meta-analysis. BMJ Open. 2019;9(9):e028280.

49. Ebrahimi Z, Patel H, Wijk H, et al. A systematic review on implementation of person-centered care interventions for older people in out-of-hospital settings. Geriatric Nursing. 2021 2021/01/01/;42(1):213-24.

50. Eliassen M, Moholt JM. Boundary work in task-shifting practices - a qualitative study of reablement teams. Physiother Theory Pract. 2022 Apr 18:1-14.

51. Birkeland A, Tuntland H, Førland O, et al. Interdisciplinary collaboration in reablement - a qualitative study. J Multidiscip Healthc. 2017;10:195-203.

52. Reeves S, Fletcher S, Barr H, et al. A BEME systematic review of the effects of interprofessional education: BEME Guide No. 39. Med Teach. 2016 Jul;38(7):656-68.

53. Rose A, Rosewilliam S, Soundy A. Shared decision making within goal setting in rehabilitation settings: A systematic review. Patient Educ Couns. 2017 Jan;100(1):65-75.

54. Lopez-Hartmann M, Wens J, Verhoeven V, et al. The effect of caregiver support interventions for informal caregivers of community-dwelling frail elderly: a systematic review. Int J Integr Care. 2012 Jul-Sep;12:e133.

For peer review only

For peer review only - http://bmjopen.bmj.com/site/about/guidelines.xhtml

55. Spiers GF, Liddle J, Kunonga TP, et al. What are the consequences of caring for older people and what interventions are effective for supporting unpaid carers? A rapid review of systematic reviews. BMJ Open. 2021;11(9):e046187.

56. Hjelle KM, Alvsvåg H, Førland O. The relatives' voice: how do relatives experience participation in reablement? A qualitative study. Journal of multidisciplinary healthcare. 2016;10:1-11.

57. van Wieringen M, Broese van Groenou MI, Groenewegen P. Impact of home care management on the involvement of informal caregivers by formal caregivers. Home Health Care Serv Q. 2015 Apr-Jun;34(2):67-84.

58. de Vries NM, Staal JB, van Ravensberg CD, et al. Outcome instruments to measure frailty: A systematic review. Ageing Res Rev. 2011 2011/01/01/;10(1):104-14.

59. Clotworthy A, Kusumastuti S, Westendorp RGJ. Reablement through time and space: a scoping review of how the concept of 'reablement' for older people has been defined and operationalised. BMC Geriatr. 2021 2021/01/15;21(1):61.

60. Fakha A, Groenvynck L, de Boer B, et al. A myriad of factors influencing the implementation of transitional care innovations: a scoping review. Implementation Science. 2021 2021/02/26;16(1):21.

61. Reay T, Goodrick E, D'Aunno T. Health Care Research and Organization Theory. Cambridge: Cambridge University Press; 2021. Available from: https://www.cambridge.org/core/elements/health-care-research-and-organization-theory/18F375C5D5DEBE92B725FAB547EA076D.

62. Rooijackers T, van Rossum E, Zijlstra GAR, et al. Effectiveness of a reablement training program on self-efficacy and outcome expectations regarding client activation in homecare staff: A cluster randomized controlled trial. Geriatric Nursing. 2022 2022/01/01/;43:104-12.

63. Pawson R, Tilley N. Realistic evaluation. London: Sage; 1997.

64. Michie S, Prestwich A. Are interventions theory-based? Development of a theory coding scheme. Health Psychol. 2010 Jan;29(1):1-8.

65. Bartholomew LK, Mullen PD. Five roles for using theory and evidence in the design and testing of behavior change interventions. J Public Health Dent. 2011;71(s1):S20-S33.

66. Thuesen J, Feiring M, Doh D, et al. Reablement in need of theories of ageing: would theories of successful ageing do? Ageing and Society. 2021:1-13.

67. O'Cathain A, Croot L, Sworn K, et al. Taxonomy of approaches to developing interventions to improve health: a systematic methods overview. Pilot and Feasibility Studies. 2019 2019/03/12;5(1):41.

For peer review only

For peer review only - http://bmjopen.bmj.com/site/about/guidelines.xhtml

For peer review only

For peer review only - http://bmjopen.bmj.com/site/about/guidelines.xhtml

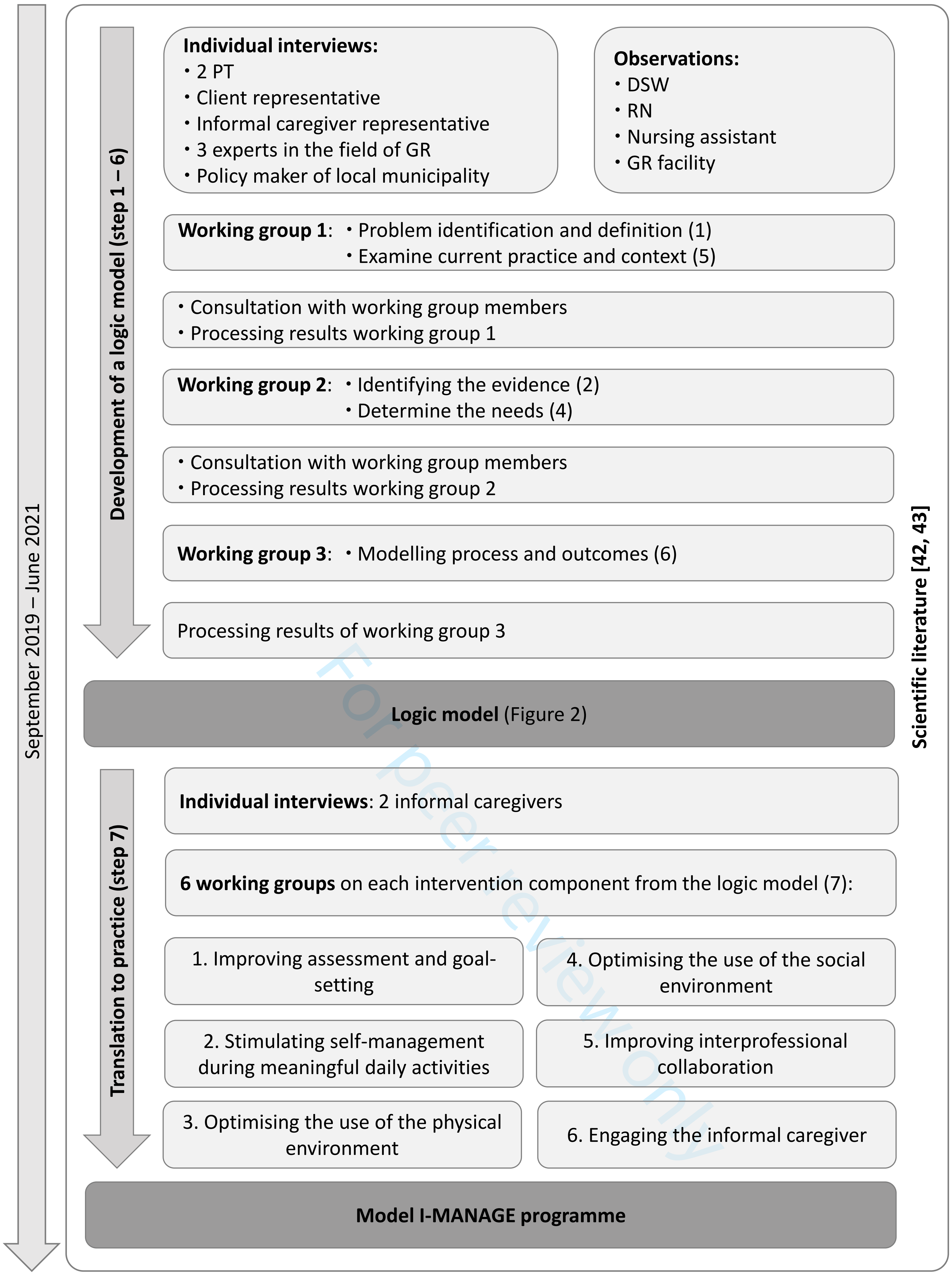

For peer review only

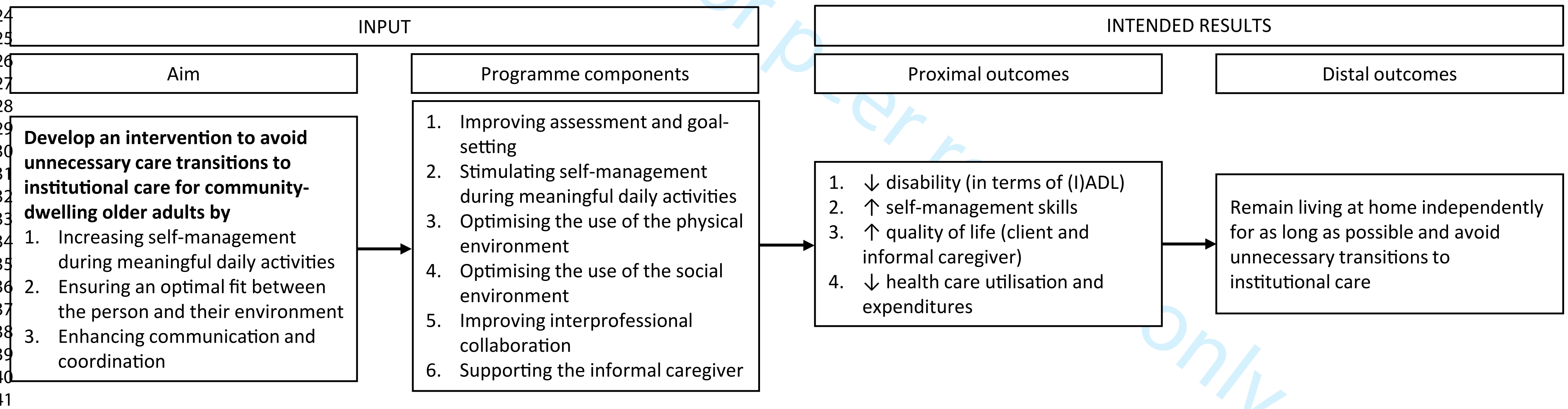

For peer review only

Referral of the client to I-MANAGE programme:
- Community nurses
- General practitioner
- Institutional care facilities

Start of the programme

Phase 1: Initiation
1. Provide information to client (and informal caregiver)
2. First visit by OT
   - Exploratory conversation based on Positive Health
3. If discharged from other care facility; initiate smooth handover

Week 1

Phase 2: Intake
1. Environmental assessment by OT
2. Set meaningful goals by using COPM
   - Guided by exploratory conversation from phase 1
3. Intake with informal caregiver to assess burden and needs

Week 2

Phase 3: Care plan
1. Determine interventions and actions to reach goals
   - Guided by preferences of client/informal caregiver
   - Specific attention for client's capabilities, social network and physical environment
2. Recorded in electronic care file

Week 3-8

Phase 4: Care delivery
1. Care provided as described in care plan
2. Follow-up on the care process by reablement team
3. If needed, initiate additional support for informal caregiver

Week 8

Phase 5: Evaluation
1. Continuous evaluation during care visit and team meetings
   - Continue, adjust or terminate care plan
2. Formal evaluation after 8 weeks by using COPM

Aftercare
1. Continue programme for maximum of 2 weeks
2. Referral to usual care

Interdisciplinary collaboration:
- Appointed care coordinator
- Regular team meetings
- Shared electronic care file
- Coaching on the job

Supplementary file 1. GUIDED checklist. A guideline for reporting for intervention development studies.

**Item description**	**Explanation**	**Page in manuscript where item is located**	**Other***
Report the context for which the intervention was developed.	Understanding the context in which an intervention was developed informs readers about the suitability and transferability of the intervention to the context in which they are considering evaluating, adapting or using the intervention. Context here can include place, organisational and wider socio- political factors that may influence the development and/or delivery of the intervention.	P 8 – 9	
Report the purpose of the intervention development process.	Clearly describing the purpose of the intervention specifies what it sets out to achieve. The purpose may be informed by research priorities, for example those identified in systematic reviews, evidence gaps set out in practice guidance such as The National Institute for Health and Care Excellence or specific prioritisation exercises such as those undertaken with patients and practitioners through the James Lind Alliance.	P 6 – 7	
Report the target population for the intervention development process.	The target population is the population that will potentially benefit from the intervention – this may include patients, clinicians, and/or members of the public. If the target population is clearly described then readers will be able to understand the relevance of the intervention to their own research or practice. Health inequalities, gender and ethnicity are features of the target population that may be relevant to intervention development processes.	P 8 – 9	
Report how any published intervention development approach contributed to the development process.	Many formal intervention development approaches exist and are used to guide the intervention development process (e.g. Squid or The Person Based Approach to Intervention Development). Where a formal intervention development approach is used, it is helpful to describe the process that was followed, including any deviations. More general approaches to intervention development also exist and have been categorised as follows: Target Population-centred intervention development; evidence and theory-based intervention development; partnership intervention development; implementation-based intervention development; efficacy- based intervention development; step or phased-based intervention development; and intervention-specific intervention development. These approaches do not always have specific guidance that describe their use. Nevertheless, it is helpful to give a rich description of how any published approach was operationalised	P 8	

For peer review only

Report how evidence from different sources informed the intervention development process.	Intervention development is often based on published evidence and/or primary data that has been collected to inform the intervention development process. It is useful to describe and reference all forms of evidence and data that have informed the development of the intervention because evidence bases can change rapidly, and to explain the manner in which the evidence and/or data was used. Understanding what evidence was and was not available at the time of intervention development can help readers to assess transferability to their current situation.	P 10	Supplementary file 3 + [1]
Report how/if published theory informed the intervention development process.	Reporting whether and how theory informed the intervention development process aids the reader's understanding of the theoretical rationale that underpins the intervention. Though not mentioned in the e-Delphi or consensus meeting, it became increasingly apparent through the development of our guidance that this theory item could relate to either existing published theory or programme theory.	P 10	Supplementary file 3 + [2]
Report any use of components from existing interventions in the current intervention development process.	Some interventions are developed with components that have been adopted from existing interventions. Clearly identifying components that have been adopted or adapted and acknowledging their original source helps the reader to understand and distinguish between the novel and adopted components of the new intervention.	P 10	Supplementary file 3 + [1]
Report any guiding principles, people or factors that were prioritised when making decisions during the intervention development process.	Reporting any guiding principles that governed the development of the application helps the reader to understand the authors' reasoning behind the decisions that were made. These could include the examples of particular populations who views are being considered when designing the intervention, the modality that is viewed as being most appropriate, design features considered important for the target population, or the potential for the intervention to be scaled up.	P 9 – 11	Supplementary file 3
Report how stakeholders contributed to the intervention development process.	Potential stakeholders can include patient and community representatives, local and national policy makers, health care providers and those paying for or commissioning health care. Each of these groups may influence the intervention development process in different ways. Specifying how differing groups of stakeholders contributed to the intervention development process helps the reader to understand how stakeholders were involved and the degree of influence they had on the overall process. Further detail on how to integrate stakeholder contributions within intervention reporting are available.	P 9 – 11	
Report how the intervention	Intervention development is frequently an iterative process. The conclusion of the initial	P 13 – 18	

For peer review only

changed in content and format from the start of the intervention development process.	phase of intervention development does not necessarily mean that all uncertainties have been addressed. It is helpful to list remaining uncertainties such as the intervention intensity, mode of delivery, materials, procedures, or type of location that the intervention is most suitable for. This can guide other researchers to potential future areas of research and practitioners about uncertainties relevant to their healthcare context.		
Report any changes to the intervention required or likely to be required for subgroups.	Specifying any changes that the intervention development team perceive are required for the intervention to be delivered or tailored to specific sub groups enables readers to understand the applicability of the intervention to their target population or context. These changes could include changes to personnel delivering the intervention, to the content of the intervention, or to the mode of delivery of the intervention.	P 20 – 21	
Report important uncertainties at the end of the development process.	Intervention development is frequently an iterative process. The conclusion of the initial phase of intervention development does not necessarily mean that all uncertainties have been addressed. It is helpful to list remaining uncertainties such as the intervention intensity, mode of delivery, materials, procedures, or type of location that the intervention is most suitable for. This can guide other researchers to potential future areas of research and practitioners about uncertainties relevant to their healthcare context.	P 21 – 22	
Follow TIDieR guidance when describing the developed intervention.	Interventions have been poorly reported for a number of years. In response to this, internationally recognized guidance has been published to support the high quality reporting of health care interventions and public health interventions. This guidance should therefore be followed when describing a developed intervention.		
Brief name.	Provide the name or a phrase that describes the intervention.	P 1	Title page
Rationale, theory and aim of the elements essential to the intervention.	Describe any rationale, theory, or goal of the elements essential to the intervention.	P 13	Supplementary file 3 + [2]
Materials used in the intervention delivery or in the training of intervention providers.	Describe any physical or informational materials used in the intervention, including those provided to participants or used in intervention delivery or in training of intervention providers. Provide information on where the materials can be accessed (e.g. online appendix, URL).	P 13 – 18	
Procedures, activities and/or processes used including enabling	Describe each of the procedures, activities, and/or processes used in the intervention, including any enabling or support activities.	P 13 – 18	

For peer review only

or support activities.			
Expertise and background of intervention providers.	For each category of intervention provider (e.g. psychologist, nursing assistant), describe their expertise, background and any specific training given.	P 14 – 18	
How the intervention was provided.	Describe the modes of delivery (e.g. face-to-face or by some other mechanism, such as internet or telephone) of the intervention and whether it was provided individually or in a group.	P 13 – 18	
Location where the intervention occurred (incl. necessary infrastructure or relevant features).	Describe the type(s) of location(s) where the intervention occurred, including any necessary infrastructure or relevant features.	P 8 – 9	
When and how much the intervention was delivered.	Describe the number of times the intervention was delivered and over what period of time including the number of sessions, their schedule, and their duration, intensity or dose.	P 13 – 18	
Tailoring – what, why, when and how.	If the intervention was planned to be personalised, titrated or adapted, then describe what, why, when, and how.	P 20 – 21	
Modifications. †	If the intervention was modified during the course of the study, describe the changes (what, why, when, and how).	N/A	
Strategies to improve or maintain fidelity.	If intervention adherence or fidelity was assessed, describe how and by whom, and if any strategies were used to maintain or improve fidelity, describe them.	P 20 – 21	
Adherence and fidelity.†	If intervention adherence or fidelity was assessed, describe the extent to which the intervention was delivered as planned.	N/A	
Report the intervention development process in an open access format.	Unless reports of intervention development are available people considering using an intervention cannot understand the process that was undertaken and make a judgement about its appropriateness to their context. It also limits cumulative learning about intervention development methodology and observed consequences at later evaluation, translation and implementation stages. Reporting intervention development in an open access (Gold or Green) publishing format increases the accessibility and visibility of intervention development research and makes it more likely to be read and used. Potential platforms for open access publication of intervention development include open access journal publications, freely accessible funder reports or a study web-page that details the intervention development process.		Aim of this manuscript

Note. * e.g. if item is reported elsewhere, then the location of this information can be stated here. † If completing the TIDieR checklist for a protocol, these items are not relevant to the protocol and cannot be described until the study is complete.

For peer review only

References

1. Mouchaers I, Van Haastregt JCM, Verbeek H, et al. « Reablement » comme outil thérapeutique pour offrir aux personnes âgées une vie de qualité à domicile après une réadaptation gériatrique. Rev Med Brux. 2021;42(4):324-32.

2. Mouchaers I, Verbeek H, Kempen GIJM, et al. The concept of disability and its causal mechanisms in older people over time from a theoretical perspective: a literature review. Eur J Ageing. 2022 2022/01/29.

For peer review only

Supplementary file 2. Overview of the different stakeholders involved per research activity.

Stakeholders[a] (n = 26)	Observations[b]	Individual interviews	Working groups: Development of a logic model[c]			Working groups: Translation to practice[d]					
			1	2	3	1	2	3	4	5	6
Domestic support worker	•		•	•	•		•			•	
Registered nurse (n = 3)	•		•	•	•	•	•		•	•	
Nursing assistant	•										
Physiotherapist (n = 4)		•	•	•	•	•				•	
Expert in the field of GR research (n = 3)		•	•								
Informal caregiver (n = 3)		•									•
Policy maker of the local municipality (n = 2)		•		•	•			•	•		
Client representative		•	•	•					•		
Informal caregiver representative		•	•	•	•						
Occupational therapist (n = 2)			•	•	•	•	•	•			
Psychologist			•	•	•						•
Geriatrician						•					
Reablement researcher							•				
Manager community care team										•	
Consultant informal care									•		•

GR = geriatric rehabilitation

Note: [a] if n > 1, not all stakeholders participated in every indicated research activity for that group; [b] observations were performed by the researchers; [c] indicates the three consecutive working group sessions throughout which the logic model was being developed following step 1 to 6 as described by Bleijenberg et al. [1]; [d] indicated the 6 working groups related to each programme component described in the logic model (1 = improving assessment and goal-setting, 2 = stimulating self-management during meaningful daily activities, 3 = optimising the use of the physical environment, 4 = optimising the use of the social environment, 5 = improving interprofessional collaboration, and 6 = engaging the informal caregiver).

References

1. Bleijenberg N, de Man-van Ginkel JM, Trappenburg JCA, et al. Increasing value and reducing waste by optimizing the development of complex interventions: Enriching the development phase of the Medical Research Council (MRC) Framework. Int J Nurs Stud. 2018;79:86-93.

For peer review only

Supplementary file 3. Overview of the intermediate results of the first five steps of the development phase.

Programme development step	Results
1. Problem identification	The identified problems were situated in three main areas:<br>1. A lack of attention to meaningful daily activities. According to the working group, older persons were mainly focused on functional goals related to (I)ADL tasks rather than meaningful daily activities that increase their well-being. Additionally, the focus of care is often on eliminating diseases and symptoms instead of maintaining quality of life [1, 2];<br>2. There appeared a gap between the person's abilities, needs, and wishes, and the environment they reside in. When older people experience a deterioration in health status, their environment is often not adapted, i.e. the necessary home modifications and assistive devices are not always in place [3, 4];<br>3. Communication and coordination within the care trajectory of an older person. There is insufficient communication amongst different care professionals but also with older persons and informal caregivers [4-7].
2. Identifying the evidence	The identification of the evidence-based practices was mainly based on literature research. A literature review was performed to gain insight into current evidence on effective reablement programmes aimed at improving the independent living of older persons [8]. We specifically sought to gain a general understanding of different reablement programmes, their characteristics (e.g. duration), content, outcome measures, and effectiveness. Thirteen programmes were identified, all intended for (I)ADL-impaired community-dwelling older adults. The most common features were: 1) delivery by a trained and coordinated multidisciplinary team and 2) implementation of an intake assessment and goal-setting tool. The applied interventions focused on supporting (I)ADL and physical functioning, and improving home safety. Nine programmes showed a statistically significant improvement on at least one of the outcome measures (e.g. (I)ADL functioning).
3. Identifying or developing theory	A literature review was performed on the concept of disability and its working mechanisms [9] to identify a solid theoretical foundation for our programme (*step 3*). We concluded that by optimising the use of personal, as well as environmental, resources and focusing on health and functioning, rather than disability, an older person's independence and wellbeing could be improved, especially while performing meaningful daily activities in accordance with the person's needs and preferences.
4. Determine the needs	The determined needs are closely related to the identified problems. Stakeholders expressed the need for:<br>1. Standardised goal-setting at the beginning of the care process. Goals should be meaningful, embedded in the older person's own environment, and not solely focused on (I)ADL;<br>2. Timely assessment and, if necessary, adaptations to the home environment of the older person. This concerns adaptations to the built environment, as well as the availability of assistive devices. Additionally, the social environment of the older person needs to be assessed in order to make optimal use of it or provide additional support if the social network is rather weak;<br>3. Improved communication and coordination within the care trajectory of the older person. For example, a smooth handover of information between care professionals and organisations, involvement of the informal caregiver when discussing a client's care needs, multidisciplinary collaboration with clear roles for each provider, and the appointment of a care coordinator.
5. Examining current practice and context	When examining the current practice and context we focused on positive aspects and possible barriers for implementation. Possible barriers could be different financial arrangements and different communication systems within a multidisciplinary team. Positive aspects include the comprehensive assessment by community nurses at the start

For peer review only

	of receiving community care, a signalling role for domestic support workers, and the presence of various welfare initiatives in the Netherlands. A suggestion was made to include the principles of Positive Health [10] in the programme, a best practice that is already known and applied by some care professionals.

References

1. Nicholson C, Meyer J, Flatley M, et al. The experience of living at home with frailty in old age: A psychosocial qualitative study. Int J Nurs Stud. 2013 2013/09/01/;50(9):1172-9.
2. Aspinal F, Glasby J, Rostgaard T, et al. New horizons: Reablement - supporting older people towards independence. Age Ageing. 2016;45(5):572-6.
3. Q-Consult zorg. Inventarisatie zorginhoud GRZ: Het net opgehaald na zes jaar GRZ in de Zorgverzekeringswet. Q-Consult zorg, 2019.
4. Rustad EC, Furnes B, Cronfalk BS, et al. Older patients' experiences during care transition. Patient Prefer Adherence. 2016;10:769-79.
5. Everink IHJ, van Haastregt JCM, Kempen GIJM, et al. Uitdagingen in de geriatrische revalidatiezorg: de ontwikkeling van een zorgpad. Tijdschr Gerontol Geriatr. 2015 2015/04/01;46(2):104-12.
6. Bauer M, Fitzgerald L, Haesler E, et al. Hospital discharge planning for frail older people and their family. Are we delivering best practice? A review of the evidence. J Clin Nurs. 2009;18(18):2539-46.
7. Adelman RD, Tmanova LL, Delgado D, et al. Caregiver burden: a clinical review. JAMA. 2014;311(10):1052-60.
8. Mouchaers I, Van Haastregt JCM, Verbeek H, et al. « Reablement » comme outil thérapeutique pour offrir aux personnes âgées une vie de qualité à domicile après une réadaptation gériatrique. Rev Med Brux. 2021;42(4):324-32.
9. Mouchaers I, Verbeek H, Kempen GIJM, et al. The concept of disability and its causal mechanisms in older people over time from a theoretical perspective: a literature review. Eur J Ageing. 2022 2022/01/29.
10. Huber M, van Vliet M, Giezenberg M, et al. Towards a ‘patient-centred’ operationalisation of the new dynamic concept of health: a mixed methods study. BMJ Open. 2016;6(1):e010091.

For peer review only

Supplementary file 1. GUIDED checklist. A guideline for reporting for intervention development studies.

**Item description**	**Explanation**	**Page in manuscript where item is located**	**Other***
Report the context for which the intervention was developed.	Understanding the context in which an intervention was developed informs readers about the suitability and transferability of the intervention to the context in which they are considering evaluating, adapting or using the intervention. Context here can include place, organisational and wider socio- political factors that may influence the development and/or delivery of the intervention.	P 8 – 9	
Report the purpose of the intervention development process.	Clearly describing the purpose of the intervention specifies what it sets out to achieve. The purpose may be informed by research priorities, for example those identified in systematic reviews, evidence gaps set out in practice guidance such as The National Institute for Health and Care Excellence or specific prioritisation exercises such as those undertaken with patients and practitioners through the James Lind Alliance.	P 6 – 7	
Report the target population for the intervention development process.	The target population is the population that will potentially benefit from the intervention – this may include patients, clinicians, and/or members of the public. If the target population is clearly described then readers will be able to understand the relevance of the intervention to their own research or practice. Health inequalities, gender and ethnicity are features of the target population that may be relevant to intervention development processes.	P 8 – 9	
Report how any published intervention development approach contributed to the development process.	Many formal intervention development approaches exist and are used to guide the intervention development process (e.g. Squid or The Person Based Approach to Intervention Development). Where a formal intervention development approach is used, it is helpful to describe the process that was followed, including any deviations. More general approaches to intervention development also exist and have been categorised as follows: Target Population-centred intervention development; evidence and theory-based intervention development; partnership intervention development; implementation-based intervention development; efficacy- based intervention development; step or phased-based intervention development; and intervention-specific intervention development. These approaches do not always have specific guidance that describe their use. Nevertheless, it is helpful to give a rich description of how any published approach was operationalised	P 8	

For peer review only

Report how evidence from different sources informed the intervention development process.	Intervention development is often based on published evidence and/or primary data that has been collected to inform the intervention development process. It is useful to describe and reference all forms of evidence and data that have informed the development of the intervention because evidence bases can change rapidly, and to explain the manner in which the evidence and/or data was used. Understanding what evidence was and was not available at the time of intervention development can help readers to assess transferability to their current situation.	P 10	Supplementary file 3 + [1]
Report how/if published theory informed the intervention development process.	Reporting whether and how theory informed the intervention development process aids the reader's understanding of the theoretical rationale that underpins the intervention. Though not mentioned in the e-Delphi or consensus meeting, it became increasingly apparent through the development of our guidance that this theory item could relate to either existing published theory or programme theory.	P 10	Supplementary file 3 + [2]
Report any use of components from existing interventions in the current intervention development process.	Some interventions are developed with components that have been adopted from existing interventions. Clearly identifying components that have been adopted or adapted and acknowledging their original source helps the reader to understand and distinguish between the novel and adopted components of the new intervention.	P 10	Supplementary file 3 + [1]
Report any guiding principles, people or factors that were prioritised when making decisions during the intervention development process.	Reporting any guiding principles that governed the development of the application helps the reader to understand the authors' reasoning behind the decisions that were made. These could include the examples of particular populations who views are being considered when designing the intervention, the modality that is viewed as being most appropriate, design features considered important for the target population, or the potential for the intervention to be scaled up.	P 9 – 11	Supplementary file 3
Report how stakeholders contributed to the intervention development process.	Potential stakeholders can include patient and community representatives, local and national policy makers, health care providers and those paying for or commissioning health care. Each of these groups may influence the intervention development process in different ways. Specifying how differing groups of stakeholders contributed to the intervention development process helps the reader to understand how stakeholders were involved and the degree of influence they had on the overall process. Further detail on how to integrate stakeholder contributions within intervention reporting are available.	P 9 – 11	
Report how the intervention	Intervention development is frequently an iterative process. The conclusion of the initial	P 13 – 18	

For peer review only

changed in content and format from the start of the intervention development process.	phase of intervention development does not necessarily mean that all uncertainties have been addressed. It is helpful to list remaining uncertainties such as the intervention intensity, mode of delivery, materials, procedures, or type of location that the intervention is most suitable for. This can guide other researchers to potential future areas of research and practitioners about uncertainties relevant to their healthcare context.		
Report any changes to the intervention required or likely to be required for subgroups.	Specifying any changes that the intervention development team perceive are required for the intervention to be delivered or tailored to specific sub groups enables readers to understand the applicability of the intervention to their target population or context. These changes could include changes to personnel delivering the intervention, to the content of the intervention, or to the mode of delivery of the intervention.	P 20 – 21	
Report important uncertainties at the end of the development process.	Intervention development is frequently an iterative process. The conclusion of the initial phase of intervention development does not necessarily mean that all uncertainties have been addressed. It is helpful to list remaining uncertainties such as the intervention intensity, mode of delivery, materials, procedures, or type of location that the intervention is most suitable for. This can guide other researchers to potential future areas of research and practitioners about uncertainties relevant to their healthcare context.	P 21 – 22	
Follow TIDieR guidance when describing the developed intervention.	Interventions have been poorly reported for a number of years. In response to this, internationally recognized guidance has been published to support the high quality reporting of health care interventions and public health interventions. This guidance should therefore be followed when describing a developed intervention.		
Brief name.	Provide the name or a phrase that describes the intervention.	P 1	Title page
Rationale, theory and aim of the elements essential to the intervention.	Describe any rationale, theory, or goal of the elements essential to the intervention.	P 13	Supplementary file 3 + [2]
Materials used in the intervention delivery or in the training of intervention providers.	Describe any physical or informational materials used in the intervention, including those provided to participants or used in intervention delivery or in training of intervention providers. Provide information on where the materials can be accessed (e.g. online appendix, URL).	P 13 – 18	
Procedures, activities and/or processes used including enabling	Describe each of the procedures, activities, and/or processes used in the intervention, including any enabling or support activities.	P 13 – 18	

For peer review only

or support activities.			
Expertise and background of intervention providers.	For each category of intervention provider (e.g. psychologist, nursing assistant), describe their expertise, background and any specific training given.	P 14 – 18	
How the intervention was provided.	Describe the modes of delivery (e.g. face-to-face or by some other mechanism, such as internet or telephone) of the intervention and whether it was provided individually or in a group.	P 13 – 18	
Location where the intervention occurred (incl. necessary infrastructure or relevant features).	Describe the type(s) of location(s) where the intervention occurred, including any necessary infrastructure or relevant features.	P 8 – 9	
When and how much the intervention was delivered.	Describe the number of times the intervention was delivered and over what period of time including the number of sessions, their schedule, and their duration, intensity or dose.	P 13 – 18	
Tailoring – what, why, when and how.	If the intervention was planned to be personalised, titrated or adapted, then describe what, why, when, and how.	P 20 – 21	
Modifications. †	If the intervention was modified during the course of the study, describe the changes (what, why, when, and how).	N/A	
Strategies to improve or maintain fidelity.	If intervention adherence or fidelity was assessed, describe how and by whom, and if any strategies were used to maintain or improve fidelity, describe them.	P 20 – 21	
Adherence and fidelity.†	If intervention adherence or fidelity was assessed, describe the extent to which the intervention was delivered as planned.	N/A	
Report the intervention development process in an open access format.	Unless reports of intervention development are available people considering using an intervention cannot understand the process that was undertaken and make a judgement about its appropriateness to their context. It also limits cumulative learning about intervention development methodology and observed consequences at later evaluation, translation and implementation stages. Reporting intervention development in an open access (Gold or Green) publishing format increases the accessibility and visibility of intervention development research and makes it more likely to be read and used. Potential platforms for open access publication of intervention development include open access journal publications, freely accessible funder reports or a study web-page that details the intervention development process.		Aim of this manuscript

Note. * e.g. if item is reported elsewhere, then the location of this information can be stated here. † If completing the TIDieR checklist for a protocol, these items are not relevant to the protocol and cannot be described until the study is complete.

For peer review only

References

1. Mouchaers I, Van Haastregt JCM, Verbeek H, et al. « Reablement » comme outil thérapeutique pour offrir aux personnes âgées une vie de qualité à domicile après une réadaptation gériatrique. Rev Med Brux. 2021;42(4):324-32.
2. Mouchaers I, Verbeek H, Kempen GIJM, et al. The concept of disability and its causal mechanisms in older people over time from a theoretical perspective: a literature review. Eur J Ageing. 2022 2022/01/29.

For peer review only
